# Supplementary material for: Comparative statistical analysis of the release kinetics models for nanoprecipitated drug delivery systems based on poly(lactic-co-glycolic acid)
Source: PLoS One. 2022 Mar 10;17(3):e0264825. doi: 10.1371/journal.pone.0264825 (PMC8912140; doi:10.1371/journal.pone.0264825)
Supplement: S1 File — (DOCX) [file pone.0264825.s001.docx]

**S1 File.**

**S1 Tables.**

**Equations of second to fifth order polynomials.**

| **No. Equation** | **Model** | **Equation** |
| --- | --- | --- |
| (6) | 2nd order polynomial | $Q_{t}=a+bt+ct^{2}$ |
| (7) | 3rd order polynomial | $Q_{t}=a+bt+ct^{2}+dt^{3}$ |
| (8) | 4th order polynomial | $Q_{t}=a+bt+ct^{2}+dt^{3}+et^{4}$ |
| (9) | 5th order polynomial | $Q_{t}=a+bt+ct^{2}+dt^{3}+et^{4}+ft^{5}$ |

Where: a, b, c, d, e, f are constants.

**Equations for the mathematical model acceptance criteria.**

| **Criteria** | **Equation** | **Remarks** |
| --- | --- | --- |
| **Sum Square of Errors** | $SSE=\sum\left( Y_{i}-\hat{Y_{i}} \right)^{2}$ | It represents the sum of the difference between the observed value ($Y_{i}$) minus the estimated value ($\hat{Y_{i}}$) for a number $i$ of observations squared. |
| **Sum Square of residual** | $SSR=\sum\left( \hat{Y_{i}}-\bar{Y} \right)^{2}$ | It represents the difference between the estimated value ($\hat{Y_{i}}$) and the average ($Y$) of the $i$ observations. |
| **Sum Square of Total variation** | $SST=\sum\left( Y_{i}-\bar{Y} \right)^{2}$ | $SST=SSR+SSE$ |
| **Regression coefficient (R^2^)** | $R^{2}=\frac{\sum\left( \hat{Y_{i}}-\bar{Y} \right)^{2}}{\sum\left( Y_{i}-\bar{Y} \right)^{2}}$ | $R^{2}=\frac{SSR}{SST}$  It measures the goodness of fit of a regression model. It has a range between 0 and 1. The closer the coefficient is to one, the better the estimation of the regression model [87]. R^2^_B_: regression coefficient obtained by applying the Bootstrap resampling technique. |
| **Adjusted or corrected regression coefficient (R^2^_a_)** | ${R_{a}}^{2}=1-\left( 1-R^{2} \right)\frac{N-1}{N-k-1}$ | Occasionally, $R^{2}$ of the sample tends to be larger than the $R^{2}$ of the population. In order to rectify this situation, a correction is made to $R^{2}$ generating adjusted $R^{2}$, where $N$ is the sample size and$k$ is the number of variables in the analysis [88]. |
| **Akaike Information Criterion (AIC)** | $AIC=-2\times\ln\left( L \right)+2\times k$ | It allows the selection of a model based on the trade-off between model complexity and predictive ability, so that the goodness of fit measured by maximum likelihood is penalized by the number of parameters involved [89]. $L$ is the likelihood value and $k$ is the number of estimated parameters. The more negative the value of AIC, the greater the adjustment is considered to be [90]. |
| **Bayesian Information Criterion (BIC)** | $BIC=-2\times\ln\left( L \right)+\ln\left( N \right)\times k$ | Sample fit to estimate the model's probability of predicting or estimating future values.  $L$ likelihood value, $N$ is the number of measurements recorded y $k$ is the number of estimated parameters. The more negative the value of AIC, the greater the adjustment is considered to be [91]. |
