# Supplementary material for: Comparative statistical analysis of the release kinetics models for nanoprecipitated drug delivery systems based on poly(lactic-co-glycolic acid)
Source: PLoS One. 2022 Mar 10;17(3):e0264825. doi: 10.1371/journal.pone.0264825 (PMC8912140; doi:10.1371/journal.pone.0264825)
Supplement: S2 File — Forty sets of cumulative drug release data obtained from different scientific articles published in scientific journals since 2016 are listed below. It is included, if available, the model that best fits each set according to the authors. (DOCX) [file pone.0264825.s002.docx]

**S2 File. Release kinetic data.**

| **No.** | **Time**  **[hours]** | **Cumulative release [%]** | **Best fit model proposed by authors** | **Ref.** |
| --- | --- | --- | --- | --- |
| **1** | 1 | 18 | NA | [100] |
|  | 2 | 32 |  |  |
|  | 4 | 50 |  |  |
|  | 8 | 62 |  |  |
|  | 12 | 65 |  |  |
|  | 24 | 70 |  |  |
|  | 72 | 74 |  |  |
|  | 120 | 78 |  |  |
|  | 168 | 80 |  |  |
| **2** | 1 | 21 | NA | [100] |
|  | 2 | 38 |  |  |
|  | 4 | 49 |  |  |
|  | 8 | 56 |  |  |
|  | 12 | 63 |  |  |
|  | 24 | 68 |  |  |
|  | 72 | 70 |  |  |
|  | 120 | 73 |  |  |
|  | 168 | 75 |  |  |
| **3** | 1 | 15 | NA | [101] |
|  | 2 | 20 |  |  |
|  | 5 | 30 |  |  |
|  | 12 | 40 |  |  |
|  | 24 | 44 |  |  |
|  | 35 | 48 |  |  |
|  | 48 | 50 |  |  |
|  | 60 | 52 |  |  |
|  | 72 | 55 |  |  |
|  | 98 | 59 |  |  |
|  | 120 | 62 |  |  |
|  | 240 | 68 |  |  |
| **4** | 48 | 23 | Biphasic: First order + Stationary Estate | [102] |
|  | 120 | 38 |  |  |
|  | 144 | 51 |  |  |
|  | 216 | 60 |  |  |
|  | 504 | 82 |  |  |
| **5** | 48 | 19 | Biphasic: First order + Stationary Estate | [102] |
|  | 120 | 26 |  |  |
|  | 144 | 39 |  |  |
|  | 216 | 47 |  |  |
|  | 504 | 58 |  |  |
| **6** | 0.5 | 10 | Biphasic | [103] |
|  | 1 | 18 |  |  |
|  | 3 | 40 |  |  |
|  | 6 | 52 |  |  |
|  | 12 | 65 |  |  |
|  | 24 | 82 |  |  |
|  | 48 | 90 |  |  |
| **7** | 0.5 | 80 | Biphasic | [103] |
|  | 1 | 92 |  |  |
|  | 3 | 100 |  |  |
|  | 6 | 100 |  |  |
|  | 12 | 100 |  |  |
|  | 24 | 100 |  |  |
|  | 48 | 100 |  |  |
| **8** | 0.5 | 38 | Biphasic | [104] |
|  | 1 | 85 |  |  |
|  | 3 | 100 |  |  |
| **9** | 0.5 | 30 | Biphasic | [104] |
|  | 1 | 73 |  |  |
|  | 3 | 100 |  |  |
| **10** | 1 | 12 | Biphasic | [105] |
|  | 3 | 43 |  |  |
|  | 6 | 70 |  |  |
|  | 12 | 80 |  |  |
|  | 24 | 88 |  |  |
| **11** | 1 | 10 | Biphasic | [105] |
|  | 3 | 24 |  |  |
|  | 6 | 52 |  |  |
|  | 12 | 65 |  |  |
|  | 24 | 79 |  |  |
| **12** | 0.5 | 15 | Biphasic: Burst release + Zero order | [106] |
|  | 1 | 22 |  |  |
|  | 2 | 25 |  |  |
|  | 3 | 50 |  |  |
|  | 6 | 52 |  |  |
|  | 24 | 55 |  |  |
|  | 48 | 79 |  |  |
|  | 96 | 80 |  |  |
|  | 120 | 88 |  |  |
|  | 168 | 100 |  |  |
| **13** | 4 | 12 | Korsmeyer-Peppas | [107] |
|  | 12 | 20 |  |  |
|  | 24 | 26 |  |  |
|  | 48 | 31 |  |  |
|  | 72 | 46 |  |  |
|  | 96 | 49 |  |  |
|  | 120 | 52 |  |  |
|  | 168 | 55 |  |  |
|  | 240 | 57 |  |  |
|  | 288 | 59 |  |  |
|  | 360 | 60 |  |  |
| **14** | 2.5 | 33.5 | Non-fickian behavior | [108] |
|  | 5 | 37 |  |  |
|  | 12 | 54 |  |  |
|  | 24 | 63 |  |  |
|  | 48 | 70 |  |  |
|  | 72 | 79 |  |  |
|  | 120 | 82 |  |  |
|  | 240 | 86 |  |  |
| **15** | 2.5 | 38 | Non-fickian behavior | [108] |
|  | 5 | 44 |  |  |
|  | 12 | 64 |  |  |
|  | 24 | 71 |  |  |
|  | 48 | 77 |  |  |
|  | 72 | 83 |  |  |
|  | 120 | 85 |  |  |
|  | 240 | 88.5 |  |  |
| **16** | 2.5 | 40 | Non-fickian behavior | [108] |
|  | 5 | 47 |  |  |
|  | 12 | 62 |  |  |
|  | 24 | 64 |  |  |
|  | 48 | 70 |  |  |
|  | 72 | 78 |  |  |
|  | 120 | 81 |  |  |
|  | 240 | 87.5 |  |  |
| **17** | 2.5 | 47 | Non-fickian behavior | [108] |
|  | 5 | 55 |  |  |
|  | 12 | 69 |  |  |
|  | 24 | 76 |  |  |
|  | 48 | 79 |  |  |
|  | 72 | 81 |  |  |
|  | 120 | 87 |  |  |
|  | 240 | 89 |  |  |
| **18** | 0.5 | 5 | NA | [109] |
|  | 0.6 | 69 |  |  |
|  | 0.8 | 81 |  |  |
|  | 1 | 97 |  |  |
|  | 2.5 | 98 |  |  |
|  | 3 | 100 |  |  |
| **19** | 0.5 | 3 | NA | [109] |
|  | 0.6 | 30 |  |  |
|  | 0.7 | 61 |  |  |
|  | 0.8 | 82 |  |  |
|  | 1 | 98 |  |  |
|  | 2.5 | 99 |  |  |
|  | 3 | 100 |  |  |
| **20** | 0.5 | 8 | NA | [110] |
|  | 1 | 9 |  |  |
|  | 1.5 | 12 |  |  |
|  | 2 | 14 |  |  |
|  | 3 | 17 |  |  |
|  | 4 | 23 |  |  |
|  | 5 | 25 |  |  |
|  | 6 | 26 |  |  |
|  | 7 | 30 |  |  |
|  | 8 | 31 |  |  |
|  | 9 | 33 |  |  |
|  | 10 | 35 |  |  |
|  | 12 | 36 |  |  |
|  | 24 | 42 |  |  |
| **21** | 0.5 | 10 | NA | [110] |
|  | 1 | 11 |  |  |
|  | 1.5 | 12 |  |  |
|  | 2 | 13 |  |  |
|  | 3 | 15 |  |  |
|  | 4 | 17 |  |  |
|  | 5 | 19 |  |  |
|  | 6 | 21 |  |  |
|  | 7 | 23 |  |  |
|  | 8 | 24 |  |  |
|  | 9 | 26 |  |  |
|  | 10 | 28 |  |  |
|  | 12 | 30 |  |  |
|  | 24 | 36 |  |  |
| **22** | 0.5 | 10 | NA | [110] |
|  | 1 | 12 |  |  |
|  | 1.5 | 13 |  |  |
|  | 2 | 16 |  |  |
|  | 3 | 17 |  |  |
|  | 4 | 19 |  |  |
|  | 5 | 21 |  |  |
|  | 6 | 24 |  |  |
|  | 7 | 25 |  |  |
|  | 8 | 30 |  |  |
|  | 9 | 33 |  |  |
|  | 10 | 34 |  |  |
|  | 12 | 36 |  |  |
|  | 24 | 43 |  |  |
| **23** | 0.5 | 16 | NA | [110] |
|  | 1 | 18 |  |  |
|  | 1.5 | 21 |  |  |
|  | 2 | 23 |  |  |
|  | 3 | 25 |  |  |
|  | 4 | 28 |  |  |
|  | 5 | 31 |  |  |
|  | 6 | 35 |  |  |
|  | 7 | 38 |  |  |
|  | 8 | 39 |  |  |
|  | 9 | 43 |  |  |
|  | 10 | 46 |  |  |
|  | 12 | 50 |  |  |
|  | 24 | 58 |  |  |
| **24** | 0.5 | 11 | NA | [110] |
|  | 1 | 14 |  |  |
|  | 1.5 | 18 |  |  |
|  | 2 | 21 |  |  |
|  | 3 | 21 |  |  |
|  | 4 | 24 |  |  |
|  | 5 | 27 |  |  |
|  | 6 | 28 |  |  |
|  | 7 | 30 |  |  |
|  | 8 | 33 |  |  |
|  | 9 | 35 |  |  |
|  | 10 | 35 |  |  |
|  | 12 | 38 |  |  |
|  | 24 | 44 |  |  |
| **25** | 0.5 | 15 | NA | [110] |
|  | 1 | 16 |  |  |
|  | 1.5 | 21 |  |  |
|  | 2 | 23 |  |  |
|  | 3 | 25 |  |  |
|  | 4 | 28 |  |  |
|  | 5 | 31 |  |  |
|  | 6 | 34 |  |  |
|  | 7 | 38 |  |  |
|  | 8 | 39 |  |  |
|  | 9 | 43 |  |  |
|  | 10 | 46 |  |  |
|  | 12 | 50 |  |  |
|  | 24 | 62 |  |  |
| **26** | 2 | 56 | NA | [111] |
|  | 4 | 65 |  |  |
|  | 6 | 67 |  |  |
|  | 8 | 68 |  |  |
|  | 10 | 70 |  |  |
|  | 12 | 71 |  |  |
|  | 24 | 73 |  |  |
|  | 48 | 75 |  |  |
|  | 144 | 95 |  |  |
| **27** | 2 | 50 | NA | [47] |
|  | 6 | 65 |  |  |
|  | 8 | 73 |  |  |
|  | 24 | 82 |  |  |
|  | 48 | 85 |  |  |
|  | 72 | 92 |  |  |
| **28** | 2 | 23 | NA | [47] |
|  | 6 | 42 |  |  |
|  | 8 | 45 |  |  |
|  | 24 | 74 |  |  |
|  | 48 | 74 |  |  |
|  | 72 | 80 |  |  |
| **29** | 2 | 25 | NA | [47] |
|  | 6 | 45 |  |  |
|  | 8 | 50 |  |  |
|  | 24 | 63 |  |  |
|  | 48 | 68 |  |  |
|  | 72 | 75 |  |  |
| **30** | 2 | 21 | NA | [47] |
|  | 6 | 32 |  |  |
|  | 8 | 35 |  |  |
|  | 24 | 48 |  |  |
|  | 48 | 50 |  |  |
|  | 72 | 57 |  |  |
| **31** | 2 | 32 | Desorption. diffusion. erosion | [112] |
|  | 4 | 42 |  |  |
|  | 8 | 50 |  |  |
|  | 24 | 55 |  |  |
|  | 48 | 63 |  |  |
|  | 72 | 65 |  |  |
|  | 96 | 66 |  |  |
|  | 120 | 68 |  |  |
| **32** | 2 | 30 | Desorption. diffusion. erosion | [112] |
|  | 4 | 41 |  |  |
|  | 8 | 50 |  |  |
|  | 24 | 56 |  |  |
|  | 48 | 64 |  |  |
|  | 72 | 65 |  |  |
|  | 96 | 66.5 |  |  |
|  | 120 | 68.5 |  |  |
| **33** | 0.5 | 8 | NA | [113] |
|  | 1 | 19 |  |  |
|  | 1.5 | 35 |  |  |
|  | 2 | 42 |  |  |
|  | 3 | 51 |  |  |
|  | 4 | 62 |  |  |
| **34** | 1 | 25 | Higuchi | [114] |
|  | 2 | 42 |  |  |
|  | 3 | 49 |  |  |
|  | 4 | 60 |  |  |
|  | 5 | 78 |  |  |
|  | 6 | 80 |  |  |
|  | 12 | 87 |  |  |
|  | 24 | 91 |  |  |
|  | 48 | 92 |  |  |
| **35** | 1 | 28 | Korsmeyer-Peppas | [115] |
|  | 2 | 41 |  |  |
|  | 4 | 55 |  |  |
|  | 6 | 66 |  |  |
|  | 8 | 76 |  |  |
|  | 10 | 82 |  |  |
|  | 12 | 92 |  |  |
|  | 16 | 99 |  |  |
| **36** | 0.5 | 30 | NA | [38] |
|  | 1 | 42 |  |  |
|  | 2 | 76 |  |  |
|  | 4 | 83 |  |  |
|  | 6 | 98 |  |  |
|  | 8 | 100 |  |  |
| **37** | 24 | 17 | NA | [116] |
|  | 48 | 20 |  |  |
|  | 120 | 34 |  |  |
|  | 168 | 39 |  |  |
| **38** | 0.5 | 0 | NA | [46] |
|  | 1 | 1 |  |  |
|  | 1.5 | 9 |  |  |
|  | 3 | 15 |  |  |
|  | 6 | 19 |  |  |
|  | 12 | 28 |  |  |
|  | 24 | 30 |  |  |
| **39** | 0.5 | 0 | NA | [46] |
|  | 1 | 3 |  |  |
|  | 1.5 | 9 |  |  |
|  | 3 | 18 |  |  |
|  | 6 | 22 |  |  |
|  | 12 | 28 |  |  |
|  | 24 | 35 |  |  |
| **40** | 0.5 | 1 | NA | [46] |
|  | 1 | 3 |  |  |
|  | 1.5 | 12 |  |  |
|  | 3 | 19 |  |  |
|  | 6 | 26 |  |  |
|  | 12 | 31 |  |  |
|  | 24 | 38 |  |  |

Forty sets of cumulative drug release data obtained from different scientific articles published in scientific journals since 2016 are listed above. It is included, if available, the model that best fits each set according to the authors.
