# Supplementary material for: Comparative statistical analysis of the release kinetics models for nanoprecipitated drug delivery systems based on poly(lactic-co-glycolic acid)
Source: PLoS One. 2022 Mar 10;17(3):e0264825. doi: 10.1371/journal.pone.0264825 (PMC8912140; doi:10.1371/journal.pone.0264825)
Supplement: S3 File — This appendix shows the programming models in the statistical program R. For this purpose, set number 33 [109] and its fit to the Korsmeyer-Peppas model was taken as an example. (DOCX) [file pone.0264825.s003.docx]

# S3 File. R programming

# This appendix shows the programming models in the statistical program R. For this purpose, set number 33 [111] and its fit to the Korsmeyer-Peppas model was taken as an example.

# First step: Insert the data

In a new script, the cumulative release data were entered in a numeric object (Qt) as well as the time data (t).

Qt<- c(8,19,35,42,51,62)

t <- c(0.5,1,1.5,2,3,4)

The data were placed in the linearly correct form, for the Korsmeyer-Peppas case, the logarithm of both the cumulative release (Qt) and time (t) were calculated.

lnQ<- c(log(Qt))

lnT<- c(log(t))

# Second step: Linear regression

To perform the simple linear regression process, the "lm" function was used, placing first the data of the vertical axis, in this case, Qt, followed by the "~" sign and the data of the horizontal axis.

> reg_lin<-lm(lnQ~lnT)

This function was placed in an object (reg_lin) that can then be called to view the values of the intercept ("Intercept") and slope ("lnT").

> reg_lin

Call:

lm(formula = lnQ ~ lnT)

Coefficients:

(Intercept) lnT

2.9219 0.9841

"Summary" function returns a summary of the regression process:

> summary(reg_lin)

Call:

lm(formula = lnQ ~ lnT)

Residuals:

1 2 3 4 5 6

-0.16029 0.02255 0.23443 0.13363 -0.07125 -0.15906

Coefficients:

Estimate Std. Error t value Pr(>|t|)

(Intercept) 2.92189 0.08952 32.638 5.25e-06 ***

lnT 0.98414 0.10632 9.256 0.000757 ***

---

Signif. codes: 0 ‘***’ 0.001 ‘**’ 0.01 ‘*’ 0.05 ‘.’ 0.1 ‘ ’ 1

Residual standard error: 0.1799 on 4 degrees of freedom

Multiple R-squared: 0.9554, Adjusted R-squared: 0.9442

F-statistic: 85.68 on 1 and 4 DF, p-value: 0.0007574

First, the "*Call*" output is shown, which contains the formula with which the model was performed. The second output, named "*Residuals*", provides the 5 statistics of the distribution of the residuals of the model (minimum value, first, second and third quartiles and maximum value). The third output, "*Coefficients*", contains the coefficients estimated by the model: the ordinate to the origin ("*Intercept*") and the values of the slopes estimated for each variable, in this case, there is only one slope which for Korsmeyer-Peppas is the parameter "*n*", each estimated coefficient ("*Estimate*") is presented together with the standard error of the estimation ("*Std. Error*") or average of the residuals, the t-value ("*t value*") which is a divergence statistic between the estimated coefficient obtained and the coefficient with a value of zero and finally the p-value ("*Pr(>|t|)*") of the hypothesis test of the t-statistic, which shows the probability that the estimated coefficient has a value of zero, if the p-value is greater than 0. 05, the null of the coefficient is accepted, which in the case of the slope, would result in a horizontal line, on the other hand, in the intercept, the null would indicate that the line starts at the origin of the coordinate system. The last output of the summary contains the standard error of the residuals ("*Residual standard error*"), the R2, multiple ("*Multiple R-squared*") and adjusted ("*Adjusted R-squared")* values. The F-statistic and its corresponding p-value are reported, this statistic contrasts the analyzed model with a hypothetical one in which the variables have no effect, i.e. in which all coefficients are equal to zero ^[21]^.

To calculate the sum of squares of residuals (SSR), the residuals of the regression were summed by adding the square of the residuals of the regression

> SSR<- sum((reg_lin$residuals)^2)

> SSR

[1] 0.1293912

Considering the relationship between the sum of squares of the residual, the sum of total squares and R^2^, the value of the sum of total squares (SST) was obtained.

> SST<-SSR/0.9554

> SST

[1] 0.1354314

With the total sum of squares and the sum of squares of the error, the sum of squares of the error (SSE) was calculated.

SSE<-SST-SSR

> SSE

[1] 0.006040242

# Third step: Calculation of AIC and BIC

The “*glance*” function of the library “*broom*” ^[22]^ provides general model information such as the multiple ("*r.squared*") and adjusted ("*adj.r.squared*") R^2^, estimated standard error of the residuals ("*sigma*"), the F-statistic ("*statistic*") followed by its p-value ("*p. value*"), degrees of freedom of the numerator of the F statistic ("*df*"), model likelihood value ("*logLik*"), Akaike information criterion ("*AIC*"), Bayesian information criterion ("*BIC*"), model deviance ("deviance"), residual degrees of freedom ("df.residual") and the number of observations used ("*nobs*") to build the model ^[23]^.

library(broom)

> glance(reg_lin)

# A tibble: 1 x 12

r.squared adj.r.squared sigma statistic p.value df logLik AIC BIC deviance df.residual nobs

<dbl> <dbl> <dbl> <dbl> <dbl> <dbl> <dbl> <dbl> <dbl> <dbl> <int> <int>

0.955 0.944 0.180 85.7 0.000757 1 3.00 0.00722 -0.618 0.129 4 6

# Fourth step: Bootstrap correction of parameters and regression coefficient.

For this purpose, the "*boot*" function was used, which requires the following packages “*car*” ^[24]^, “*carData*” ^[25]^, “*MuMIn*” ^[26]^ y “*boot*” ^[27]^ adjusted to 1000 Bootstrap samples, you must specify that "*f=coef*" in the code to obtain the value of the coefficients:

> library(carData)

> library(car)

> library(MuMIn)

> library(boot)

> kp<-Boot(reg_lin, R=1000,f=coef, method = "case")

> summary(kp,high.moments = FALSE)

Number of bootstrap replications R = 1000

original bootBias bootSE bootMed

(Intercept) 2.92189 0.051374 0.13419 2.93979

lnT 0.98414 -0.029839 0.16517 0.98414

The "*summary*" function was applied to see the summary of this process, obtaining the value of the real estimate ("*original*"), bias error ("*bootBias*"), standard error measured by Bootstrap ("*bootSE*"), and the mean value of the coefficient ("*bootMed*") obtained in the 1000 samples. With the "*confint*" function, the coefficients were checked to ensure that they were within the 95% confidence level.

> confint(kp,level=.95,type="bca")

Bootstrap bca confidence intervals

2.5 % 97.5 %

(Intercept) 2.7627737 3.153772

lnT 0.5635885 1.271625

By changing “*f=coef*” to “*f=MuMIn: :r.squaredGLMM*”, the estimated value of R^2^ was obtained

> rboot<- Boot(reg_lin, R=1000,f=MuMIn::r.squaredGLMM, method = "case")

> summary(rboot)

Number of bootstrap replications R = 999

original bootBias bootSE bootMed

0.94486 0.012665 0.032234 0.95942

In the same way, "*confint*" was applied to obtain the confidence interval

> confint(rboot, level=.95,type="bca")

Bootstrap bca confidence intervals

2.5 % 97.5 %

0.8155992 0.9920776

# Fifth step: AIC Bootstrap Correction

In order to apply Bootstrap in the Akaike information criterion measurement, you will use the "BootStepAIC" function available in the "BootStepAIC" package ^[28]^ which also requires the “*MASS*” library ^[29]^. This function works with objects in "*dataframe*" format, so first the dependent and independent variables were placed in a *dataframe*.

When compiling the function, the program returns firstly the output "*Call*", which shows the model formula, secondly the Bootstrap samples, thirdly the process direction, fourthly the penalty, fifthly the selected covariates, sixthly the sign of the coefficients, seventhly the statistical significance, eighth the original data set ("stepAIC() for the original data-set gave") which includes the model formula and the value of the coefficients estimated by linear regression. The ninth place shows the initial model and the tenth place the final model. In the case of our model, both are the same, however, if any of the parameters is a better fit with a value of zero, we could differentiate them here. Finally, statistics of the final model are shown, such as the degrees of freedom ("*df*"), the deviation of the residuals ("*Resid. Dev.*") and the AIC value.

> Datos<-data.frame(lnQ, lnT)

> library(MASS)

> library(bootStepAIC)

> boot.stepAIC(reg_lin,Datos,B=1000, alpha=0.05)

Summary of Bootstrapping the 'stepAIC()' procedure for

Call:

lm(formula = lnQ ~ lnT)

Bootstrap samples: 1000

Direction: backward

Penalty: 2 * df

Covariates selected

(%)

lnT 100

Coefficients Sign

+ (%) - (%)

lnT 100 0

Stat Significance

(%)

lnT 100

The stepAIC() for the original data-set gave

Call:

lm(formula = lnQ ~ lnT)

Coefficients:

(Intercept) lnT

2.9219 0.9841

Stepwise Model Path

Analysis of Deviance Table

Initial Model:

lnQ ~ lnT

Final Model:

lnQ ~ lnT

Step Df Deviance Resid. Df Resid. Dev AIC

1 4 0.1293912 -19.02005
