# Supplementary material for: Comparative statistical analysis of the release kinetics models for nanoprecipitated drug delivery systems based on poly(lactic-co-glycolic acid)
Source: PLoS One. 2022 Mar 10;17(3):e0264825. doi: 10.1371/journal.pone.0264825 (PMC8912140; doi:10.1371/journal.pone.0264825)
Supplement: S4 File — (DOCX) [file pone.0264825.s004.docx]

**S4 File.**

**Order Zero model by simple linear regression.**

| **No.** | **Inter** | **t-value** | **p-value** | **ES**  **Boot** | **Pend** | **t-value** | **p-value** | **ES**  **Boot** | **Est F** | **p-value** | **SSE** | **SST** | **SSR** | **R^2^** | **R^2^_a_** | **AIC** | **BIC** |
| --- | --- | --- | --- | --- | --- | --- | --- | --- | --- | --- | --- | --- | --- | --- | --- | --- | --- |
| **1** | 47.74 | 6.73 | 2E-4 | 8.59 | 0.24 | 2.50 | 0.04 | 0.40 | 6.27 | 0.04 | 2166.87 | 4109.37 | 1942.5 | 0.47 | 0.40 | 79.9 | 80.5 |
| **2** | 47.66 | 7.97 | 9E-5 | 6.72 | 0.20 | 2.51 | 0.04 | 0.33 | 6.32 | 0.04 | 1527.5 | 2907.86 | 1380.36 | 0.47 | 0.40 | 76.8 | 77.4 |
| **3** | 33.72 | 8.46 | 7E-6 | 5.11 | 0.19 | 4.30 | 1E-3 | 0.09 | 18.49 | 1E-3 | 1163.70 | 1607.40 | 1043.2 | 0.65 | 0.61 | 93.6 | 95.1 |
| **4** | 26.26 | 4.11 | 0.02 | 10.24 | 0.12 | 4.83 | 0.02 | 0.07 | 23.33 | 0.01 | 29.21 | 256.5 | 227.29 | 0.89 | 0.85 | 39.3 | 38.1 |
| **5** | 21.26 | 3.69 | 0.03 | 10.02 | 0.08 | 3.61 | 0.03 | 0.06 | 13.1 | 0.03 | 42.13 | 226.03 | 183.90 | 0.81 | 0.75 | 38.2 | 37.0 |
| **6** | 30.66 | 3.58 | 0.01 | 9.41 | 1.51 | 3.68 | 0.01 | 1.31 | 13.6 | 0.01 | 550.33 | 2047.38 | 1497.04 | 0.73 | 0.68 | 63.4 | 63.3 |
| **7** | 80.38 | 13.18 | 0.04 | 7.32 | 6.86 | 2.07 | 0.28 | 8.04 | 4.32 | 0.28 | 8.82 | 46.91 | 38.095 | 0.81 | 0.64 | 22.1 | 19.4 |
| **8** | 44.55 | 1.68 | 0.34 | 31.15 | 19.86 | 1.39 | 0.39 | 33.21 | 1.94 | 0.39 | 367.91 | 1080.50 | 712.59 | 0.66 | 0.32 | 30.9 | 28.2 |
| **8*** | 0 | - | - | - | 39.41 | 3.40 | 0.07 | 20.45 | 11.6 | 0.07 | 473.51 | 3219.0 | 2745.4 | 0.85 | 0.78 | 33.0 | 31.2 |
| **9** | 31.88 | 1.44 | 0.38 | 26.75 | 23.86 | 1.99 | 0.29 | 28.38 | 3.97 | 0.29 | 125.77 | 626.37 | 500.59 | 0.80 | 0.60 | 29.9 | 27.2 |
| **9*** | 0 | - | - | - | 37.85 | 4.36 | 0.14 | 15.19 | 19.05 | 0.04 | 161.84 | 1541.78 | 1703.62 | 0.90 | 0.86 | 31.2 | 29.4 |
| **10** | 33.44 | 2.40 | 0.09 | 17.58 | 2.73 | 2.43 | 0.09 | 31.63 | 5.89 | 0.09 | 665.70 | 1969.55 | 1303.84 | 0.66 | 0.55 | 48.0 | 46.8 |
| **10*** | 0 | - | - | - | 4.74 | 4.25 | 0.01 | 2.36 | 18.11 | 0.01 | 840.63 | 3806.33 | 4646.97 | 0.81 | 0.77 | 51.4 | 50.6 |
| **11** | 20.39 | 2.13 | 0.12 | 10.95 | 2.78 | 3.61 | 0.03 | 1.70 | 13.03 | 0.03 | 140.64 | 751.69 | 611.05 | 0.81 | 0.75 | 44.2 | 43.0 |
| **11*** | 0 | - | - | - | 4.00 | 5.65 | 4E-3 | 1.47 | 31.92 | 4E-3 | 193.30 | 1541.95 | 1735.26 | 0.88 | 0.86 | 46.8 | 46.1 |
| **12** | 36.36 | 5.95 | 3E-4 | 6.46 | 0.43 | 5.23 | 7E-4 | 0.31 | 27.38 | 7E-4 | 525.41 | 1789.18 | 1789.18 | 0.77 | 0.74 | 86.2 | 87.2 |
| **13** | 26.72 | 6.08 | 1E-4 | 5.02 | 0.12 | 4.76 | 1E-3 | 0.03 | 22.72 | 1E-3 | 329.86 | 1161.49 | 831.62 | 0.72 | 0.68 | 84.8 | 86.0 |
| **14** | 50.33 | 8.02 | 2E-4 | 6.85 | 0.19 | 3.10 | 0.02 | 0.17 | 9.64 | 0.02 | 673.80 | 1756.52 | 1082.72 | 0.62 | 0.55 | 68.0 | 68.2 |
| **15** | 57.60 | 8.86 | 1E-4 | 7.61 | 0.17 | 2.63 | 0.03 | 0.19 | 6.96 | 0.03 | 1001.13 | 2163.2 | 1162.07 | 0.54 | 0.46 | 68.8 | 68.8 |
| **16** | 55.28 | 11.57 | 2E-5 | 5.41 | 0.16 | 3.49 | 0.01 | 0.12 | 12.21 | 0.01 | 935.59 | 627.31 | 308.27 | 0.67 | 0.62 | 63.6 | 63.8 |
| **17** | 63.88 | 12.58 | 1E-5 | 6.08 | 0.13 | 2.71 | 0.03 | 0.13 | 7.35 | 0.03 | 578.41 | 1287.07 | 708.66 | 0.55 | 0.48 | 64.6 | 64.8 |
| **18** | 45.56 | 2.00 | 0.11 | 37.67 | 21.03 | 1.57 | 0.18 | 46.22 | 2.49 | 0.19 | 6532.06 | 10603.99 | 4071.93 | 0.38 | 0.23 | 62.1 | 61.5 |
| **18*** | 0 | - | - | - | 42.89 | 4.44 | 6E-3 | 15.25 | 19.74 | 6E-3 | 2066.11 | 8157.11 | 10223.2 | 0.79 | 0.75 | 64.3 | 63.9 |
| **19** | 34.14 | 1.72 | 0.14 | 41.55 | 25.71 | 2.07 | 0.09 | 52.89 | 4.31 | 0.09 | 5479.68 | 10204.25 | 4724.57 | 0.46 | 0.35 | 71.5 | 71.3 |
| **19*** | 0 | - | - | - | 42.98 | 5.15 | 2E-3 | 12.06 | 26.53 | 2E-3 | 8720.3 | 16239.10 | 7518.70 | 0.81 | 0.78 | 72.7 | 72.6 |
| **20** | 14.01 | 6.50 | 2E-5 | 3.21 | 1.55 | 6.42 | 3E-5 | 0.61 | 41.31 | 3E-5 | 100.24 | 445.33 | 345.08 | 0.77 | 0.76 | 90.6 | 92.5 |
| **21** | 12.33 | 10.95 | 1E-7 | 1.70 | 1.20 | 9.55 | 5E-7 | 0.33 | 91.21 | 5E-7 | 12.38 | 106.46 | 94.08 | 0.88 | 0.87 | 72.4 | 74.3 |
| **22** | 13.63 | 8.60 | 1E-6 | 2.30 | 1.52 | 8.57 | 1E-6 | 0.46 | 73.58 | 1E-6 | 29.53 | 216.07 | 186.53 | 0.86 | 0.85 | 82.0 | 83.9 |
| **23** | 20.84 | 10.84 | 1E-7 | 2.86 | 1.92 | 8.91 | 1E-6 | 0.56 | 79.47 | 1E-6 | 41.46 | 316.07 | 274.61 | 0.87 | 0.86 | 87.4 | 89.3 |
| **24** | 17.79 | 10.4 | 2E-7 | 2.64 | 1.39 | 7.28 | 9E-6 | 0.48 | 53.06 | 9E-6 | 48.82 | 264.78 | 215.96 | 0.82 | 0.80 | 84.0 | 86.0 |
| **25** | 19.65 | 11.08 | 1E-7 | 2.75 | 2.10 | 10.56 | 1E-7 | 0.52 | 111.5 | 2E-7 | 25.16 | 258.90 | 233.73 | 0.90 | 0.89 | 85.1 | 87.1 |
| **26** | 64.96 | 39.03 | 1E-9 | 2.34 | 0.21 | 6.64 | 2E-4 | 0.17 | 44.21 | 2E-4 | 19.11 | 139.79 | 120.68 | 0.86 | 0.84 | 54.9 | 55.5 |
| **27** | 62.01 | 11.91 | 2E-4 | 6.42 | 0.47 | 3.31 | 0.03 | 0.49 | 11.00 | 0.03 | 113.22 | 424.07 | 310.84 | 0.73 | 0.66 | 46.7 | 46.1 |
| **28** | 37.65 | 4.76 | 8E-3 | 9.77 | 0.70 | 3.27 | 0.03 | 0.63 | 10.7 | 0.03 | 267.23 | 982.48 | 715.25 | 0.73 | 0.66 | 51.7 | 51.1 |
| **29** | 39.27 | 6.55 | 3E-3 | 7.19 | 0.56 | 3.47 | 0.02 | 0.57 | 12.08 | 0.02 | 136.51 | 548.23 | 411.72 | 0.75 | 0.69 | 48.4 | 47.8 |
| **30** | 28.95 | 7.53 | 1E-3 | 4.21 | 0.43 | 4.15 | 0.01 | 0.29 | 17.28 | 0.01 | 39.2 | 208.61 | 169.41 | 0.81 | 0.76 | 43.1 | 42.4 |
| **31** | 43.55 | 11.64 | 2E-5 | 4.67 | 0.24 | 4.17 | 5E-3 | 0.14 | 17.41 | 5E-3 | 103.95 | 405.58 | 301.63 | 0.74 | 0.70 | 57.7 | 58.0 |
| **32** | 42.98 | 10.24 | 5E-5 | 5.01 | 0.25 | 3.89 | 7E-3 | 0.08 | 15.2 | 8E-3 | 149.94 | 529.84 | 379.89 | 0.72 | 0.67 | 59.6 | 59.8 |
| **33** | 6.40 | 1.41 | 0.22 | 5.96 | 14.88 | 7.66 | 1E-3 | 3.26 | 58.73 | 1E-3 | 8.73 | 136.95 | 128.22 | 0.94 | 0.92 | 41.4 | 40.8 |
| **33*** | 0 | - | - | - | 17.25 | 15.84 | 1E-5 | 1.46 | 251 | 2E-5 | 3.82 | 196.36 | 192.54 | 0.98 | 0.98 | 41.8 | 41.4 |
| **34** | 54.99 | 6.65 | 2E-4 | 11.31 | 1.03 | 2.33 | 0.05 | 2.05 | 5.46 | 0.05 | 3349.15 | 5962.52 | 2613.37 | 0.44 | 0.36 | 82.6 | 83.2 |
| **35** | 32.98 | 7.85 | 2E-4 | 5.54 | 4.66 | 9.79 | 6E-5 | 0.70 | 95.88 | 6E-5 | 15.83 | 268.82 | 252.98 | 0.94 | 0.93 | 56.3 | 56.6 |
| **36** | 39.45 | 4.18 | 0.01 | 11.44 | 8.86 | 4.19 | 0.01 | 3.56 | 17.62 | 0.01 | 178.88 | 966.9 | 788.0 | 0.81 | 0.77 | 52.3 | 51.7 |
| **37** | 13.07 | 9.39 | 0.01 | 2.18 | 0.16 | 12.28 | 6E-3 | 0.02 | 150.9 | 6E-3 | 0.05 | 4.52 | 4.46 | 0.99 | 0.98 | 17.8 | 15.9 |
| **38** | 6.29 | 1.87 | 0.11 | 3.71 | 1.21 | 3.76 | 0.01 | 1.03 | 14.14 | 0.01 | 80.30 | 306.50 | 226.19 | 0.74 | 0.69 | 50.2 | 50.0 |
| **38*** | 0 | - | - | - | 1.59 | 5.53 | 1E-3 | 0.76 | 30.6 | 1E-3 | 75.35 | 384.95 | 460.31 | 0.83 | 0.81 | 51.9 | 51.8 |
| **39** | 7.22 | 2.15 | 0.08 | 3.73 | 1.34 | 4.19 | 8E-3 | 1.08 | 17.55 | 8E-3 | 64.26 | 289.87 | 225.61 | 0.78 | 0.73 | 50.2 | 50.0 |
| **39*** | 0 | - | - | - | 1.79 | 5.84 | 1E-3 | 0.86 | 34.1 | 1E-3 | 76.28 | 434.31 | 510.59 | 0.85 | 0.82 | 52.8 | 52.7 |
| **40** | 8.78 | 2.32 | 0.06 | 4.15 | 1.42 | 3.95 | 0.01 | 1.18 | 15.61 | 0.01 | 92.04 | 378.77 | 286.73 | 0.76 | 0.71 | 51.9 | 51.7 |
| **40*** | 0 | - | - | - | 1.97 | 5.49 | 1E-3 | 2.00 | 30.23 | 1E-3 | 118.53 | 595.55 | 714.09 | 0.83 | 0.81 | 55.0 | 54.9 |

Note: * refers to sets that have been rescaled considering the intercept with a value of zero.

**First order model by simple linear regression.**

| **No.** | **Inter** | **t-value** | **p-value** | **ES**  **Boot** | **Pend** | **t-value** | **p-value** | **ES**  **Boot** | **Est F** | **p-value** | **SSE** | **SSR** | **SST** | **R^2^** | **R^2^_a_** | **AIC** | **BIC** |
| --- | --- | --- | --- | --- | --- | --- | --- | --- | --- | --- | --- | --- | --- | --- | --- | --- | --- |
| **1** | 3.76 | 20.37 | 1E-7 | 0.20 | -4E-3 | -1.88 | 0.10 | 5E-3 | 3.56 | 0.10 | 2.59 | 1.32 | 3.92 | 0.34 | 0.24 | 14.3 | 14.9 |
| **2** | 3.79 | 25.02 | 4E-8 | 0.17 | -4E-3 | -1.93 | 0.09 | 7E-3 | 3.75 | 0.09 | 1.65 | 0.89 | 2.54 | 0.35 | 0.26 | 10.7 | 11.3 |
| **3** | 3.44 | 25.02 | 2E-10 | 0.17 | -4E-3 | -3.03 | 0.01 | 3E-3 | 9.19 | 0.01 | 1.35 | 1.24 | 2.60 | 0.48 | 0.43 | 12.9 | 14.3 |
| **4** | 3.35 | 16.18 | 5E-4 | 0.30 | -2E-3 | -2.95 | 0.05 | 2E-3 | 8.71 | 0.05 | 0.08 | 0.23 | 0.32 | 0.74 | 0.66 | 4.98 | 3.81 |
| **5** | 3.11 | 15.04 | 6E-4 | 0.32 | -2E-3 | -2.70 | 0.07 | 2E-3 | 7.29 | 0.07 | 0.09 | 0.23 | 0.33 | 0.71 | 0.61 | 4.96 | 3.78 |
| **6** | 3.24 | 10.44 | 1E-4 | 0.34 | -0.03 | -2.28 | 0.07 | 0.04 | 5.22 | 0.07 | 1.89 | 1.97 | 3.86 | 0.51 | 0.41 | 17.0 | 16.8 |
| **7** | 4.38 | 60.53 | 0.01 | 0.08 | -0.07 | -1.92 | 0.30 | 0.09 | 3.72 | 0.30 | 1E-3 | 5E-3 | 6E-3 | 0.79 | 0.58 | -4.45 | -7.16 |
| **8** | 3.77 | 8.10 | 0.07 | 0.56 | -0.29 | -1.18 | 0.44 | 0.60 | 1.41 | 0.44 | 0.15 | 0.22 | 0.38 | 0.58 | 0.17 | 6.71 | 4.01 |
| **8*** | 0 | - | - | - | -1.95 | -2.30 | 0.14 | 1.62 | 5.29 | 0.14 | 5.60 | 14.85 | 20.45 | 0.72 | 0.58 | 17.3 | 15.5 |
| **9** | 3.51 | 7.11 | 0.08 | 0.56 | -0.38 | -1.45 | 0.38 | 0.61 | 2.11 | 0.38 | 0.11 | 0.25 | 0.36 | 0.68 | 0.36 | 7.06 | 4.36 |
| **9*** | 0 | - | - | - | -1.93 | -2.43 | 0.13 | 1.53 | 5.92 | 0.13 | 4.35 | 12.91 | 17.26 | 0.74 | 0.62 | 16.9 | 15.1 |
| **10** | 3.30 | 7.25 | 5E-3 | 0.54 | -0.06 | -1.68 | 0.19 | 0.09 | 2.84 | 0.19 | 1.46 | 1.38 | 2.85 | 0.49 | 0.32 | 13.8 | 12.6 |
| **11** | 2.92 | 7.33 | 5E-3 | 0.47 | -0.07 | -2.27 | 0.10 | 0.07 | 5.18 | 0.11 | 0.61 | 1.06 | 1.68 | 0.63 | 0.51 | 12.5 | 11.3 |
| **12** | 3.48 | 19.01 | 6E-8 | 0.20 | -8E-3 | -3.31 | 0.01 | 0.01 | 10.99 | 0.01 | 1.17 | 1.61 | 2.78 | 0.58 | 0.53 | 16.1 | 17.0 |
| **13** | 3.20 | 19.42 | 1E-8 | 0.20 | -3E-3 | -3.57 | 6E-3 | 1E-3 | 12.74 | 6E-3 | 0.82 | 1.17 | 1.99 | 0.59 | 0.54 | 12.6 | 13.8 |
| **14** | 3.88 | 30.90 | 7E-8 | 0.14 | -3E-3 | -2.57 | 0.04 | 3E-3 | 6.63 | 0.04 | 0.39 | 0.43 | 0.82 | 0.52 | 0.45 | 5.39 | 5.62 |
| **15** | 4.01 | 34.20 | 4E-8 | 0.13 | -2E-3 | -2.25 | 0.06 | 2E-3 | 5.08 | 0.06 | 0.44 | 0.37 | 0.82 | 0.46 | 0.37 | 4.32 | 4.56 |
| **16** | 3.99 | 44.74 | 8E-9 | 0.10 | -2E-3 | -2.85 | 0.03 | 2E-3 | 8.16 | 0.02 | 0.16 | 0.21 | 0.38 | 0.58 | 0.51 | -0.07 | 0.16 |
| **17** | 4.14 | 50.20 | 4E-9 | 0.09 | -1E-3 | -2.36 | 0.05 | 2E-3 | 5.60 | 0.05 | 0.20 | 0.18 | 0.38 | 0.48 | 0.40 | -1.34 | -1.11 |
| **18** | 3.24 | 3.95 | 0.01 | 1.37 | -0.541 | -1.13 | 0.32 | 1.63 | 1.27 | 0.32 | 16.54 | 5.27 | 21.82 | 0.24 | 0.05 | 22.3 | 21.6 |
| **19** | 2.97 | 3.85 | 0.01 | 1.56 | -0.655 | -1.35 | 0.23 | 1.98 | 1.847 | 0.23 | 19.40 | 7.16 | 26.56 | 0.27 | 0.12 | 26.0 | 25.9 |
| **20** | 2.61 | 19.00 | 2E-10 | 0.19 | -0.06 | -4.47 | 7E-4 | 0.03 | 20.05 | 7E-4 | 0.84 | 1.40 | 2.24 | 0.63 | 0.59 | 13.6 | 15.5 |
| **21** | 2.55 | 31.95 | 5E-13 | 0.12 | -0.05 | -6.40 | 3E-5 | 0.02 | 40.97 | 3E-5 | 0.13 | 0.47 | 0.61 | 0.77 | 0.75 | -1.61 | 0.30 |
| **22** | 2.65 | 28.11 | 2E-12 | 0.14 | -0.06 | -5.94 | 6E-5 | 0.02 | 35.28 | 6E-5 | 0.22 | 0.66 | 0.89 | 0.75 | 0.72 | 3.08 | 4.99 |
| **23** | 3.07 | 37.97 | 7E-14 | 0.12 | -0.05 | -6.14 | 4E-5 | 0.02 | 37.81 | 4E-5 | 0.15 | 0.48 | 0.64 | 0.76 | 0.74 | -1.29 | 0.62 |
| **24** | 2.88 | 30.31 | 1E-12 | 0.14 | -0.05 | -4.92 | 3E-4 | 0.02 | 24.25 | 3E-4 | 0.33 | 0.67 | 1.00 | 0.67 | 0.64 | 3.21 | 5.13 |
| **25** | 3.03 | 35.53 | 1E-13 | 0.12 | -0.06 | -6.30 | 3E-5 | 0.02 | 39.77 | 3E-5 | 0.16 | 0.54 | 0.70 | 0.77 | 0.75 | 0.19 | 2.12 |
| **26** | 4.17 | 152.16 | 1E-13 | 0.03 | -2E-3 | -5.15 | 1E-3 | 2E-3 | 26.62 | 1E-3 | 8E-3 | 0.03 | 0.04 | 0.79 | 0.76 | -19.0 | -18.4 |
| **27** | 4.11 | 47.93 | 1E-6 | 0.10 | -6E-3 | -2.76 | 0.05 | 8E-3 | 7.66 | 0.05 | 0.04 | 0.08 | 0.12 | 0.66 | 0.57 | -2.54 | -3.16 |
| **28** | 3.58 | 18.34 | 5E-5 | 0.23 | 0.01 | -2.58 | 0.06 | 0.01 | 6.68 | 0.06 | 0.26 | 0.43 | 0.69 | 0.63 | 0.53 | 7.31 | 6.68 |
| **29** | 3.63 | 22.01 | 2E-5 | 0.20 | -0.01 | -2.49 | 0.06 | 0.01 | 6.22 | 0.06 | 0.20 | 0.31 | 0.51 | 0.61 | 0.51 | 5.31 | 4.69 |
| **30** | 3.35 | 25.35 | 1E-5 | 0.15 | -0.01 | -3.09 | 0.03 | 9E-3 | 9.60 | 0.03 | 0.08 | 0.20 | 0.28 | 0.71 | 0.63 | 2.61 | 2.01 |
| **31** | 3.75 | 42.72 | 1E-8 | 0.10 | -4E-3 | -3.43 | 0.01 | 1E-3 | 11.76 | 0.01 | 0.08 | 0.16 | 0.25 | 0.66 | 0.61 | -2.25 | -2.02 |
| **32** | 3.73 | 37.08 | 2E-8 | 0.12 | -5E-3 | -3.18 | 0.01 | 2E-3 | 10.16 | 0.02 | 0.12 | 0.21 | 0.34 | 0.63 | 0.57 | -0.07 | 0.16 |
| **33** | 2.37 | 7.21 | 1E-3 | 0.44 | -0.51 | -3.61 | 0.02 | 0.24 | 13.06 | 0.02 | 0.20 | 0.68 | 0.88 | 0.77 | 0.71 | 9.96 | 9.34 |
| **34** | 3.93 | 23.98 | 5E-8 | 0.22 | -0.01 | -1.91 | 0.09 | 0.03 | 2.67 | 0.97 | 1.95 | 1.02 | 2.98 | 0.34 | 0.25 | 12.0 | 12.6 |
| **35** | 3.56 | 29.53 | 9E-8 | 0.16 | -0.07 | -5.62 | 1E-3 | 0.01 | 31.61 | 1E-3 | 0.03 | 0.20 | 0.24 | 0.84 | 0.81 | -0.42 | -0.18 |
| **36** | 3.67 | 18.63 | 4E-5 | 0.25 | -0.14 | -3.23 | 0.03 | 0.07 | 10.44 | 0.03 | 0.13 | 0.33 | 0.47 | 0.72 | 0.65 | 5.80 | 5.18 |
| **37** | 2.71 | 36.70 | 7E-4 | 0.11 | -6E-3 | -8.66 | 0.01 | 1E-3 | 75.12 | 0.01 | 3E-4 | 0.01 | 0.01 | 0.97 | 0.96 | -5.69 | -7.53 |
| **38** | 2.73 | 14.10 | 3E-5 | 0.22 | -0.05 | -2.73 | 0.04 | 0.07 | 7.45 | 0.04 | 0.50 | 0.75 | 1.25 | 0.60 | 0.52 | 10.2 | 10.1 |
| **39** | 2.79 | 14.63 | 2E-5 | 0.22 | -0.05 | -2.83 | 0.03 | 0.06 | 8.02 | 0.03 | 0.45 | 0.73 | 1.18 | 0.62 | 0.54 | 10.1 | 9.89 |
| **40** | 1.68 | 3.02 | 0.03 | 0.66 | -0.10 | -1.96 | 0.10 | 0.20 | 3.87 | 0.10 | 8.07 | 6.25 | 14.32 | 0.43 | 0.32 | 25.2 | 24.9 |

Note: * refers to sets that have been rescaled considering the intercept with a value of zero.

**Korsmeyer Peppas model by analyzing 100% of the release curve by simple linear regression.**

| **No.** | **Obs** | **Inter** | **t-value** | **p-value** | **ES**  **Boot** | **Pend** | **t-value** | **p-value** | **ES**  **Boot** | **Estad F** | **p-value** | **SSE** | **SSR** | **SST** | **R^2^** | **R^2^_a_** | **AIC** | **BIC** |
| --- | --- | --- | --- | --- | --- | --- | --- | --- | --- | --- | --- | --- | --- | --- | --- | --- | --- | --- |
| **1** | 9 | 3.34 | 20.74 | 1E-7 | 0.23 | 0.23 | 4.71 | 2E-3 | 0.06 | 22.21 | 2E-3 | 0.15 | 0.47 | 0.62 | 0.76 | 0.73 | 5.13 | 5.72 |
| **2** | 9 | 3.44 | 26.25 | 2E-8 | 0.19 | 0.19 | 4.81 | 1E-3 | 0.06 | 23.2 | 1E-3 | 0.09 | 0.31 | 0.41 | 0.77 | 0.73 | 1.43 | 2.03 |
| **3** | 12 | 2.86 | 48.52 | 3E-13 | 0.11 | 0.26 | 16.49 | 1E-8 | 0.02 | 271.9 | 1E-8 | 3E-3 | 0.08 | 0.08 | 0.96 | 0.96 | -19.4 | -17.9 |
| **4** | 5 | 1.06 | 3.22 | 0.04 | 0.83 | 0.55 | 8.54 | 3E-3 | 0.16 | 73.09 | 3E-3 | 1E-3 | 0.03 | 0.03 | 0.96 | 0.95 | -4.38 | -5.55 |
| **5** | 5 | 1.02 | 2.19 | 0.11 | 1.05 | 0.50 | 5.48 | 0.01 | 0.21 | 30.04 | 0.01 | 7E-3 | 0.07 | 0.08 | 0.91 | 0.88 | -0.87 | -2.05 |
| **5*** | 5 | 0 | - | - | - | 0.70 | 36.4 | 3E-6 | 0.01 | 1325 | 3E-6 | 5E-4 | 0.19 | 0.19 | 0.997 | 0.996 | 1.91 | 1.13 |
| **6** | 7 | 2.89 | 23.22 | 2E-6 | 0.18 | 0.47 | 8.72 | 3E-4 | 0.07 | 76.09 | 3E-4 | 0.01 | 0.24 | 0.26 | 0.94 | 0.93 | 2.51 | 2.34 |
| **7** | 7 | 4.48 | 176.68 | 3E-3 | 0.02 | 0.12 | 3.54 | 0.17 | 0.04 | 12.58 | 0.17 | 1E-4 | 1E-3 | 0.02 | 0.93 | 0.85 | -7.62 | -10.3 |
| **8** | 3 | 4.16 | 20.32 | 0.03 | 0.18 | 0.50 | 1.84 | 0.31 | 0.35 | 3.41 | 0.31 | 0.03 | 0.12 | 0.15 | 0.77 | 0.55 | 4.90 | 2.19 |
| **9** | 3 | 4.01 | 19.93 | 0.03 | 0.18 | 0.63 | 2.6 | 0.25 | 0.36 | 5.63 | 0.25 | 0.02 | 0.11 | 0.13 | 0.85 | 0.69 | 4.80 | 2.09 |
| **10** | 5 | 2.81 | 9.57 | 2E-3 | 0.44 | 0.61 | 4.29 | 0.02 | 0.21 | 18.42 | 0.02 | 0.06 | 0.37 | 0.44 | 0.86 | 0.81 | 7.29 | 6.12 |
| **11** | 5 | 2.44 | 12.81 | 1E-3 | 0.30 | 0.67 | 7.18 | 5E-3 | 0.14 | 51.58 | 5E-3 | 9E-3 | 0.15 | 0.16 | 0.94 | 0.93 | 2.97 | 1.80 |
| **12** | 10 | 3.16 | 29.28 | 2E-9 | 0.13 | 0.29 | 8.52 | 2E-5 | 0.03 | 72.72 | 2E-5 | 0.04 | 0.37 | 0.42 | 0.90 | 0.88 | 1.66 | 2.57 |
| **13** | 11 | 2.07 | 18.72 | 1E-8 | 0.15 | 0.36 | 14.83 | 1E-7 | 0.03 | 219.8 | 1E-7 | 4E-3 | 0.11 | 0.11 | 0.96 | 0.96 | -13.3 | -12.1 |
| **14** | 8 | 3.35 | 44.26 | 8E-9 | 0.10 | 0.22 | 10.72 | 3E-5 | 0.02 | 114.9 | 3E-5 | 2E-3 | 0.04 | 0.04 | 0.95 | 0.94 | -12.7 | -12.4 |
| **15** | 8 | 3.55 | 40.49 | 1E-8 | 0.12 | 0.19 | 7.94 | 2E-4 | 0.03 | 63.07 | 2E-4 | 5E-3 | 0.06 | 0.06 | 0.91 | 0.90 | -10.3 | -10.1 |
| **16** | 8 | 3.60 | 69.62 | 5E-10 | 0.07 | 0.16 | 11.86 | 2E-5 | 0.01 | 140.7 | 2E-5 | 9E-4 | 0.02 | 0.02 | 0.96 | 0.95 | -18.8 | -18.5 |
| **17** | 8 | 3.80 | 63.87 | 9E-10 | 0.09 | 0.13 | 8.43 | 1E-4 | 0.02 | 71.2 | 1E-4 | 2E-3 | 0.02 | 0.03 | 0.92 | 0.91 | -16.5 | -16.3 |
| **18** | 6 | 3.90 | 8.99 | 8E-4 | 0.57 | 0.95 | 1.50 | 0.20 | 1.12 | 2.27 | 0.20 | 7.79 | 4.43 | 12.23 | 0.36 | 0.20 | 21.2 | 20.6 |
| **19** | 6 | 3.79 | 9.28 | 2E-4 | 0.59 | 1.16 | 1.85 | 0.12 | 1.29 | 3.43 | 0.12 | 8.48 | 5.81 | 14.30 | 0.40 | 0.29 | 24.6 | 24.4 |
| **20** | 14 | 2.34 | 53.04 | 1E-15 | 0.06 | 0.49 | 20.18 | 1E-10 | 0.03 | 407.1 | 1E-10 | 3E-3 | 0.10 | 0.11 | 0.97 | 0.97 | -22.4 | -20.5 |
| **21** | 14 | 2.39 | 66.10 | <2E-16 | 0.06 | 0.37 | 18.34 | 3E-10 | 0.03 | 336.5 | 3E-10 | 2E-3 | 0.07 | 0.07 | 0.97 | 0.96 | -28.0 | -26.1 |
| **22** | 14 | 2.47 | 61.85 | <2E-16 | 0.05 | 0.41 | 18.60 | 3E-10 | 0.02 | 346.0 | 3E-10 | 3E-3 | 0.08 | 0.09 | 0.97 | 0.96 | -25.3 | -23.3 |
| **23** | 14 | 2.90 | 94.55 | <2E-16 | 0.04 | 0.36 | 21.29 | 6E-11 | 0.02 | 453.1 | 6E-11 | 1E-3 | 0.05 | 0.05 | 0.97 | 0.97 | -32.6 | -30.7 |
| **24** | 14 | 2.69 | 112.19 | <2E-16 | 0.03 | 0.36 | 27.49 | 3E-12 | 0.01 | 755.9 | 3E-12 | 5E-4 | 0.03 | 0.03 | 0.98 | 0.98 | -39.5 | -37.6 |
| **25** | 14 | 2.85 | 94.85 | <2E-16 | 0.04 | 0.39 | 23.43 | 2E-11 | 0.02 | 549 | 2E-11 | 1E-3 | 0.04 | 0.05 | 0.98 | 0.98 | -33.2 | -31.2 |
| **26** | 9 | 3.99 | 120.86 | 7E-13 | 0.04 | 0.10 | 8.75 | 5E-5 | 0.01 | 76.56 | 5E-15 | 1E-3 | 0.01 | 0.01 | 0.92 | 0.90 | -27.2 | -26.6 |
| **27** | 6 | 3.87 | 62.53 | 3E-7 | 0.09 | 0.15 | 7.42 | 1E-3 | 0.03 | 55.17 | 1E-3 | 1E-3 | 0.01 | 0.01 | 0.93 | 0.91 | -12.3 | -12.9 |
| **28** | 6 | 3.04 | 22.43 | 2E-5 | 0.19 | 0.34 | 7.37 | 1E-3 | 0.06 | 54.43 | 1E-3 | 5E-3 | 0.07 | 0.08 | 0.93 | 0.91 | -2.89 | -3.51 |
| **29** | 6 | 3.19 | 24.86 | 1E-5 | 0.19 | 0.27 | 6.37 | 3E-3 | 0.06 | 40.6 | 3E-3 | 7E-3 | 0.07 | 0.07 | 0.91 | 0.88 | -3.53 | -4.15 |
| **30** | 6 | 2.94 | 39.27 | 2E-6 | 0.11 | 0.26 | 10.37 | 4E-4 | 0.03 | 107.5 | 4E-4 | 9E-4 | 0.02 | 0.02 | 0.96 | 0.95 | -9.99 | -10.6 |
| **31** | 8 | 3.46 | 59.09 | 1E-9 | 0.09 | 0.16 | 9.73 | 6E-5 | 0.02 | 94.78 | 6E-5 | 1E-3 | 0.02 | 0.03 | 0.94 | 0.93 | -16.1 | -15.9 |
| **32** | 8 | 3.41 | 47.41 | 5E-9 | 0.11 | 0.18 | 8.58 | 1E-4 | 0.03 | 73.7 | 1E-4 | 3E-3 | 0.04 | 0.04 | 0.92 | 0.91 | -12.8 | -12.6 |
| **33** | 6 | 2.92 | 32.63 | 5E-6 | 0.13 | 0.98 | 9.25 | 7E-4 | 0.16 | 85.68 | 7E-4 | 6E-3 | 0.12 | 0.13 | 0.95 | 0.94 | 0.01 | -0.61 |
| **34** | 9 | 3.55 | 25.33 | 3E-8 | 0.19 | 0.31 | 4.83 | 1E-3 | 0.10 | 23.37 | 1E-3 | 0.10 | 0.36 | 0.46 | 0.77 | 0.74 | 2.61 | 3.20 |
| **35** | 8 | 3.36 | 164.09 | 3E-12 | 0.03 | 0.45 | 42.46 | 1E-8 | 0.01 | 1803 | 1E-8 | 3E-5 | 4E-3 | 4E-3 | 0.99 | 0.97 | -31.4 | -31.2 |
| **36** | 6 | 3.78 | 50.48 | 9E-7 | 0.12 | 0.44 | 7.81 | 1E-3 | 0.08 | 61.05 | 1E-3 | 0.01 | 0.07 | 0.09 | 0.94 | 0.92 | -3.23 | -3.85 |
| **37** | 4 | 1.35 | 6.04 | 0.02 | 0.30 | 0.44 | 8.66 | 0.01 | 0.07 | 75.15 | 0.01 | 3E-4 | 0.01 | 0.01 | 0.97 | 0.96 | -5.69 | -7.53 |
| **38** | 7 | 2.62 | 30.34 | 7E-7 | 0.1 | 0.38 | 7.81 | 5E-4 | 0.05 | 60.95 | 5E-4 | 0.01 | 0.14 | 0.15 | 0.92 | 0.91 | -1.42 | -1.59 |
| **39** | 7 | 2.68 | 36.01 | 3E-7 | 0.08 | 0.39 | 9.23 | 2E-4 | 0.05 | 85.2 | 2E-4 | 6E-3 | 0.1 | 0.11 | 0.94 | 0.93 | -3.5 | -3.66 |
| **40** | 7 | 1.36 | 4.20 | 8E-3 | 0.42 | 0.88 | 4.78 | 4E-3 | 0.28 | 22.92 | 4E-3 | 0.43 | 1.98 | 2.42 | 0.82 | 0.78 | 17.0 | 16.9 |

Note: * refers to sets that have been rescaled considering the intercept with a value of zero.

**Korsmeyer Peppas model analyzing 60% of the release curve by simple linear regression.**

| **No.** | **Obs** | **%**  **Release** | **Inter** | **t-**  **valor** | **p-value** | **ES Boot** | **Pend** | **t-value** | **p-value** | **ES Boot** | **Est F** | **p-value** | **SSE** | **SSR** | **SST** | **R^2^** | **R^2^_a_** | **AIC** | **BIC** |
| --- | --- | --- | --- | --- | --- | --- | --- | --- | --- | --- | --- | --- | --- | --- | --- | --- | --- | --- | --- |
| **1** | 4 | 62 | 2.97 | 27.69 | 1E-3 | 0.15 | 0.59 | 7.23 | 0.01 | 0.12 | 52.41 | 0.01 | 1E-3 | 0.03 | 0.03 | 0.96 | 0.94 | -1.84 | -3.68 |
| **2** | 5 | 63 | 3.20 | 26.46 | 1E-4 | 0.17 | 0.41 | 5.47 | 0.01 | 0.11 | 29.92 | 0.01 | 6E-3 | 0.06 | 0.07 | 0.90 | 0.88 | -1.2 | -2.37 |
| **3** | 11 | 62 | 2.83 | 50.07 | 2E-12 | 0.10 | 0.28 | 16.78 | 4E-8 | 0.02 | 281.4 | 4E-8 | 2E-3 | 0.06 | 0.06 | 0.97 | 0.97 | -19.4 | -17.9 |
| **4** | 4 | 60 | 0.60 | 1.56 | 0.25 | 1.06 | 0.65 | 8.12 | 0.01 | 0.21 | 66.07 | 0.01 | 4E-4 | 0.01 | 0.01 | 0.97 | 0.96 | -4.83 | -6.67 |
| **4*** | 4 | 60 | 0 | - | - | - | 0.77 | 69.1 | 6E-6 | 9E-3 | 4770 | 6E-6 | 2E-5 | 0.03 | 0.03 | 0.99 | 0.99 | -3.63 | -4.86 |
| **5** | 5 | 58 | 1.02 | 2.19 | 0.11 | 1.05 | 0.50 | 5.48 | 0.01 | 0.21 | 30.04 | 0.01 | 7E-3 | 0.07 | 0.08 | 0.91 | 0.88 | -0.87 | -2.05 |
| **5*** | 5 | 58 | 0 | - | - | - | 0.70 | 36.4 | 3E-6 | 0.01 | 1325 | 3E-6 | 5E-4 | 0.19 | 0.19 | 0.99 | 0.99 | 1.91 | 1.13 |
| **6** | 5 | 65 | 2.84 | 30.45 | 7E-5 | 0.12 | 0.59 | 9.49 | 2E-3 | 0.09 | 90.1 | 2E-3 | 2E-3 | 0.07 | 0.08 | 0.97 | 0.96 | -0.55 | -1.72 |
| **7** | 3 | 100 | - | - | - | - | - | - | - | - | - | - | - | - | - | - | - | - | - |
| **8** | 3 | 100 | - | - | - | - | - | - | - | - | - | - | - | - | - | - | - | - | - |
| **9** | 3 | 100 | - | - | - | - | - | - | - | - | - | - | - | - | - | - | - | - | - |
| **10** | 2 | 43 | - | - | - | - | - | - | - | - | - | - | - | - | - | - | - | - | - |
| **11** | 4 | 65 | 2.34 | 15.61 | 4E-3 | 0.25 | 0.78 | 8.57 | 0.01 | 0.14 | 73.46 | 0.01 | 1E-4 | 0.05 | 0.05 | 0.97 | 0.96 | 0.35 | -1.48 |
| **12** | 6 | 55 | 3.11 | 21.40 | 2E-5 | 0.14 | 0.35 | 3.94 | 0.01 | 0.12 | 15.53 | 0.01 | 0.07 | 0.30 | 0.38 | 0.79 | 0.74 | 5.20 | 4.58 |
| **13** | 11 | 60 | 2.07 | 18.72 | 1E-8 | 0.15 | 0.36 | 14.83 | 1E-7 | 0.03 | 219.8 | 1E-7 | 4E-3 | 0.11 | 0.11 | 0.96 | 0.96 | -13.3 | -12.1 |
| **14** | 8 | 63 | 3.20 | 37.91 | 6E-4 | 0.11 | 0.29 | 7.86 | 0.01 | 0.05 | 61.84 | 0.01 | 2E-4 | 8E-3 | 8E-3 | 0.97 | 0.95 | -7.26 | -9.10 |
| **15** | 3 | 64 | 3.29 | 30.03 | 0.02 | 0.12 | 0.33 | 5.48 | 0.11 | 0.07 | 30.05 | 0.11 | 4E-4 | 4E-3 | 5E-3 | 0.97 | 0.94 | -4.89 | -7.60 |
| **16** | 3 | 62 | 3.41 | 80.51 | 7E-3 | 0.04 | 0.28 | 11.84 | 0.05 | 0.02 | 140.1 | 0.05 | 4E-6 | 6E-4 | 7E-4 | 0.99 | 0.98 | -10.6 | -13.3 |
| **17** | 3 | 69 | 3.62 | 221.2 | 2E-3 | 0.01 | 0.24 | 26.83 | 0.02 | 0.01 | 719 | 0.02 | 1E-7 | 1E-4 | 1E-4 | 0.99 | 0.99 | -16.3 | -19.0 |
| **18** | 2 | 69 | - | - | - | - | - | - | - | - | - | - | - | - | - | - | - | - | - |
| **19** | 3 | 61 | 1.35 | 6.04 | 0.10 | 0.29 | 0.44 | 8.66 | 0.15 | 0.06 | 75.15 | 0.01 | 3E-4 | 0.01 | 0.01 | 0.97 | 0.96 | -5.69 | -7.53 |
| **19*** | 3 | 61 | 0 | - | - | - | 1.57 | 7.54 | 4E-6 | 0.13 | 56.89 | 4E-6 | 5.78 | 25.3 | 31.1 | 0.81 | 0.79 | 52.0 | 53.3 |
| **20** | 14 | 42 | 2.34 | 53.04 | 1E-15 | 0.06 | 0.49 | 20.18 | 1E-10 | 0.03 | 407.1 | 1E-10 | 3E-3 | 0.10 | 0.11 | 0.97 | 0.97 | -22.4 | -20.5 |
| **21** | 14 | 36 | 2.39 | 66.10 | <2E-16 | 0.06 | 0.37 | 18.34 | 3E-10 | 0.03 | 336.5 | 3E-10 | 2E-3 | 0.07 | 0.07 | 0.97 | 0.96 | -28.0 | -26.1 |
| **22** | 14 | 43 | 2.47 | 61.85 | <2E-16 | 0.05 | 0.41 | 18.60 | 3E-10 | 0.02 | 346.0 | 3E-10 | 3E-3 | 0.08 | 0.09 | 0.97 | 0.96 | -25.3 | -23.3 |
| **23** | 14 | 58 | 2.90 | 94.55 | <2E-16 | 0.04 | 0.36 | 21.29 | 6E-11 | 0.02 | 453.1 | 6E-11 | 1E-3 | 0.05 | 0.05 | 0.97 | 0.97 | -32.6 | -30.7 |
| **24** | 14 | 44 | 2.69 | 112.19 | <2E-16 | 0.03 | 0.36 | 27.49 | 3E-12 | 0.01 | 755.9 | 3E-12 | 5E-4 | 0.03 | 0.03 | 0.98 | 0.98 | -39.5 | -37.6 |
| **25** | 14 | 62 | 2.85 | 94.85 | <2E-16 | 0.04 | 0.39 | 23.43 | 2E-11 | 0.02 | 549 | 2E-11 | 1E-3 | 0.04 | 0.05 | 0.98 | 0.98 | -33.2 | -31.2 |
| **26** | 2 | 65 | - | - | - | - | - | - | - | - | - | - | - | - | - | - | - | - | - |
| **27** | 2 | 65 | - | - | - | - | - | - | - | - | - | - | - | - | - | - | - | - | - |
| **28** | 3 | 45 | 2.79 | 32.55 | 0.01 | 0.22 | 0.50 | 9.55 | 0.06 | 0.12 | 91.21 | 0.06 | 3E-5 | 2E-3 | 2E-3 | 0.99 | 0.97 | -6.25 | -8.96 |
| **29** | 4 | 63 | 3.05 | 20.10 | 2E-3 | 0.20 | 0.37 | 5.21 | 0.03 | 0.10 | 27.21 | 0.03 | 2E-3 | 0.03 | 0.03 | 0.93 | 0.89 | -1.99 | -3.83 |
| **30** | 6 | 57 | 2.94 | 39.27 | 2E-6 | 0.11 | 0.26 | 10.37 | 4E-4 | 0.03 | 107.5 | 4E-4 | 9E-4 | 0.02 | 0.02 | 0.96 | 0.95 | -9.99 | -10.6 |
| **31** | 5 | 63 | 3.41 | 43.20 | 2E-5 | 0.11 | 0.19 | 6.23 | 8E-3 | 0.04 | 38.83 | 8E-3 | 1E-3 | 0.01 | 0.02 | 0.93 | 0.90 | -7.49 | -8.66 |
| **32** | 8 | 56 | 3.31 | 27.78 | 1E-3 | 0.15 | 0.24 | 4.20 | 0.05 | 0.08 | 17.69 | 0.05 | 2E-3 | 0.02 | 0.02 | 0.89 | 0.85 | -3.32 | -5.16 |
| **33** | 6 | 62 | 2.92 | 32.63 | 5E-6 | 0.13 | 0.98 | 9.25 | 7E-4 | 0.16 | 85.68 | 7E-4 | 6E-3 | 0.12 | 0.13 | 0.95 | 0.94 | 0.01 | -0.61 |
| **34** | 9 | 60 | 3.24 | 65.46 | 2E-4 | 0.08 | 0.61 | 11.85 | 7E-3 | 0.07 | 140 | 7E-3 | 8E-5 | 5E-3 | 5E-3 | 0.98 | 0.98 | -8.72 | -10.6 |
| **35** | 4 | 66 | 3.35 | 135 | 5E-6 | 0.03 | 0.47 | 22.74 | 7E-4 | 0.02 | 517 | 1E-3 | 6E-6 | 1E-3 | 1E-3 | 0.99 | 0.99 | -13.9 | -15.7 |
| **36** | 2 | 42 | - | - | - | - | - | - | - | - | - | - | - | - | - | - | - | - | - |
| **37** | 4 | 39 | 1.35 | 6.04 | 0.02 | 0.30 | 0.44 | 8.66 | 0.01 | 0.07 | 75.15 | 0.01 | 3E-4 | 0.01 | 0.01 | 0.97 | 0.96 | -5.69 | -7.53 |
| **38** | 7 | 30 | 2.62 | 30.34 | 7E-7 | 0.1 | 0.38 | 7.81 | 5E-4 | 0.05 | 60.95 | 5E-4 | 0.01 | 0.14 | 0.15 | 0.92 | 0.91 | -1.42 | -1.59 |
| **39** | 7 | 35 | 2.68 | 36.01 | 3E-7 | 0.08 | 0.39 | 9.23 | 2E-4 | 0.05 | 85.2 | 2E-4 | 6E-3 | 0.1 | 0.11 | 0.94 | 0.93 | -3.5 | -3.66 |
| **40** | 7 | 38 | 1.36 | 4.20 | 8E-3 | 0.42 | 0.88 | 4.78 | 4E-3 | 0.28 | 22.92 | 4E-3 | 0.43 | 1.98 | 2.42 | 0.82 | 0.78 | 17.0 | 16.9 |

Note: * refers to sets that have been rescaled considering the intercept with a value of zero.

**Weibull model by simple linear regression.**

| **No.** | **Inter** | **t-value** | **p-value** | **ES**  **Boot** | **Pend** | **t-value** | **p-value** | **ES**  **Boot** | **Est F** | **p-value** | **SSE** | **SSR** | **SST** | **R^2^** | **R^2^_a_** | **AIC** | **BIC** |
| --- | --- | --- | --- | --- | --- | --- | --- | --- | --- | --- | --- | --- | --- | --- | --- | --- | --- |
| **1** | -1.11 | -5.93 | 5E-4 | 0.26 | 0.35 | 5.96 | 5E-4 | 0.08 | 35.59 | 5E-4 | 0.12 | 0.64 | 0.77 | 0.83 | 0.81 | 7.80 | 8.39 |
| **2** | -0.98 | -6.35 | 3E-4 | 0.21 | 0.29 | 5.95 | 5E-4 | 0.06 | 35.44 | 5E-4 | 0.08 | 0.44 | 0.52 | 0.83 | 0.81 | 4.39 | 4.98 |
| **3** | -1.69 | -32.76 | 1E-11 | 0.09 | 0.34 | 24.21 | 3E-10 | 0.02 | 586.2 | 3E-10 | 1E-3 | 0.06 | 0.06 | 0.98 | 0.98 | -22.6 | -21.1 |
| **4** | -4.49 | -14.22 | 7E-4 | 0.95 | 0.81 | 13.12 | 9E-4 | 0.19 | 172.2 | 9E-4 | 5E-4 | 0.03 | 0.03 | 0.98 | 0.97 | -4.77 | -5.94 |
| **5** | -4.02 | -7.31 | 5E-3 | 1.15 | 0.63 | 5.90 | 9E-3 | 0.23 | 34.87 | 9E-3 | 8E-3 | 0.10 | 0.11 | 0.92 | 0.89 | 0.75 | -0.41 |
| **6** | -1.61 | -19.94 | 5E-6 | 0.11 | 0.67 | 18.84 | 7E-6 | 0.04 | 354.9 | 7E-6 | 1E-3 | 0.10 | 0.11 | 0.98 | 0.98 | -3.55 | -3.71 |
| **7** | 0.92 | NA | NA | 7E-17 | 0.65 | NA | NA | 1E-16 | NAN | NA | 0 | 0 | 0 | 1 | NAN | -inf | -inf |
| **8** | 0.64 | NA | NA | 7E-17 | 1.98 | NA | NA | 0 | NAN | NA | 0 | 0 | 0 | 1 | NAN | -inf | -inf |
| **9** | 0.26 | NA | NA | 0 | 1.87 | NA | NA | 1E-16 | NAN | NA | 0 | 0 | 0 | 1 | NAN | -inf | -inf |
| **10** | -1.75 | -6.18 | 8E-3 | 0.44 | 0.88 | 6.33 | 7E-3 | 0.21 | 40.08 | 7E-3 | 0.02 | 0.35 | 0.38 | 0.93 | 0.91 | 6.96 | 5.79 |
| **11** | -2.17 | -12.67 | 1E-3 | 0.26 | 0.87 | 10.40 | 1E-3 | 0.12 | 108.3 | 1E-3 | 3E-3 | 0.12 | 0.13 | 0.97 | 0.96 | 1.89 | 0.72 |
| **12** | -1.32 | -9.98 | 2E-5 | 0.15 | 0.42 | 9.25 | 3E-5 | 0.04 | 85.72 | 3E-5 | 0.04 | 0.49 | 0.53 | 0.92 | 0.91 | 5.43 | 6.03 |
| **13** | -2.63 | -21.95 | 3E-9 | 0.15 | 0.45 | 17.04 | 3E-8 | 0.03 | 290.3 | 3E-8 | 4E-3 | 0.13 | 0.13 | 0.97 | 0.97 | -11.6 | -10.4 |
| **14** | -1.24 | -16.04 | 3E-6 | 0.09 | 0.37 | 17.41 | 2E-6 | 0.02 | 303.1 | 2E-6 | 9E-4 | 0.04 | 0.04 | 0.98 | 0.98 | -12.3 | -12.1 |
| **15** | -0.98 | -9.92 | 6E-5 | 0.14 | 0.34 | 12.68 | 1E-5 | 0.03 | 161 | 1-5 | 2E-3 | 0.07 | 0.08 | 0.96 | 0.96 | -8.42 | -8.18 |
| **16** | -0.90 | -15.78 | 4E-6 | 0.06 | 0.29 | 18.93 | 1E-6 | 0.01 | 358.3 | 1E-6 | 4E-4 | 0.02 | 0.02 | 0.98 | 0.98 | -17.0 | -16.8 |
| **17** | -0.62 | -8.72 | 1E-4 | 0.09 | 0.27 | 13.94 | 8E-6 | 0.02 | 194.5 | 8E-6 | 1E-3 | 0.04 | 0.04 | 0.97 | 0.96 | -13.6 | -13.4 |
| **18** | 0.26 | 0.40 | 1E-4 | 1.09 | 1.99 | 1.73 | 8E-6 | 2.37 | 3.01 | 0.18 | 6.21 | 6.24 | 12.46 | 0.50 | 0.33 | 21.3 | 20.1 |
| **18*** | 0 | - | - | - | 1.91 | 1.9 | 0.13 | 1.07 | 3.61 | 0.13 | 7.30 | 6.59 | 13.89 | 0.47 | 0.34 | 19.6 | 18.8 |
| **19** | 0.16 | 0.29 | 0.71 | 1.03 | 2.49 | 2.36 | 0.18 | 2.44 | 5.57 | 0.07 | 5.25 | 7.31 | 12.57 | 0.58 | 0.48 | 24.2 | 23.6 |
| **20** | -2.21 | -47.71 | 0.78 | 0.06 | 0.56 | 21.65 | 0.07 | 0.03 | 468.6 | 5E-11 | 3E-3 | 0.11 | 0.12 | 0.97 | 0.97 | -21.1 | -19.2 |
| **21** | -2.15 | -50.31 | 4E-15 | 0.07 | 0.41 | 17.29 | 5E-11 | 0.03 | 298.8 | 7E-10 | 4E-3 | 0.10 | 0.10 | 0.96 | 0.96 | -23.2 | -21.3 |
| **22** | -2.08 | -42.27 | 2E-15 | 0.06 | 0.47 | 17.16 | 7E-10 | 0.03 | 294.5 | 8E-10 | 5E-3 | 0.13 | 0.13 | 0.96 | 0.96 | -19.4 | -17.5 |
| **23** | -1.61 | -36.60 | 1E-14 | 0.07 | 0.44 | 18.22 | 8E-10 | 0.04 | 331.9 | 4E-10 | 3E-3 | 0.10 | 0.11 | 0.96 | 0.96 | -22.5 | -20.6 |
| **24** | -1.84 | -72.75 | 1E-13 | 0.02 | 0.42 | 30.11 | 4E-10 | 0.01 | 906.3 | 1E-12 | 4E-4 | 0.03 | 0.03 | 0.99 | 0.99 | -38.1 | -36.1 |
| **25** | -1.67 | -37.36 | <2E-16 | 0.06 | 0.48 | 19.33 | 1E-12 | 0.03 | 373.6 | 2E-10 | 3E-3 | 0.11 | 0.11 | 0.97 | 0.97 | -22.1 | -20.2 |
| **26** | -0.39 | -3.47 | 8E-14 | 0.13 | 0.24 | 6.25 | 2E-10 | 0.06 | 39.17 | 4E-4 | 0.02 | 0.15 | 0.17 | 0.85 | 0.82 | -5.16 | -4.57 |
| **27** | -0.53 | -6.36 | 0.01 | 0.11 | 0.33 | 11.51 | 4E-4 | 0.04 | 132.6 | 3E-4 | 9E-4 | 0.03 | 0.03 | 0.97 | 0.96 | -8.54 | -9.17 |
| **28** | -1.57 | -10.02 | 5E-4 | 0.21 | 0.51 | 9.45 | 6E-4 | 0.07 | 89.33 | 6E-4 | 4E-3 | 0.107 | 0.11 | 0.96 | 0.95 | -1.09 | -1.71 |
| **29** | -1.35 | -10.15 | 5E-4 | 0.19 | 0.40 | 8.90 | 8E-4 | 0.06 | 79.3 | 8E-4 | 3E-3 | 0.07 | 0.08 | 0.95 | 0.94 | -3.09 | -3.72 |
| **30** | -1.60 | -20.36 | 3E-5 | 0.10 | 0.34 | 12.67 | 2E-4 | 0.03 | 160.6 | 2E-4 | 1E-3 | 0.02 | 0.02 | 0.98 | 0.97 | -9.41 | -10.0 |
| **31** | -0.99 | -14.77 | 6E-6 | 0.10 | 0.24 | 12.40 | 1E-5 | 0.02 | 153.7 | 1E-5 | 1E-3 | 0.04 | 0.04 | 0.96 | 0.96 | -13.9 | -13.7 |
| **32** | -1.05 | -12.76 | 1E-5 | 0.12 | 0.26 | 10.86 | 3E-5 | 0.03 | 117.8 | 3E-5 | 2E-3 | 0.06 | 0.05 | 0.95 | 0.94 | -10.6 | -10.4 |
| **33** | -1.54 | -19.35 | 4E-5 | 0.12 | 1.17 | 12.41 | 2E-4 | 0.14 | 154.1 | 2E-4 | 2E-3 | 0.10 | 0.10 | 0.97 | 0.97 | -1.37 | -2.00 |
| **34** | -0.90 | -4.95 | 1E-3 | 0.21 | 0.57 | 6.65 | 2E-4 | 0.12 | 44.31 | 2E-4 | 0.09 | 0.61 | 0.71 | 0.86 | 0.84 | 7.36 | 7.96 |
| **35** | -1.28 | -7.37 | 3E-4 | 0.32 | 0.87 | 9.59 | 7E-5 | 0.16 | 90.31 | 7E-5 | 0.02 | 0.31 | 0.33 | 0.94 | 0.93 | 2.82 | 3.06 |
| **36** | -0.45 | -3.79 | 0.03 | 0.13 | 0.92 | 8.45 | 3E-3 | 0.13 | 71.49 | 3E-3 | 6E-3 | 0.14 | 0.15 | 0.96 | 0.95 | 2.57 | 1.40 |
| **36*** | 0 | - | - | - | 0.68 | 3.68 | 0.02 | 0.16 | 13.55 | 0.02 | 0.25 | 0.85 | 1.10 | 0.77 | 0.71 | 9.35 | 8.57 |
| **37** | -3.42 | -12.45 | 6E-3 | 0.37 | 0.52 | 8.25 | 0.01 | 0.09 | 68.09 | 0.01 | 5E-4 | 0.01 | 0.02 | 0.97 | 0.96 | -4.03 | -5.87 |
| **38** | -4.39 | -7.25 | 7E-4 | 0.80 | 1.37 | 3.95 | 0.01 | 0.50 | 15.67 | 0.01 | 2.22 | 6.96 | 9.18 | 0.76 | 0.71 | 25.8 | 25.7 |
| **39** | -4.06 | -6.45 | 1E-3 | 0.84 | 1.29 | 3.60 | 0.01 | 0.51 | 12.96 | 0.01 | 2.9 | 7.54 | 10.45 | 0.72 | 0.66 | 26.4 | 26.2 |
| **40** | -3.21 | -9.91 | 1E-4 | 0.42 | 0.94 | 5.11 | 3E-3 | 0.26 | 26.11 | 3E-3 | 0.38 | 1.98 | 2.36 | 0.84 | 0.81 | 17.1 | 16.9 |

Note: * refers to sets that have been rescaled considering the intercept with a value of zero.

**Hyperbolic Tangent Function Model by simple linear regression.**

| **No.** | **Inter** | **t-value** | **p-value** | **ES Boot** | **Pend** | **t-value** | **p-value** | **ES Boot** | **Est F** | **p-value** | **SSE** | **SSR** | **SST** | **R^2^** | **R^2^_a_** | **AIC** | **BIC** |
| --- | --- | --- | --- | --- | --- | --- | --- | --- | --- | --- | --- | --- | --- | --- | --- | --- | --- |
| **1** | 0.39 | 4.36 | 3E-3 | 0.11 | 0.06 | 4.71 | 2E-3 | 0.02 | 22.22 | 2E-3 | 0.01 | 0.19 | 0.20 | 0.76 | 0.73 | -3.15 | -2.55 |
| **2** | 0.41 | 5.39 | 1E-3 | 0.09 | 0.05 | 4.42 | 3E-3 | 0.05 | 19.56 | 3E-3 | 0.04 | 0.13 | 0.18 | 0.73 | 0.69 | -5.98 | -5.39 |
| **3** | 0.20 | 6.68 | 5E-5 | 0.04 | 0.04 | 11.71 | 3E-7 | 6E-3 | 137.1 | 3E-7 | 2E-3 | 0.03 | 0.03 | 0.93 | 0.92 | -31.2 | -29.7 |
| **4** | -0.20 | -3.77 | 0.03 | 0.09 | 0.06 | 16.30 | 5E-4 | 7E-3 | 265.8 | 5E-4 | 6E-5 | 5E-3 | 5E-3 | 0.99 | 0.98 | -13.9 | -15.0 |
| **5*** | 0 | - | - | - | 0.03 | 18.41 | 5E-5 | 1E-3 | 338.8 | 5E-5 | 3E-4 | 0.01 | 0.01 | 0.99 | 0.98 | -12.2 | -13.0 |
| **6*** | 0 | - | - | - | 0.22 | 41.02 | 1E-8 | 6E-3 | 1683 | 1E-8 | 5E-5 | 0.01 | 0.01 | 0.99 | 0.99 | -18.5 | -18.6 |
| **7** | -0.08 | NA | NA | 0 | 1.67 | NA | NA | 0 | NaN | NA | 0 | 0 | 0 | 1 | NA | -Inf | -Inf |
| **8** | -1.66 | NA | NA | 0 | 2.92 | NA | NA | 0 | NaN | NA | 0 | 0 | 0 | 1 | NA | -Inf | -Inf |
| **9** | -1.18 | NA | NA | 0 | 2.11 | NA | NA | 0 | NaN | NA | 0 | 0 | 0 | 1 | NA | -Inf | -Inf |
| **10*** | 0 | - | - | - | 0.29 | 15.76 | 9E-5 | 0.01 | 248.3 | 9E-5 | 1E-3 | 0.06 | 0.06 | 0.98 | 0.98 | -3.55 | -4.33 |
| **11*** | 0 | - | - | - | 0.21 | 15.98 | 8E-5 | 0.01 | 255.5 | 8E-5 | 5E-4 | 0.03 | 0.03 | 0.98 | 0.98 | 5.45 | -6.89 |
| **12** | 0.18 | 2.77 | 0.02 | 0.06 | 0.1 | 9.01 | 4E-5 | 0.01 | 81.26 | 4E-5 | 0.01 | 0.11 | 0.12 | 0.92 | 0.91 | -7.35 | -6.75 |
| **13** | 0.11 | 2.79 | 0.02 | 0.04 | 0.03 | 9.59 | 5E-6 | 4E-3 | 92.05 | 5E-6 | 3E-3 | 0.03 | 0.04 | 0.91 | 0.90 | -25.3 | -24.1 |
| **14** | 0.24 | 5.18 | 2E-3 | 0.08 | 0.07 | 12.11 | 1E-4 | 0.01 | 146.8 | 6E-5 | 6E-4 | 0.01 | 0.02 | 0.96 | 0.96 | -15.3 | -15.4 |
| **15** | 0.43 | 5.15 | 2E-3 | 0.10 | 0.07 | 6.84 | 4E-4 | 0.01 | 46.86 | 4E-4 | 0.01 | 0.10 | 0.11 | 0.88 | 0.87 | -5.99 | -5.75 |
| **16** | 0.41 | 9.14 | 9E-5 | 0.05 | 0.06 | 11.50 | 2E-5 | 8E-3 | 132.4 | 2E-5 | 1E-3 | 0.02 | 0.03 | 0.95 | 0.95 | -16.0 | -15.8 |
| **17** | 0.56 | 7.72 | 2E-4 | 0.09 | 0.06 | 7.09 | 3E-4 | 0.01 | 50.27 | 3E-4 | 9E-3 | 0.07 | 0.08 | 0.89 | 0.88 | -8.49 | -8.25 |
| **18*** | 0 | - | - | - | 1.37 | 5.32 | 5E-3 | 0.22 | 28.4 | 5E-3 | 0.20 | 1.43 | 1.64 | 0.87 | 0.84 | 12.0 | 11.2 |
| **19*** | 0 | - | - | - | 1.37 | 4.58 |  | 0.27 | 21.01 | 5E-3 | 0.92 | 2.73 | 3.65 | 0.80 | 0.77 | 16.3 | 15.9 |
| **20*** | 0 | - | - | - | 0.10 | 38.00 | 1E-14 | 4E-4 | 1444 | 1E-14 | 8E-5 | 9E-3 | 9E-3 | 0.99 | 0.99 | -58.5 | -57.2 |
| **21** | 0.03 | 4.54 | 6E-4 | 0.01 | 0.07 | 24.13 | 1E-11 | 4E-3 | 582.3 | 1E-11 | 3E-5 | 1E-3 | 1E-3 | 0.98 | 0.98 | -79.5 | -77.6 |
| **22*** | 0 | - | - | - | 0.10 | 43.23 | 1E-15 | 2E-3 | 1869 | 1E-15 | 4E-5 | 6E-3 | 6E-3 | 0.99 | 0.99 | -62.9 | -61.6 |
| **23** | 0.05 | 3.45 | 4E-3 | 0.01 | 0.13 | 23.04 | 2E-11 | 7E-3 | 530.9 | 2E-11 | 1E-4 | 6E-3 | 6E-3 | 0.97 | 0.98 | -62.1 | -60.2 |
| **24** | 0.06 | 5.62 | 1E-3 | 0.01 | 0.09 | 19.35 | 2E-10 | 8E-3 | 374.5 | 2E-10 | 1E-4 | 4E-3 | 4E-3 | 0.96 | 0.97 | -67.5 | -65.6 |
| **25*** | 0 | - | - | - | 0.15 | 70.18 | <2E-16 | 1E-3 | 4925 | <2E-16 | 1E-5 | 5E-3 | 5E-3 | 0.99 | 0.99 | 34.9 | -65.7 |
| **26** | 0.51 | 8.21 | 7E-5 | 0.08 | 0.09 | 8.50 | 6E-5 | 0.02 | 72.27 | 6E-5 | 8E-3 | 0.08 | 0.08 | 0.91 | 0.89 | -10.5 | -9.90 |
| **27** | 0.45 | 5.92 | 4E-3 | 0.09 | 0.13 | 8.71 | 9E-4 | 0.02 | 75.87 | 9E-4 | 1E-3 | 0.03 | 0.03 | 0.95 | 0.94 | -7.96 | -8.58 |
| **28*** | 0 | - | - | - | 0.14 | 13.56 | 3E-5 | 0.01 | 183.9 | 3E-5 | 2E-3 | 0.09 | 0.09 | 0.97 | 0.97 | -3.99 | -4.41 |
| **29** | 0.23 | 3.77 | 0.01 | 0.08 | 0.09 | 7.63 | 1E-3 | 0.02 | 58.31 | 1E-3 | 1E-3 | 0.02 | 0.02 | 0.93 | 0.92 | -10.7 | -11.3 |
| **30** | 0.18 | 4.96 | 7E-3 | 0.04 | 0.05 | 7.86 | 1E-3 | 0.01 | 61.88 | 1E-3 | 5E-4 | 7E-3 | 8E-3 | 0.93 | 0.92 | -16.7 | -17.4 |
| **31** | 0.35 | 8.96 | 1E-4 | 0.05 | 0.04 | 8.08 | 1E-4 | 7E-3 | 65.34 | 1E-4 | 1E-3 | 0.01 | 0.02 | 0.91 | 0.90 | -19.6 | -19.3 |
| **32** | 0.34 | 7.49 | 2E-4 | 0.06 | 0.04 | 7.36 | 3E-4 | 9E-3 | 54.24 | 3E-4 | 2E-3 | 0.02 | 0.02 | 0.90 | 0.88 | -17.3 | -17.0 |
| **33** | -0.27 | -7.69 | 1E-3 | 0.04 | 0.49 | 19.74 | 3E-5 | 0.03 | 389.9 | 3E-5 | 2E-5 | 2E-3 | 2E-3 | 0.99 | 0.99 | -22.9 | -23.5 |
| **34*** | 0 | - | - | - | 0.29 | 11.05 | 4E-6 | 0.04 | 122.1 | 4E-6 | 0.04 | 0.61 | 0.65 | 0.93 | 0.93 | 5.36 | 5.76 |
| **35*** | 0 | - | - | - | 0.45 | 8.72 | 5E-5 | 0.06 | 76.14 | 5E-5 | 0.10 | 1.10 | 1.20 | 0.91 | 0.90 | 10.9 | 11.0 |
| **36*** | 0 | - | - | - | 0.74 | 8.14 | 1E-3 | 0.10 | 66.39 | 1E-3 | 0.02 | 0.45 | 0.48 | 0.94 | 0.93 | 6.19 | 5.41 |
| **37*** | 0 | - | - | - | 0.03 | 42.94 | 2E-5 | 5E-4 | 1843 | 2E-5 | 9E-7 | 5E-4 | 5E-4 | 0.99 | 0.99 | -19.9 | -21.1 |
| **38*** | 0 | - | - | - | 0.07 | 11.64 | 2E-5 | 6E-3 | 135.5 | 2E-5 | 4E-4 | 0.01 | 0.01 | 0.95 | 0.95 | -21.7 | -21.8 |
| **39*** | 0 | - | - | - | 0.07 | 14.08 | 8E-6 | 4E-3 | 198.3 | 8E-6 | 2E-4 | 9E-3 | 9E-3 | 0.97 | 0.97 | -22.7 | -22.8 |
| **40*** | 0 | - | - | - | 0.08 | 13.91 | 8E-6 | 6E-3 | 193.6 | 8E-6 | 3E-3 | 0.01 | 0.01 | 0.97 | 0.97 | -20.9 | -21.1 |

Note: * refers to sets that have been rescaled considering the intercept with a value of zero.

**Polynomial equation of second degree by simple linear regression.**

| **No.** | **a** | **t-value** | **p-value** | **ES Boot** | **b** | **ES Boot** | **c** | **t-value** | **p-value** | **ES Boot** | **Est F** | **p-value** | **SSE** | **SSR** | **SST** | **R^2^** | **R^2^ _a_** | **AIC** | **BIC** |
| --- | --- | --- | --- | --- | --- | --- | --- | --- | --- | --- | --- | --- | --- | --- | --- | --- | --- | --- | --- |
| **1** | 42.08 | 5.57 | 1E-3 | 10.51 | 0.76 | 1.62 | -3E-3 | -1.49 | 0.18 | 0.05 | 4.82 | 0.05 | 880.05 | 1413.5 | 2293.6 | 0.61 | 0.48 | 79.1 | 79.8 |
| **2** | 42.81 | 6.77 | 5E-4 | 8.76 | 0.64 | 1.23 | -2E-3 | -1.53 | 0.17 | 0.04 | 4.94 | 0.05 | 601.9 | 992.2 | 1594.1 | 0.62 | 0.49 | 75.9 | 76.7 |
| **3** | 25.82 | 8.12 | 1E-5 | 4.47 | 0.51 | 0.22 | -1E-3 | -4.04 | 2E-3 | 2E-3 | 31.57 | 8E-5 | 52.88 | 370.86 | 423.74 | 0.87 | 0.84 | 83.2 | 85.2 |
| **4** | 8.28 | 1.46 | 0.28 | 27.98 | 0.31 | 0.36 | -3E-4 | -3.65 | 0.06 | -1E-3 | 66.38 | 0.01 | 0.44 | 29.60 | 30.05 | 0.98 | 0.97 | 31.1 | 29.5 |
| **4*** | 0 | - | - | - | 0.39 | 0.05 | -4E-4 | -8.41 | 3E-3 | 3E-3 | 363.8 | 2E-4 | 0.25 | 61.17 | 61.42 | 0.99 | 0.99 | 32.7 | 31.5 |
| **5** | 6.24 | 0.88 | 0.47 | 35.66 | 0.24 | 0.46 | -2E-4 | -2.45 | 0.13 | -1E-3 | 20.5 | 0.04 | 2.23 | 45.90 | 48.14 | 0.95 | 0.90 | 33.3 | 31.7 |
| **5*** | 0 | - | - | - | 0.30 | 0.05 | -3E-4 | -6.70 | 6E-3 | -3E-4 | 189.6 | 6E-4 | 0.50 | 63.81 | 64.31 | 0.99 | 0.98 | 32.9 | 31.8 |
| **6** | 18.21 | 3.12 | 0.03 | 7.82 | 4.44 | 3.15 | -0.06 | -3.57 | 0.02 | 0.25 | 29.2 | 4E-3 | 24.45 | 357.08 | 381.54 | 0.93 | 0.90 | 55.4 | 55.2 |
| **7** | 64 | NA | NA | 5E-14 | 36 | 6E-14 | -8 | NA | NA | 1E-14 | NA | NA | 0 | 0 | 0 | 1 | NA | -INF | -INF |
| **8** | -26.3 | NA | NA | 1E-13 | 145.9 | 2E-13 | -34.6 | NA | NA | 5E-14 | NA | NA | 0 | 0 | 0 | 1 | NA | -INF | -INF |
| **9** | -27.5 | NA | NA | 8E-13 | 129.5 | 1E-13 | -29.0 | NA | NA | 5E-14 | NA | NA | 0 | 0 | 0 | 1 | NA | -INF | -INF |
| **10** | 10.76 | 0.93 | 0.44 | 16.68 | 9.82 | 5.25 | -0.27 | -2.71 | 0.11 | 0.35 | 12.83 | 0.07 | 21.79 | 279.68 | 301.48 | 0.92 | 0.85 | 42.3 | 40.7 |
| **10*** | 0 | - | - | - | 11.91 | 2.37 | -0.34 | -5.04 | 0.01 | 0.28 | 77.06 | 2E-3 | 7.82 | 401.6 | 409.4 | 0.98 | 0.96 | 42.1 | 40.9 |
| **11** | 4.25 | 0.64 | 0.58 | 10.27 | 7.83 | 3.13 | -0.19 | -3.34 | 0.07 | 0.24 | 34.28 | 0.02 | 2.69 | 92.57 | 95.26 | 0.97 | 0.94 | 36.8 | 35.2 |
| **11*** | 0 | - | - | - | 8.65 | 1.21 | -0.22 | -6.19 | 8E-3 | 0.14 | 184.5 | 7E-4 | 0.91 | 111.67 | 112.58 | 0.99 | 0.98 | 35.7 | 34.5 |
| **12** | 31.71 | 5.22 | 1E-3 | 7.06 | 0.88 | 1.09 | -3E-3 | -1.75 | 0.12 | 0.02 | 18.71 | 1E-3 | 233.93 | 1246.68 | 1489.62 | 0.84 | 0.79 | 84.6 | 85.8 |
| **13** | 16.60 | 5.74 | 3E-4 | 3.64 | 0.35 | 0.07 | -6E-4 | -5.34 | 6E-4 | 3E-4 | 60.23 | 1E-5 | 12.96 | 182.50 | 194.56 | 0.93 | 0.92 | 70.1 | 71.7 |
| **14** | 40.18 | 8.73 | 3E-4 | 6.14 | 0.64 | 0.42 | -1E-3 | -3.59 | 0.01 | 6E-3 | 20.84 | 3E-3 | 36.26 | 302.33 | 338.60 | 0.89 | 0.85 | 59.8 | 60.1 |
| **15** | 47.93 | 8.49 | 3E-4 | 7.50 | 0.59 | 0.53 | -1E-3 | -2.78 | 0.03 | 7E-3 | 11.29 | 0.01 | 100.70 | 455.07 | 555.77 | 0.81 | 0.74 | 63.0 | 63.3 |
| **16** | 48.07 | 11.86 | 7E-5 | 5.68 | 0.48 | 0.44 | -1E-3 | -2.89 | 0.03 | 8E-3 | 17.82 | 5E-3 | 32.85 | 234.25 | 267.10 | 0.87 | 0.82 | 57.7 | 58.0 |
| **17** | 56.58 | 12.23 | 6E-5 | 6.63 | 0.45 | 0.45 | -1E-3 | -2.57 | 0.05 | 6E-3 | 10.41 | 0.01 | 73.29 | 305.28 | 378.58 | 0.80 | 0.72 | 59.8 | 60.2 |
| **18** | -35.5 | -0.65 | 0.55 | 149.21 | 165.33 | 388.24 | -41.4 | -1.59 | 0.20 | 251.92 | 3.01 | 0.19 | 1096.56 | 2199.37 | 3295.93 | 0.66 | 0.44 | 60.5 | 59.6 |
| **18*** | 0 | - | - | - | 107.93 | 46.82 | -25.58 | -2.99 | 0.04 | 63.51 | 30.11 | 3E-3 | 166.99 | 2513.49 | 2680.48 | 0.93 | 0.90 | 59.3 | 58.6 |
| **19** | -66.4 | -1.56 | 0.19 | 77.91 | 204.89 | 167.02 | -51.5 | -2.50 | 0.06 | 85.03 | 7.58 | 0.04 | 484.15 | 1835.69 | 2319.84 | 0.79 | 0.68 | 66.9 | 66.6 |
| **19*** | 0 | - | - | - | 95.55 | 51.95 | -21.09 | -2.77 | 0.03 | 66.25 | 31.9 | 1E-3 | 232.22 | 2962.11 | 3194.34 | 0.92 | 0.89 | 68.2 | 68.0 |
| **20** | 6.74 | 8.90 | 2E-6 | 1.04 | 3.83 | 0.50 | -0.09 | -13.55 | 3E-8 | 0.04 | 427 | 3E-11 | 0.25 | 19.49 | 19.74 | 0.98 | 0.98 | 52.4 | 54.9 |
| **21** | 8.44 | 49.12 | 3E-14 | 0.23 | 2.42 | 0.11 | -0.05 | -31.91 | 3E-12 | 0.01 | 4422 | <2E-16 | 1E-3 | 1.00 | 1.00 | 0.99 | 0.99 | 10.9 | 13.4 |
| **22** | 8.37 | 12.16 | 1E-7 | 0.78 | 3.17 | 0.36 | -0.07 | -10.78 | 3E-7 | 0.03 | 448.2 | 2E-11 | 0.19 | 16.12 | 16.32 | 0.98 | 0.98 | 49.7 | 52.3 |
| **23** | 14.26 | 30.47 | 5E-12 | 0.62 | 3.98 | 0.25 | -0.08 | -19.86 | 5E-10 | 0.02 | 1540 | 3E-14 | 0.02 | 7.44 | 7.47 | 0.99 | 0.99 | 38.9 | 41.5 |
| **24** | 12.20 | 14.85 | 1E-8 | 1.35 | 3.14 | 0.45 | -0.07 | -9.62 | 1E-6 | 0.03 | 275.4 | 4E-10 | 0.45 | 22.92 | 23.38 | 0.98 | 0.97 | 54.6 | 57.2 |
| **25** | 13.65 | 23.61 | 8E-11 | 0.92 | 3.98 | 0.26 | -0.08 | -14.67 | 1E-8 | 0.01 | 1158 | 1E-13 | 0.05 | 11.37 | 11.42 | 0.99 | 0.99 | 44.8 | 47.4 |
| **26** | 63.55 | 27.52 | 1E-7 | 4.52 | 0.35 | 0.79 | -9E-4 | -0.89 | 0.40 | 0.03 | 21.88 | 1E-3 | 14.59 | 106.46 | 121.06 | 0.87 | 0.83 | 55.8 | 56.6 |
| **27** | 56.26 | 9.35 | 2E-3 | 9.74 | 1.21 | 1.58 | -0.01 | -1.47 | 0.23 | 0.04 | 8.21 | 0.06 | 32.91 | 180.08 | 212.98 | 0.84 | 0.74 | 45.4 | 44.6 |
| **28** | 26.55 | 3.91 | 0.03 | 9.76 | 2.13 | 1.38 | -0.02 | -2.51 | 0.08 | 0.05 | 15.68 | 0.02 | 21.96 | 229.60 | 251.67 | 0.91 | 0.85 | 46.9 | 46.1 |
| **29** | 32.19 | 4.91 | 0.01 | 10.25 | 1.48 | 1.73 | -0.01 | -1.66 | 0.19 | 0.05 | 10.1 | 0.04 | 31.78 | 213.99 | 245.77 | 0.87 | 0.78 | 46.5 | 45.6 |
| **30** | 24.14 | 6.08 | 8E-3 | 5.61 | 1.05 | 0.84 | -8E-3 | -1.86 | 0.15 | 0.02 | 15.75 | 0.02 | 7.47 | 78.39 | 85.87 | 0.91 | 0.85 | 40.4 | 39.6 |
| **31** | 38.72 | 11.84 | 7E-5 | 4.85 | 0.65 | 0.54 | -3E-3 | -2.58 | 0.04 | 0.02 | 20.26 | 4E-3 | 15.96 | 129.28 | 145.25 | 0.89 | 0.84 | 53.0 | 53.3 |
| **32** | 37.66 | 10.03 | 1E-4 | 5.50 | 0.70 | 0.52 | -3E-3 | -2.47 | 0.05 | 0.01 | 17.17 | 5E-3 | 24.84 | 170.64 | 195.48 | 0.87 | 0.82 | 55.2 | 55.5 |
| **33** | -6.00 | -1.35 | 0.26 | 9.20 | 30.31 | 9.92 | -3.41 | -3.33 | 0.04 | 2.78 | 109.1 | 1E-3 | 0.37 | 27.26 | 27.64 | 0.98 | 0.97 | 34.1 | 33.3 |
| **33*** | 0 | - | - | - | 17.25 | 1.46 | 0 | - | - | - | 251 | 2E-5 | 3.82 | 196.36 | 192.54 | 0.98 | 0.98 | 41.8 | 41.4 |
| **34** | 40.12 | 4.75 | 3E-3 | 14.76 | 4.40 | 4.76 | -0.07 | -2.57 | 0.04 | 0.25 | 8.23 | 0.01 | 452.19 | 1242.04 | 1694.23 | 0.73 | 0.64 | 77.9 | 78.7 |
| **35** | 22.44 | 10.71 | 1E-4 | 3.76 | 8.65 | 1.23 | -0.24 | -6.85 | 1E-3 | 0.11 | 438.8 | 2E-6 | 0.13 | 24.33 | 24.47 | 0.99 | 0.99 | 39.6 | 39.9 |
| **36** | 22.86 | 2.58 | 0.08 | 15.41 | 23.58 | 11.45 | -1.77 | -2.62 | 0.07 | 1.95 | 25.16 | 0.01 | 14.29 | 239.65 | 253.94 | 0.94 | 0.90 | 47.2 | 46.3 |
| **36*** | 0 | - | - | - | 36.09 | 7.74 | -3.05 | -4.23 | 0.01 | 2.47 | 88.19 | 4E-4 | 17.58 | 774.68 | 792.26 | 0.97 | 0.96 | 52.2 | 51.6 |
| **37** | 10.99 | 4.03 | 0.15 | 3.18 | 0.22 | 0.09 | -3E-4 | -0.91 | 0.53 | 5E-4 | 69.12 | 0.08 | 0.02 | 2.44 | 2.46 | 0.99 | 0.97 | 17.4 | 14.9 |
| **37*** | 0 | - | - | - | 0.51 | 0.14 | -1E-3 | -2.74 | -1E-3 | 2E-3 | 78.51 | 0.01 | 0.54 | 42.33 | 42.87 | 0.98 | 0.97 | 26.8 | 24.9 |
| **38** | 0.73 | 0.32 | 0.75 | 4.19 | 3.65 | 3.35 | -0.10 | -3.86 | 0.01 | 0.52 | 34.2 | 3E-3 | 2.79 | 47.82 | 50.61 | 0.94 | 0.91 | 41.3 | 41.1 |
| **38*** | 0 | - | - | - | 3.80 | 0.98 | -0.10 | -5.84 | 2E-3 | 0.47 | 117.2 | 6E-5 | 1.04 | 49.10 | 50.15 | 0.97 | 0.97 | 39.5 | 39.3 |
| **39** | 2.15 | 0.75 | 0.49 | 4.23 | 3.56 | 2.89 | -0.09 | -2.76 | 0.05 | 0.43 | 24.27 | 5E-3 | 6.38 | 77.48 | 83.86 | 0.92 | 0.88 | 44.7 | 44.5 |
| **39*** | 0 | - | - | - | 1.79 | 0.86 | 0 | - | - | - | 34.1 | 1E-3 | 76.28 | 434.31 | 510.59 | 0.85 | 0.82 | 52.8 | 52.7 |
| **40** | 3.02 | 0.95 | 0.39 | 5.00 | 3.95 | 3.84 | -0.10 | -2.82 | 0.04 | 0.96 | 22.74 | 6E-3 | 8.41 | 95.54 | 103.95 | 0.91 | 0.87 | 46.2 | 45.9 |
| **40*** | 0 | - | - | - | 1.97 | 0.98 | 0 | - | - | - | 30.23 | 1E-3 | 118.53 | 595.55 | 714.09 | 0.83 | 0.81 | 55.0 | 54.9 |

Note: * refers to sets that have been rescaled considering the intercept with a value of zero.

**Polynomial equation of third degree by simple linear regression.**

| **No** | **a** | **t-value** | **p-value** | **ES Boot** | **b** | **ES Boot** | **c** | **ES Boot** | **d** | **t-value** | **p-value** | **ES Boot** | **Est F** | **p-value** | **SSE** | **SSR** | **SST** | **R^2^** | **R^2^ _a_** | **AIC** | **BIC** |
| --- | --- | --- | --- | --- | --- | --- | --- | --- | --- | --- | --- | --- | --- | --- | --- | --- | --- | --- | --- | --- | --- |
| **1** | 34.34 | 4.34 | 7E-3 | 12.87 | 2.03 | 3.83 | -0.02 | 0.28 | -8E-5 | 1.73 | 0.14 | 6E-3 | 5.29 | 0.05 | 227.61 | 882.02 | 1159.64 | 0.76 | 0.61 | 76.8 | 77.8 |
| **2** | 36.06 | 5.58 | 2E-3 | 10.45 | 1.76 | 3.53 | -0.02 | 0.37 | 7E-5 | 1.85 | 0.12 | 0.01 | 5.78 | 0.04 | 169.26 | 587.73 | 756.99 | 0.77 | 0.64 | 73.2 | 74.1 |
| **3** | 21.70 | 7.42 | 7E-5 | 4.63 | 0.94 | 0.48 | -7E-3 | 0.01 | -1E-5 | 2.63 | 0.03 | 1E-4 | 37.22 | 4E-5 | 14.24 | 212.94 | 198.70 | 0.93 | 0.90 | 77.7 | 80.2 |
| **4** | 7.31 | 0.48 | 0.71 | 69.5 | 0.33 | 1.31 | -4E-5 | 8E-3 | 1E-7 | 0.076 | 0.95 | 1E-5 | 22.26 | 0.15 | 0.44 | 29.43 | 29.87 | 0.98 | 0.94 | 33.1 | 31.1 |
| **4*** | 0 | - | - | - | 0.47 | 0.24 | -1E-3 | 3E-3 | 1E-6 | 1.17 | 0.36 | 1E-5 | 273.0 | 3E-3 | 0.08 | 36.28 | 36.37 | 0.99 | 0.99 | 32.1 | 30.5 |
| **5** | 12.85 | 0.74 | 0.59 | 84.3 | 0.09 | 1.61 | 5E-4 | 0.01 | -1E-6 | -0.45 | 0.73 | 2E-5 | 8.29 | 0.24 | 1.53 | 38.14 | 39.68 | 0.96 | 0.84 | 34.3 | 32.4 |
| **5*** | 0 | - | - | - | 0.33 | 0.30 | -6E-4 | 4E-3 | 4E-7 | 0.39 | 0.73 | 1E-5 | 90.72 | 0.01 | 0.43 | 59.31 | 59.74 | 0.99 | 0.98 | 34.6 | 33.0 |
| **6** | 11.13 | 2.25 | 0.10 | 6.89 | 8.01 | 4.64 | -0.29 | 0.72 | 3E-3 | 2.387 | 0.09 | 0.02 | 44.24 | 5E-3 | 2.78 | 123.13 | 125.91 | 0.97 | 0.95 | 49.9 | 49.7 |
| **6*** | 0 | - | - | - | 4.74 | 2.43 | 0 | - | 0 | - | - | - | 18.11 | 0.01 | 840.6 | 3806.63 | 4646.97 | 0.81 | 0.77 | 51.4 | 50.6 |
| **7** | 64 | NA | NA | 5E-14 | 36 | 6E-14 | -8 | 1E-14 | NA | NA | NA | - | NAN | NA | 0 | 0 | 0 | 1 | NA | -INF | -INF |
| **8** | -26.3 | NA | NA | 1E-13 | 145.9 | 2E-13 | -34.6 | 5E-14 | NA | NA | NA | - | NAN | NA | 0 | 0 | 0 | 1 | NA | -INF | -INF |
| **9** | -27.5 | NA | NA | 7E-14 | 129.5 | 1E-13 | -29.0 | 4E-14 | NA | NA | NA | - | NAN | NA | 0 | 0 | 0 | 1 | NA | -INF | -INF |
| **10** | -7.62 | -17.34 | 0.03 | 0.58 | 21.30 | 0.35 | -1.61 | 0.06 | 0.03 | 55.87 | 0.01 | 2E-3 | 1439.0 | 6E-3 | 0 | 0.08 | 0.08 | 1 | 1 | 4.08 | 2.13 |
| **11** | -4.30 | -0.52 | 0.69 | 11.31 | 13.17 | 6.65 | -0.81 | 1.12 | 0.01 | 1.37 | 0.39 | 0.05 | 33.79 | 0.12 | 0.31 | 31.90 | 32.22 | 0.99 | 0.96 | 33.5 | 31.5 |
| **11*** | 0 | - | - | - | 11.30 | 2.09 | -0.63 | 0.68 | 0.01 | 1.87 | 0.20 | 0.06 | 227.3 | 4E-3 | 0.11 | 40.49 | 40.61 | 0.99 | 0.99 | 32.6 | 31.1 |
| **12** | 27.45 | 4.60 | 3E-3 | 10.26 | 1.78 | 4.89 | -0.01 | 0.78 | 6E-5 | 1.68 | 0.14 | 0.04 | 16.71 | 2E-3 | 101.23 | 845.79 | 947.03 | 0.89 | 0.84 | 82.8 | 84.3 |
| **13** | 11.79 | 6.49 | 3E-4 | 2.42 | 0.58 | 0.09 | -2E-3 | 1E-3 | 3E-6 | 4.74 | 0.02 | 3E-6 | 155.4 | 9E-7 | 0.65 | 43.34 | 44.00 | 0.98 | 0.97 | 56.3 | 58.3 |
| **14** | 34.07 | 9.31 | 7E-4 | 6.10 | 1.24 | 0.74 | -0.01 | 0.03 | 2E-5 | 2.84 | 0.04 | 6E-3 | 36.3 | 2E-3 | 3.66 | 99.99 | 103.65 | 0.96 | 0.93 | 52.9 | 53.3 |
| **15** | 40.85 | 8.25 | 1E-3 | 8.22 | 1.30 | 1.03 | -0.01 | 0.03 | 2E-5 | 2.43 | 0.07 | 4E-4 | 16.96 | 9E-3 | 14.38 | 182.98 | 197.37 | 0.92 | 0.87 | 57.7 | 58.1 |
| **16** | 43.51 | 10.80 | 4E-4 | 7.62 | 0.93 | 1.04 | -7E-3 | 0.03 | 1E-5 | 1.93 | 0.12 | 3E-4 | 19.59 | 7E-3 | 8.25 | 1221.30 | 129.55 | 0.93 | 0.88 | 54.5 | 54.9 |
| **17** | 51.58 | 10.82 | 4E-4 | 7.51 | 0.95 | 1.01 | -8E-3 | 0.03 | 1E-5 | 1.78 | 0.14 | 3E-4 | 11.04 | 0.02 | 20.53 | 169.96 | 190.50 | 0.89 | 0.80 | 57.2 | 57.5 |
| **18** | -147.7 | -1.82 | 0.21 | 424.07 | 447.14 | 1544 | -231.5 | 1812 | 36.70 | 1.64 | 0.24 | 699 | 4.03 | 0.20 | 155.13 | 938.13 | 1093.26 | 0.85 | 0.64 | 57.3 | 56.3 |
| **18*** | 0 | - | - | - | 114.10 | 298.67 | -34.18 | 793.2 | 2.25 | 0.14 | 0.89 | 520.4 | 15.16 | 0.02 | 164.75 | 2496.94 | 2661.70 | 0.93 | 0.87 | 61.2 | 60.4 |
| **19** | -197.6 | -8.06 | 3E-3 | 141.3 | 531.26 | 561.97 | -271.2 | 762.2 | 42.42 | 6.30 | 8E-3 | 351.3 | 67.31 | 2E-3 | 1.91 | 128.78 | 130.69 | 0.98 | 0.97 | 50.3 | 50.0 |
| **20** | 4.71 | 7.04 | 3E-5 | 0.81 | 5.24 | 0.65 | -0.28 | 0.12 | 5E-3 | 4.27 | 1E-3 | 6E-3 | 737.1 | 4E-12 | 0.03 | 6.90 | 6.93 | 0.99 | 0.99 | 39.8 | 43.0 |
| **21** | 8.80 | 44.40 | 8E-13 | 0.28 | 2.17 | 0.20 | -0.02 | 0.04 | -9E-4 | 3E-4 | 0.02 | 2E-3 | 4456 | 6E-16 | 4E-4 | 0.60 | 0.60 | 0.99 | 0.99 | 5.74 | 8.94 |
| **22** | 9.39 | 10.23 | 1E-6 | 1.57 | 2.46 | 1.06 | 0.01 | 0.21 | -2E-3 | -1.56 | 0.14 | 0.01 | 338 | 2E-10 | 0.12 | 12.96 | 13.08 | 0.99 | 0.98 | 48.7 | 51.8 |
| **23** | 15.02 | 24.69 | 2E-10 | 1.04 | 3.46 | 0.68 | -0.02 | 0.13 | -1E-3 | -1.76 | 0.10 | 8E-3 | 1224 | 4E-13 | 0.01 | 5.68 | 5.70 | 0.97 | 0.99 | 37.1 | 40.3 |
| **24** | 10.81 | 10.27 | 1E-6 | 2.22 | 4.11 | 1.38 | -0.20 | 0.27 | 3E-3 | 1.85 | 0.09 | 0.01 | 225.7 | 1E-9 | 0.25 | 17.04 | 17.29 | 0.98 | 0.98 | 52.5 | 55.7 |
| **25** | 13.81 | 16.12 | 1E-8 | 1.66 | 3.87 | 1.02 | -0.06 | 0.20 | -4E-4 | -0.27 | 0.79 | 0.01 | 707 | 6E-12 | 0.05 | 11.28 | 11.34 | 0.99 | 0.99 | 46.7 | 49.9 |
| **26** | 58.17 | 22.96 | 2E-6 | 5.72 | 1.30 | 1.87 | -0.02 | 0.21 | 1E-4 | 2.74 | 0.04 | 8E-3 | 32.9 | 1E-3 | 2.15 | 42.56 | 44.72 | 0.95 | 0.92 | 49.5 | 50.5 |
| **27** | 47.88 | 8.38 | 0.01 | 8.16 | 3.09 | 1.84 | -0.07 | 0.13 | 6E-4 | 2.11 | 0.16 | 2E-3 | 13.23 | 0.07 | 2.81 | 55.89 | 58.71 | 0.95 | 0.88 | 40.4 | 39.4 |
| **28** | 15.39 | 6.65 | 0.02 | 7.84 | 4.64 | 2.32 | -0.11 | 0.22 | 8E-4 | 6.93 | 0.02 | 4E-9 | 190.7 | 5E-3 | 0.03 | 9.16 | 9.19 | 0.99 | 0.99 | 29.6 | 28.5 |
| **29** | 23.23 | 3.59 | 0.06 | 8.87 | 3.49 | 2.28 | -0.08 | 0.17 | 6E-4 | 1.99 | 0.18 | 3E-3 | 14.71 | 0.06 | 3.25 | 71.78 | 75.04 | 0.95 | 0.89 | 41.9 | 40.9 |
| **29*** | 0 | - | - | - | 6.81 | 3.04 | -0.18 | 0.44 | 1E-3 | 2.80 | 0.06 | 0.01 | 35.25 | 7E-3 | 15.16 | 534.26 | 549.43 | 0.97 | 0.94 | 52.0 | 51.1 |
| **30** | 17.78 | 9.01 | 0.01 | 3.15 | 2.48 | 0.89 | -0.06 | 0.07 | 4E-4 | 4.63 | 0.04 | 1E-3 | 89.37 | 0.01 | 0.04 | 6.67 | 6.72 | 0.99 | 0.98 | 27.7 | 26.6 |
| **31** | 35.55 | 10.42 | 4E-4 | 6.05 | 1.20 | 1.41 | -0.01 | 0.08 | 6E-5 | 1.65 | 0.17 | 1E-3 | 19.08 | 7E-3 | 5.36 | 76.85 | 82.22 | 0.93 | 0.88 | 50.8 | 51.2 |
| **32** | 33.83 | 8.97 | 8E-4 | 7.12 | 1.36 | 1.51 | -0.01 | 0.08 | 8E-5 | 1.80 | 0.14 | 1E-3 | 17.73 | 8E-3 | 7.06 | 93.90 | 100.96 | 0.93 | 0.87 | 52.8 | 93.9 |
| **33** | -13.30 | -1.86 | 0.20 | 20.22 | 45.98 | 39.88 | -11.9 | 25.51 | 1.27 | 1.24 | 0.33 | 5.25 | 86.66 | 0.01 | 0.12 | 15.35 | 15.46 | 0.99 | 0.98 | 32.7 | 31.6 |
| **33*** | 0 | - | - | - | 22.45 | 9.03 | -0.62 | 10.0 | -0.29 | -0.37 | 0.73 | 2.72 | 233.6 | 4E-4 | 0.18 | 42.02 | 42.20 | 0.99 | 0.99 | 36.7 | 35.9 |
| **34** | 19.30 | 2.70 | 0.04 | 16.17 | 12.25 | 8.83 | -0.55 | 1.77 | 6E-3 | 3.84 | 0.01 | 0.14 | 23.04 | 2E-3 | 22.72 | 313.9 | 336.6 | 0.93 | 0.89 | 67.5 | 68.5 |
| **35** | 19.52 | 6.70 | 2E-3 | 13.10 | 10.72 | 5.06 | -0.54 | 0.72 | 0.01 | 1.34 | 0.24 | 0.03 | 340.9 | 2E-5 | 0.06 | 16.74 | 16.80 | 0.99 | 0.99 | 38.6 | 39.0 |
| **36** | 10.56 | 0.85 | 0.48 | 22.79 | 41.82 | 27.41 | -7.17 | 11.07 | 0.42 | 1.30 | 0.32 | 1.38 | 21.21 | 0.04 | 4.08 | 129.81 | 133.89 | 0.97 | 0.92 | 45.5 | 44.4 |
| **36*** | 0 | - | - | - | 53.38 | 9.13 | 10.13 | 9.44 | 0.63 | 3.18 | 0.05 | 2.49 | 196.1 | 6E-4 | 0.90 | 177.23 | 178.14 | 0.99 | 0.99 | 45.3 | 44.5 |
| **37** | 16.25 | NA | NA | 2E-14 | -0.02 | 7E-16 | 2.69 | 8E-18 | -1E-5 | NA | NA | 2E-10 | NAN | NA | 0 | 0 | 0 | 1 | NA | -INF | -INF |
| **38** | -2.08 | -0.75 | 0.50 | 6.59 | 6.02 | 11.23 | -0.39 | 6.57 | 8E-3 | 1.45 | 0.24 | 1.10 | 29.84 | 9E-3 | 0.93 | 28.06 | 29.00 | 0.97 | 0.93 | 39.6 | 39.3 |
| **38*** | 0 | - | - | - | 4.93 | 3.83 | -0.28 | 3.25 | 5E-3 | 1.37 | 0.24 | 0.67 | 92.75 | 3E-5 | 36.20 | 2513.4 | 2549.60 | 0.98 | 0.97 | 38.8 | 38.6 |
| **39** | -2.65 | -1.12 | 0.34 | 4.10 | 7.61 | 5.39 | -0.60 | 2.83 | 0.01 | 2.92 | 0.06 | 0.47 | 49.48 | 4E-3 | 0.40 | 20.16 | 20.56 | 0.98 | 0.96 | 37.3 | 37.0 |
| **39*** | 0 | - | - | - | 1.79 | 0.86 | 0 | - | 0 | - | - | - |  |  |  |  |  |  |  |  |  |
| **40** | -2.42 | -0.98 | 0.39 | 6.28 | 8.54 | 10.22 | -0.68 | 5.63 | 0.01 | 3.169 | 0.05 | 0.87 | 52.77 | 4E-3 | 0.41 | 21.97 | 22.39 | 0.98 | 0.96 | 37.9 | 37.6 |
| **40*** | 0 | - | - | - | 1.97 | 0.98 | 0 | - | 0 | - | - | - | 30.23 | 1E-3 | 118.53 | 595.55 | 714.09 | 0.83 | 0.81 | 55.0 | 54.9 |

Note: * refers to sets that have been rescaled considering the intercept with a value of zero.

**Polynomial equation of fourth degree by simple linear regression.**

| **No.** | **a** | **t-value** | **p-value** | **ES Boot** | **b** | **ES Boot** | **c** | **ES Boot** | **d** | **ES Boot** | **e** | **t-value** | **p-value** | **ES Boot** | **Est F** | **p-value** | **SSE** | **SST** | **SSR** | **R^2^** | **R^2^ _a_** | **AIC** | **BIC** |
| --- | --- | --- | --- | --- | --- | --- | --- | --- | --- | --- | --- | --- | --- | --- | --- | --- | --- | --- | --- | --- | --- | --- | --- |
| **1** | 26.34 | 3.60 | 0.02 | 12.83 | 3.92 | 5.73 | -0.08 | 0.69 | 7E-4 | 0.02 | -1E-6 | -2.04 | 0.11 | 3E-4 | 7.54 | 0.03 | 57.10 | 488.09 | 430.98 | 0.88 | 0.76 | 72.4 | 73.5 |
| **2** | 29.3 | 5.05 | 7E-3 | 12.22 | 3.34 | 7.30 | -0.07 | 1.25 | 6E-4 | 0.07 | -1E-6 | -2.15 | 0.09 | 1E-3 | 8.66 | 0.02 | 31.39 | 271.95 | 303.34 | 0.89 | 0.79 | 68.2 | 69.4 |
| **3** | 18.22 | 7.31 | 1E-4 | 4.90 | 1.68 | 0.08 | -0.02 | 0.04 | 2E-4 | 7E-4 | -4E-7 | -2.78 | 0.02 | 4E-6 | 53.29 | 2E-5 | 3.10 | 97.60 | 94.50 | 0.96 | 0.95 | 70.8 | 73.7 |
| **4** | 94.14 | NA | NA | 1E-11 | -2.68 | 4E-13 | 0.03 | 4E-15 | -1E-4 | 1E-17 | 1E-7 | NA | NA | 1E-20 | NAN | NA | 0 | 0 | 0 | 1 | NA | -INF | -INF |
| **5** | 111.7 | NA | NA | 1E-11 | -3.35 | 5E-13 | 0.03 | 5E-15 | -1E-4 | 1E-17 | 1E-7 | NA | NA | 2E-20 | NAN | NA | 0 | 0 | 0 | 1 | NA | -INF | -INF |
| **6** | 4.60 | 1.61 | 0.24 | 3.87 | 14.13 | 3.26 | -1.21 | 0.75 | 0.04 | 0.05 | 4E-4 | -3.61 | 0.06 | 1E-3 | 169.2 | 5E-38 | 0.04 | 16.46 | 16.46 | 0.99 | 0.99 | 37.8 | 37.5 |
| **6*** | 0 | - | - | - | 4.74 | 2.43 | 0 | - | 0 | - | 0 | - | - | - | 18.11 | 0.01 | 840.6 | 3806.6 | 4646.9 | 0.81 | 0.77 | 51.4 | 50.6 |
| **7** | 64 | NA | NA | 5E-14 | 36 | 6E-14 | -8 | 2E-14 | NA | - | NA | NA | NA | - | NAN | NA | 0 | 0 | 0 | 1 | NA | -INF | -INF |
| **8** | -26.3 | NA | NA | 1E-3 | 145.9 | 1E-13 | -34.6 | 5E-14 | NA | - | NA | NA | NA | - | NAN | NA | 0 | 0 | 0 | 1 | NA | -INF | -INF |
| **9** | -26.3 | NA | NA | 8E-14 | 145.9 | 1E-13 | -34.6 | 5E-14 | NA | - | NA | NA | NA | - | NAN | NA | 0 | 0 | 0 | 1 | NA | -INF | -INF |
| **10** | -8.26 | NA | NA | 1E-13 | 22.01 | 1E-13 | -1.79 | 3E-14 | 0.05 | 2E-15 | -3E-4 | NA | NA | 6E-17 | NAN | NA | 0 | 0 | 0 | 1 | NAN | -INF | -INF |
| **11** | 7.87 | NA | NA | 6E-13 | -0.19 | 6E-13 | 2.57 | 1E-13 | -0.25 | 1E-14 | 6E-3 | NA | NA | 3E-16 | NAN | NA | 0 | 0 | 0 | 1 | NAN | -INF | -INF |
| **12** | 25.33 | 3.66 | 0.01 | 12.98 | 2.61 | 9.13 | -0.04 | 2.83 | 3E-4 | 0.36 | -9.8E-7 | 0.70 | 0.51 | 6E-3 | 11.61 | 9E-35 | 82.79 | 851.79 | 769 | 0.90 | 0.82 | 83.8 | 85.6 |
| **13** | 10.39 | 4.85 | 2E-3 | 6.6 | 0.69 | 0.33 | -4E-3 | 6E-3 | 1E-5 | 6E-5 | -1E-9 | -1.16 | 0.28 | 2E-7 | 123 | 6E-6 | 0.42 | 35.73 | 35.30 | 0.98 | 0.98 | 56.0 | 58.4 |
| **14** | 29.67 | 7.73 | 4E-3 | 6.45 | 2.01 | 1.34 | -0.03 | 0.11 | 2E-4 | 3E-3 | -4E-7 | -1.78 | 0.17 | 3E-5 | 42.88 | 5E-35 | 0.84 | 48.51 | 49.35 | 098 | 0.95 | 49.1 | 49.6 |
| **15** | 34.47 | 7.25 | 5E-3 | 9.35 | 2.41 | 1.51 | -0.04 | 0.10 | 2E-4 | 2E-3 | -6E-7 | -2.09 | 0.12 | 2E-5 | 24.54 | 0.01 | 2.27 | 76.73 | 74.45 | 0.97 | 0.93 | 52.5 | 53.0 |
| **16** | 39.59 | 8.01 | 4E-3 | 8.39 | 1.62 | 1.44 | -0.02 | 0.07 | 1E-4 | 1E-3 | -3E-7 | -1.23 | 0.30 | 1E-5 | 17.02 | 0.02 | 3.54 | 80.36 | 83.91 | 0.95 | 0.90 | 53.2 | 53.6 |
| **17** | 44.53 | 13.20 | 9E-4 | 6.12 | 2.18 | 1.05 | -0.04 | 0.05 | 3E-4 | 9E-4 | -7E-7 | -3.25 | 0.04 | 5E-6 | 30.79 | 9E-3 | 0.91 | 37.49 | 38.40 | 0.97 | 0.94 | 47.1 | 47.5 |
| **18** | -562 | -1.40 | 0.39 | 861 | 1963.2 | 3827 | -2005 | 5991 | 807.5 | 3813 | -110.9 | -1.05 | 0.48 | 773 | 3.46 | 0.38 | 32.10 | 477.06 | 444.95 | 0.93 | 0.66 | 54.9 | 53.6 |
| **18*** | 0 | - | - | - | -62.9 | 986.2 | 336.9 | 3711.6 | -202.7 | 4532 | 33.72 | 1.33 | 0.31 | 72.6 | 14.82 | 0.06 | 44.39 | 1317.4 | 1361.8 | 0.96 | 0.90 | 59.4 | 58.3 |
| **19** | -347 | -4.80 | 0.04 | 426 | 1075 | 2139 | -909 | 3954 | 320.17 | 3201 | -40.04 | -2.12 | 0.16 | 964 | 110.7 | 8E-39 | 0.17 | 39.73 | 39.56 | 0.99 | 0.98 | 44.0 | 43.7 |
| **20** | 5.00 | 5.03 | 7E-4 | 4.80 | 4.92 | 3.15 | -0.20 | 0.82 | -1E-3 | 0.10 | 1E-4 | 0.40 | 0.69 | 4E-3 | 506.6 | 1E-10 | 0.02 | 6.77 | 6.80 | 0.99 | 0.99 | 41.6 | 45.4 |
| **21** | 9.06 | 33.48 | 9E-11 | 0.68 | 1.88 | 0.61 | 0.05 | 0.19 | -7E-3 | 0.02 | 1E-4 | 1.34 | 0.21 | 1E-3 | 3610 | 2E-14 | 3E-4 | 0.50 | 0.50 | 0.99 | 0.99 | 5.19 | 9.03 |
| **22** | 9.96 | 7.40 | 4E-5 | 4.04 | 1.82 | 3.31 | 0.18 | 1.00 | -0.01 | 0.12 | 3E-4 | 0.59 | 0.56 | 5E-3 | 237.9 | 4E-9 | 0.11 | 12.46 | 12.58 | 0.99 | 0.98 | 50.1 | 53.9 |
| **23** | 14.46 | 16.60 | 4E-8 | 1.92 | 4.08 | 1.85 | -0.17 | 0.60 | 0.01 | 0.08 | -3E-4 | -0.90 | 0.39 | 3E-3 | 900.4 | 1E-11 | 0.01 | 5.21 | 5.23 | 0.99 | 0.99 | 37.9 | 41.7 |
| **24** | 9.39 | 6.59 | 1E-4 | 3.82 | 5.71 | 3.65 | -0.60 | 1.15 | 0.03 | 0.14 | -8E-4 | -1.40 | 0.19 | 6E-3 | 186.2 | 1E-8 | 0.16 | 13.97 | 14.14 | 0.98 | 0.98 | 51.7 | 55.5 |
| **25** | 12.77 | 10.79 | 1E-6 | 3.11 | 5.04 | 2.84 | -0.36 | 0.90 | 0.02 | 0.11 | -5E-4 | -1.24 | 0.24 | 5E-3 | 559.5 | 8E-11 | 0.03 | 9.63 | 9.67 | 0.99 | 0.99 | 46.5 | 50.3 |
| **26** | 52.84 | 21.25 | 2E-5 | 9.71 | 2.88 | 4.72 | -0.13 | 0.83 | 2E-3 | 0.06 | -9E-6 | -2.83 | 0.04 | 1E-3 | 61.54 | 7E-4 | 0.22 | 14.34 | 14.11 | 0.98 | 0.96 | 41.6 | 42.8 |
| **27** | 38.32 | 13.46 | 0.04 | 7.56 | 6.27 | 2.74 | -0.30 | 0.32 | 5E-3 | 0.01 | -3E-5 | -4.35 | 0.14 | 1E-4 | 103.8 | 0.07 | 6E-3 | 2.80 | 2.80 | 0.99 | 0.98 | 24.5 | 23.2 |
| **28** | 13.59 | 2.96 | 0.20 | 11.19 | 5.24 | 4.20 | -0.15 | 0.51 | 1E-3 | 0.02 | -6E-6 | -0.51 | 0.69 | 2E-4 | 90.25 | 0.07 | 0.02 | 7.28 | 7.26 | 0.99 | 0.98 | 30.2 | 28.9 |
| **28*** | 0 | - | - | - | 8.71 | 2.30 | -0.36 | 0.51 | 6E-3 | 0.02 | -3E-5 | -1.99 | 0.18 | 4E-4 | 151.8 | 6E-3 | 0.23 | 71.12 | 71.36 | 0.99 | 0.99 | 41.9 | 40.8 |
| **29** | 12.20 | 6.49 | 0.09 | 4.38 | 7.15 | 1.68 | -0.34 | 0.21 | 6E-3 | 8E-3 | -4E-5 | -7.60 | 0.08 | 9E-5 | 338.6 | 0.04 | 8E-4 | 1.22 | 1.22 | 0.99 | 0.99 | 19.5 | 18.2 |
| **29*** | 0 | - | - | - | 10.28 | 2.31 | -0.53 | 0.55 | 0.01 | 0.03 | -6E-5 | -4.27 | 0.05 | 7E-4 | 183.1 | 5E-3 | 0.14 | 52.73 | 52.87 | 0.99 | 0.99 | 40.1 | 39.0 |
| **30** | 14.51 | 12.57 | 0.05 | 2.98 | 3.57 | 1.07 | -0.13 | 0.12 | 2E-3 | 4E-3 | -1E-5 | -3.67 | 0.16 | 5E-5 | 489.4 | 0.03 | 2E-4 | 0.46 | 0.46 | 0.99 | 0.99 | 13.6 | 12.4 |
| **31** | 33.28 | 7.55 | 4E-3 | 7.78 | 1.78 | 2.45 | -0.03 | 0.20 | 3E-4 | 5E-3 | -1E-6 | -0.85 | 0.45 | 4E-5 | 13.56 | 0.02 | 3.41 | 65.68 | 65.09 | 0.94 | 0.87 | 51.0 | 51.5 |
| **32** | 30.98 | 6.60 | 7E-3 | 8.99 | 2.09 | 2.64 | -0.04 | 0.20 | 4E-4 | 5E-3 | -1E-6 | -1.01 | 0.38 | 4E-5 | 13.63 | 0.02 | 3.85 | 70.01 | 73.86 | 0.94 | 0.87 | 52.1 | 52.5 |
| **33** | 2.29 | 0.16 | 0.89 | 37.42 | -1.46 | 91.45 | 30.94 | 79.64 | -13.29 | 29.16 | 1.64 | 1.25 | 0.42 | 3.75 | 84.39 | 0.08 | 0.04 | 5.98 | 5.93 | 0.99 | 0.98 | 29.0 | 27.7 |
| **33*** | 0 | - | - | - | 4.92 | 13.33 | 25.52 | 24.88 | -11.53 | 15.56 | 1.45 | 3.43 | 0.07 | 3.18 | 807.4 | 1E-3 | 3E-3 | 6.10 | 6.10 | 0.99 | 0.99 | 27.1 | 26.1 |
| **34** | 3.84 | 0.655 | 0.54 | 50.26 | 21.73 | 40.46 | -1.90 | 12.90 | 0.06 | 1.92 | -7E-4 | -3.54 | 0.02 | 0.11 | 60.25 | 7E-4 | 1.25 | 75.96 | 77.22 | 0.98 | 0.96 | 56.7 | 57.9 |
| **34*** | 0 | - | - | - | 23.50 | 6.28 | -2.11 | 3.98 | 0.07 | 0.83 | -7E-4 | -6.4 | 1E-3 | 0.05 | 670.4 | 5E-7 | 1.39 | 84.10 | 85.49 | 0.99 | 0.99 | 55.7 | 56.6 |
| **35** | 13.30 | 4.40 | 0.02 | 30.25 | 17.21 | 15.38 | -2.25 | 3.02 | 0.17 | 0.27 | -4E-3 | -2.61 | 0.07 | 9E-3 | 628.6 | 1E-4 | 6E-3 | 5.12 | 5.11 | 0.99 | 0.99 | 31.1 | 31.6 |
| **36** | -3.33 | -0.16 | 0.89 | 26.78 | 71.36 | 54.40 | -23.06 | 33.92 | 3.39 | 7.75 | -0.17 | -0.89 | 0.53 | 0.57 | 14.53 | 0.19 | 1.23 | 73.27 | 72.03 | 0.98 | 0.91 | 43.9 | 42.7 |
| **36*** | 0 | - | - | - | 65.69 | 16.04 | -20.4 | 26.9 | 2.95 | 12.63 | -0.15 | -1.67 | 0.23 | 1.61 | 235.6 | 4E-3 | 0.15 | 73.98 | 74.14 | 0.99 | 0.99 | 42.1 | 41.1 |
| **37** | 16.25 | NA | NA | 2E-14 | -0.02 | 8E-16 | 2E-3 | 9E-18 | -1E-5 | 2E-20 | NA | NA | NA | - | NAN | NA | 0 | 0 | 0 | 1 | NAN | -INF | -INF |
| **38** | -6.85 | -2.06 | 0.17 | 12.38 | 12.50 | 26.48 | -2.18 | 18.73 | 0.15 | 4.74 | -3E-3 | -1.84 | 0.20 | 0.36 | 41.15 | 0.02 | 0.12 | 10.51 | 10.39 | 0.98 | 0.96 | 34.6 | 34.3 |
| **38*** | 0 | - | - | - | 5.43 | 6.39 | -0.47 | 8.39 | 0.02 | 4.19 | -4E-4 | -0.25 | 0.81 | 0.69 | 53.31 | 4E-3 | 0.45 | 32.63 | 33.09 | 0.98 | 0.96 | 40.6 | 40.4 |
| **39** | -7.32 | -3.95 | 0.05 | 5.44 | 13.95 | 11.40 | -2.35 | 7.69 | 0.16 | 1.81 | -3E-3 | -3.23 | 0.08 | 0.12 | 156.6 | 6E-33 | 0.01 | 3.24 | 3.23 | 0.99 | 0.99 | 26.5 | 26.1 |
| **40** | -6.17 | -1.80 | 0.21 | 12.51 | 13.63 | 27.16 | -2.08 | 19.33 | 0.13 | 4.88 | -2E-3 | -1.40 | 0.25 | 0.37 | 52.92 | 0.01 | 0.10 | 11.16 | 11.06 | 0.99 | 0.97 | 35.1 | 34.7 |
| **40*** | 0 | - | - | - | 7.27 | 6.87 | -0.54 | 8.71 | 0.01 | 3.92 | 6E-6 | 4E-3 | 0.99 | 0.60 | 92.03 | 1E-3 | 0.23 | 29.06 | 29.30 | 0.99 | 0.98 | 39.8 | 39.6 |

Note: * refers to sets that have been rescaled considering the intercept with a value of zero.

**Polynomial equation of fifth degree by simple linear regression.**

| **No.** | **a** | **t-value** | **p-value** | **ES Boot** | **b** | **ES Boot** | **c** | **ES Boot** | **d** | **ES Boot** | **e** | **ES Boot** | **f** | **t-value** | **p-value** | **ES Boot** | **Est F** | **p-value** | **SSE** | **SSR** | **SST** | **R2** | **R2a** | **AIC** | **BIC** |
| --- | --- | --- | --- | --- | --- | --- | --- | --- | --- | --- | --- | --- | --- | --- | --- | --- | --- | --- | --- | --- | --- | --- | --- | --- | --- |
| **1** | 15.02 | 2.69 | 0.07 | 8.20 | 8.62 | 4.67 | -0.41 | 0.78 | 7E-3 | 0.05 | -5E-5 | 1E-3 | 1E-7 | 3.05 | 0.05 | 8E-6 | 20.46 | 0.01 | 3.07 | 104.92 | 108.0 | 0.97 | 0.92 | 61.6 | 63.0 |
| **1*** | - | - | - | - | 12.39 | 2.74 | -0.63 | 0.63 | 0.01 | 0.04 | -8E-5 | 1E-3 | 2E-7 | 4.05 | 0.01 | 1E-5 | 76.77 | 4E-4 | 3.73 | 358.65 | 362.38 | 0.99 | 0.97 | 70.7 | 71.9 |
| **2** | 21.30 | 3.78 | 0.03 | 13.97 | 6.69 | 10.77 | -0.31 | 2.63 | 5E-3 | 0.24 | -4E-5 | 9E-3 | 1E-7 | 2.15 | 0.12 | 1E-4 | 14.18 | 0.02 | 4.51 | 106.7 | 111.2 | 0.95 | 0.89 | 61.8 | 63.2 |
| **3** | 15.5 | 7.55 | 2E-4 | 4.66 | 2.57 | 1.17 | -0.07 | 0.09 | 9E-4 | 2E-3 | -5E-6 | 4E-5 | 1E-8 | 2.73 | 0.03 | 2E-7 | 83.58 | 1E-5 | 0.60 | 42.06 | 42.67 | 0.98 | 0.97 | 63.1 | 66.5 |
| **3*** | 18.22 | 7.31 | 1E-4 | 4.90 | 1.68 | 0.08 | -0.02 | 0.04 | 2E-4 | 7E-4 | -4E-7 | 4E-6 | 0 | - | - | - | 53.29 | 2E-5 | 3.10 | 97.60 | 94.50 | 0.96 | 0.95 | 70.8 | 73.7 |
| **4** | 94.14 | NA | NA | 1E-11 | -2.68 | 5E-13 | 0.03 | 5E-15 | -1E-4 | 2E-17 | 1E-7 | 2E-10 | NA | NA | NA | - | NAN | NA | 0 | 0 | 0 | 1 | NA | -INF | -INF |
| **5** | 111.7 | NA | NA | 1E-11 | -3.35 | 5E-13 | 0.03 | 5E-15 | -1E-4 | 2E-17 | 1E-7 | 2E-20 | NA | NA | NA | - | NAN | NA | 0 | 0 | 0 | 1 | NA | -INF | -INF |
| **6** | 0.02 | 0.06 | 0.95 | 52.69 | 21.29 | 0.90 | -3.36 | 0.41 | 25.53 | 0.07 | -8E-3 | 4E-3 | 8E-5 | 15.26 | 0.04 | 9E-5 | 1588.0 | 6E-30 | 0 | 0.07 | 0.07 | 1 | 1 | 1.64 | 1.27 |
| **6*** | 0 | - | - | - | 4.74 | 2.43 | 0 | - | 0 | - | 0 | - | 0 | - | - | - | 18.11 | 0.01 | 840.6 | 3806.63 | 4646.97 | 0.81 | 0.77 | 51.4 | 50.6 |
| **7** | 64 | NA | NA | 5E-14 | 36 | 7E-14 | -8 | 2E-14 | NA | - | NA | - | NA | NA | NA | - | NAN | NA | 0 | 0 | 0 | 1 | NA | -INF | -INF |
| **8** | -26.3 | NA | NA | 1E-13 | 145.9 | 2E-13 | -34.6 | 5E-14 | NA | - | NA | - | NA | NA | NA | - | NAN | NA | 0 | 0 | 0 | 1 | NA | -INF | -INF |
| **9** | -8.26 | NA | NA | 8E-14 | 22.01 | 1E-13 | -1.79 | 4E-14 | 0.05 | - | -3E-4 | - | NA | NA | NA | - | NAN | NA | 0 | 0 | 0 | 1 | NAN | -INF | -INF |
| **10** | -8.26 | NA | NA | 1E-13 | 22.01 | 1E-13 | -1.79 | 3E-14 | 0.05 | 2E-15 | -3E-4 | 7E-17 | NA | NA | NA | - | NAN | NA | 0 | 0 | 0 | 1 | NAN | -INF | -INF |
| **11** | 7.87 | NA | NA | 6E-13 | -0.19 | 6E-13 | 2.57 | 1E-13 | -0.25 | 1E-14 | 6E-3 | 3E-16 | NA | NA | NA | - | NAN |  | 0 | 0 | 0 | 1 | NAN | -INF | -INF |
| **12** | 23.63 | 2.70 | 0.05 | 20.05 | 3.46 | 22.30 | -0.09 | 9.91 | 1E-3 | 2.03 | -7E-6 | 0.17 | 1E-8 | 0.38 | 0.71 | 4E-3 | 7.74 | 0.03 | 76.61 | 741.01 | 817.62 | 0.90 | 0.78 | 85.4 | 87.6 |
| **13** | 11.13 | 4.04 | 9E-3 | 15.92 | 0.60 | 1.01 | -2E-3 | 0.02 | -4E-6 | 3E-4 | 4E-8 | 2E-6 | -5E-11 | -0.48 | 0.64 | 5E-9 | 85.93 | 7E-5 | 0.39 | 33.71 | 34.10 | 0.98 | 0.97 | 57.5 | 60.3 |
| **14** | 24.62 | 9.87 | 0.01 | 5.98 | 3.36 | 2.14 | -0.10 | 0.22 | 1E-3 | 9E-3 | -9E-6 | 1E-4 | 1E-8 | 3.16 | 0.08 | 7E-7 | 139.4 | 7E-31 | 0.02 | 8.07 | 8.09 | 0.99 | 0.99 | 36.8 | 37.3 |
| **14*** | 50.33 | 8.02 | 2E-4 | 6.85 | 0.19 | 0.17 | 0 | - | 0 | - | 0 | - | 0 | - | - | - | 9.64 | 0.02 | 673.80 | 1756.52 | 1082.72 | 0.62 | 0.55 | 68.0 | 68.2 |
| **15** | 28.37 | 8.22 | 0.01 | 6.08 | 4.04 | 2.09 | -0.12 | 0.22 | 1E-3 | 9E-3 | -1E-5 | 1E-4 | 2E-8 | 2.76 | 0.10 | 7E-7 | 64.66 | 0.01 | 0.09 | 15.43 | 15.53 | 0.99 | 0.97 | 42.0 | 42.5 |
| **16** | 33.4 | 8.44 | 0.01 | 6.12 | 3.26 | 1.32 | -0.11 | 0.11 | 1E-3 | 4E-3 | -1E-5 | 7E-5 | 2E-8 | 2.42 | 0.13 | 4E-7 | 37 | 0.02 | 0.22 | 20.36 | 20.58 | 0.98 | 0.96 | 44.2 | 44.7 |
| **17** | 40.05 | 19.10 | 2E-3 | 3.02 | 3.38 | 0.65 | -0.10 | 0.04 | 1E-3 | 1E-3 | -8E-6 | 1E-5 | 1E-8 | 3.33 | 0.07 | 4E-8 | 110.1 | 9E-3 | 0.02 | 5.70 | 5.72 | 0.99 | 0.98 | 34.0 | 34.6 |
| **18** | -2818 | NA | NA | 3E-9 | 13391 | 1E-8 | -23096 | 3E-8 | 18218 | 2E-8 | -6413.3 | 13-8 | 815.6 | NA | NA | 1E-9 | NAN | NA | 0 | 0 | 0 | 1.00 | NAN | -INF | -INF |
| **19** | 280.6 | 1.47 | 0.38 | 692 | -2116 | 4088 | 5003 | 9312 | -4576 | 10095 | 1736 | 5084 | -230 | -3.33 | 0.18 | 902 | 538.5 | 0.03 | 1E-3 | 3.26 | 3.26 | 1.00 | 0.99 | 28.5 | 28.2 |
| **19*** | 0 | - | - | - | -699.3 | 560.5 | 2395 | 2945 | -2426 | 5722 | 959 | 4881 | -129 | -9.68 | 0.01 | 1551 | 1575 | 6E-4 | 3E-3 | 10.35 | 10.35 | 1.00 | 0.99 | 34.6 | 34.3 |
| **20** | 5.18 | 3.48 | 8E-3 | 13.44 | 4.63 | 13.40 | -0.08 | 5.35 | -0.01 | 1.08 | 1E-3 | 0.11 | -2E-5 | -0.17 | 0.86 | 4E-3 | 361.6 | 3E-9 | 0.02 | 6.75 | 6.78 | 0.99 | 0.99 | 43.5 | 48.0 |
| **21** | 8.94 | 22.26 | 1E-8 | 3.06 | 2.07 | 2.56 | -0.02 | 0.93 | 5E-3 | 0.17 | -6E-4 | 0.01 | 1E-5 | 0.41 | 0.68 | 6E-4 | 2623 | 1E-12 | 2E-4 | 0.49 | 0.49 | 0.99 | 0.99 | 6.89 | 11.4 |
| **21*** | 12.33 | 10.95 | 1E-7 | 1.70 | 1.20 | 0.33 | 0 | - | 0 | - | 0 | - | 0 | - | - | - | 91.21 | 5E-7 | 12.38 | 106.46 | 94.08 | 0.88 | 0.87 | 72.4 | 74.3 |
| **22** | 6.79 | 5.42 | 6E-4 | 13.73 | 6.89 | 11.40 | -1.89 | 3.86 | 0.30 | 0.66 | -0.02 | 0.05 | 4E-4 | 3.57 | 7E-3 | 2E-3 | 441.4 | 1E-9 | 0.01 | 4.80 | 4.82 | 0.99 | 0.99 | 38.8 | 43.2 |
| **23** | 13.33 | 11.31 | 3E-6 | 9.17 | 5.89 | 9.52 | -0.91 | 3.77 | 0.12 | 0.70 | -7E-3 | 0.06 | 1E-4 | 1.35 | 0.21 | 2E-3 | 786.8 | 1E-10 | 8E-3 | 4.24 | 4.25 | 0.99 | 0.99 | 37.0 | 41.5 |
| **23*** | 20.84 | 10.84 | 1E-7 | 2.86 | 1.92 | 0.56 | 0 | - | 0 | - | 0 | - | 0 | - | - | - | 79.47 | 1E-6 | 41.46 | 316.07 | 274.61 | 0.87 | 0.86 | 87.4 | 89.3 |
| **24** | 6.72 | 4.02 | 3E-3 | 17.49 | 9.98 | 13.35 | -2.35 | 4.49 | 0.30 | 0.83 | -0.01 | 0.08 | 3E-4 | 2.25 | 0.05 | 3E-3 | 217.5 | 2E-8 | 0.06 | 8.55 | 8.61 | 0.99 | 0.98 | 46.8 | 51.3 |
| **24*** | 12.20 | 14.85 | 1E-8 | 1.35 | 3.14 | 0.45 | -0.07 | 0.03 | 0 | - | 0 | - | 0 | - | - | - | 275.4 | 4E-10 | 0.45 | 22.92 | 23.38 | 0.98 | 0.97 | 54.6 | 57.2 |
| **25** | 10.89 | 7.22 | 8E-5 | 13.90 | 8.05 | 11.98 | -1.59 | 4.33 | 0.21 | 0.82 | -0.01 | 0.07 | 2E-4 | 1.75 | 0.11 | 3E-3 | 552.3 | 6E-10 | 0.02 | 6.94 | 6.96 | 0.99 | 0.99 | 43.9 | 48.4 |
| **25*** | 19.65 | 11.08 | 1E-7 | 2.75 | 2.10 | 0.52 | 0 | - | 0 | - | 0 | - | 0 | - | - | - | 111.5 | 2E-7 | 25.16 | 258.90 | 233.73 | 0.90 | 0.89 | 85.1 | 87.1 |
| **26** | 46.88 | 13.57 | 8E-4 | 15.19 | 5.94 | 8.34 | -0.05 | 1.75 | 2E-3 | 17.23 | -3E-4 | 7E-3 | 1.3E-6 | 2.04 | 0.13 | 1E-4 | 89.36 | 1E-38 | 0.04 | 5.88 | 5.92 | 0.993 | 0.982 | 35.7 | 37.1 |
| **27** | 45.37 | NA | NA | 3E-12 | 1.47 | 1E-12 | 0.49 | 3E-13 | -0.03 | 1E-14 | 8E-4 | 3E-16 | -5E-6 | NA | NA | 2E-18 | NAN | NA | 0 | 0 | 0 | 1 | NAN | -INF | -INF |
| **28** | 2.23 | NA | NA | 9E-12 | 12.97 | 5E-12 | -1.43 | 8E-13 | 0.07 | 4E-14 | -1E-3 | 9E-16 | 8E-6 | NA | NA | 5E-18 | NAN | NA | 0 | 0 | 0 | 1 | NAN | -INF | -INF |
| **29** | 7.54 | NA | NA | 5E-12 | 10.33 | 3E-12 | -0.86 | 5E-13 | 0.03 | -1E-14 | -5E-4 | 3E-16 | 3E-6 | NA | NA | 3E-18 | NAN | NA | 0 | 0 | 0 | 1 | NAN | -INF | -INF |
| **30** | 11.65 | NA | NA | 2E-12 | 5.52 | 1E-12 | -0.46 | 1E-13 | 0.02 | 8E-15 | -3E-4 | 1E-16 | 2E-6 | NA | NA | 1E-18 | NAN | NA | 0 | 0 | 0 | 1 | NAN | -INF | -INF |
| **31** | 29.38 | 5.13 | 0.03 | 7.96 | 3.10 | 2.85 | -0.12 | 0.31 | 2E-3 | 0.01 | -2E-5 | 2E-4 | 6E-8 | 1.05 | 0.40 | 1E-6 | 11.42 | 0.08 | 1.39 | 39.83 | 41.23 | 0.96 | 0.88 | 49.5 | 50.1 |
| **32** | 27.03 | 4.32 | 0.04 | 8.35 | 3.43 | 3.14 | -0.13 | 0.35 | 2E-3 | 0.01 | -2E-5 | 2E-4 | 6E-8 | 0.97 | 0.43 | 1E-6 | 10.89 | 0.08 | 1.74 | 47.55 | 49.30 | 0.96 | 0.87 | 51.0 | 51.5 |
| **33** | 47.37 | NA | NA | 1E-11 | -180.3 | 4E-11 | 270.90 | 6E-11 | -153.90 | 3E-11 | 38.43 | 9E-12 | -3.50 | NA | NA | 8E-13 | NAN | NA | 0 | 0 | 0 | 1 | NAN | -INF | -INF |
| **34** | 14.33 | 1.38 | 0.26 | 116.85 | 1.08 | 114.43 | 1.05 | 45.13 | -0.21 | 8.73 | 9E-3 | 0.82 | -1E-4 | -1.2 | 0.31 | 0.03 | 53.82 | 3E-38 | 0.57 | 51.30 | 51.87 | 0.98 | 0.97 | 56.6 | 51.3 |
| **34*** | 0 | - | - | - | 23.33 | 12.46 | -2.04 | 10.57 | 0.06 | 3.29 | -5E-4 | 0.46 | -3E-6 | -0.05 | 0.95 | 0.02 | 429.4 | 1E-5 | 0.06 | 15.71 | 15.78 | 0.99 | 0.99 | 57.6 | 58.8 |
| **35** | 12.80 | 2.04 | 0.17 | 45.8 | 17.91 | 34.87 | -2.52 | 10.12 | 0.21 | 1.40 | -7E-3 | 0.09 | 7E-5 | 0.10 | 0.92 | 2E-3 | 336.90 | 2E-39 | 6E-3 | 5.09 | 5.10 | 0.99 | 0.99 | 33.1 | 33.6 |
| **35*** | 0 | - | - | - | 32.99 | 4.98 | -7.88 | 3.28 | 1.01 | 0.78 | -0.06 | 0.07 | 2E-3 | 2.07 | 0.13 | 2E-3 | 1550 | 2E-5 | 6E-3 | 15.71 | 15.72 | 1.00 | 0.99 | 40.1 | 40.6 |
| **36** | 36.58 | NA | NA | 6E-12 | -42.87 | 1E-11 | 72.50 | 1E-11 | -28.26 | 5E-12 | 4.27 | 7E-13 | -0.22 | NA | NA | 3E-14 | NAN | NA | 0 | 0 | 0 | 1.00 | NAN | -INF | -INF |
| **37** | 16.25 | NA | NA | 1E-14 | -0.02 | 7E-16 | 2E-3 | 8E-18 | -1E-5 | 2E-20 | NA | - | NA | NA | NA | - | NAN | NA | 0 | 0 | 0 | 1.00 | NAN | -INF | -INF |
| **38** | -5.32 | -0.63 | 0.64 | 17.17 | 9.30 | 40.74 | -0.45 | 33.02 | -0.16 | 10.69 | 0.01 | 1.35 | -5E-4 | -0.21 | 0.86 | 0.05 | 17.25 | 0.18 | 0.11 | 9.92 | 10.03 | 0.98 | 0.93 | 36.3 | 35.9 |
| **38*** | 0 | - | - | - | -0.13 | 8.76 | 4.15 | 17.57 | -1.00 | 12.03 | 0.07 | 2.96 | -1E-3 | -1.64 | 0.24 | 0.22 | 67.35 | 0.01 | 0.08 | 13.88 | 13.96 | 0.99 | 0.97 | 36.7 | 36.3 |
| **39** | -4.64 | -1.29 | 0.42 | 7.12 | 8.36 | 17.00 | 0.66 | 13.72 | -0.41 | 4.40 | 0.04 | 0.55 | -9E-4 | -0.88 | 0.53 | 0.02 | 112.2 | 0.07 | 3E-3 | 1.81 | 1.81 | 0.99 | 0.98 | 24.4 | 24.0 |
| **39*** | 0 | - | - | - | 0.12 | 3.53 | 4.69 | 5.90 | -1.13 | 3.72 | 0.08 | 0.94 | -2E-3 | -3.13 | 0.08 | 0.07 | 240.4 | 4E-3 | 8E-3 | 4.82 | 4.83 | 0.99 | 0.99 | 29.3 | 28.9 |
| **40** | -6.13 | -0.69 | 0.61 | 19.08 | 13.55 | 43.84 | -2.04 | 34.22 | 0.12 | 10.70 | -2E-3 | 1.31 | -1.3E | -5E-3 | 0.99 | 0.05 | 21.17 | 0.16 | 0.10 | 11.05 | 11.16 | 0.99 | 0.94 | 37.1 | 36.7 |
| **40*** | 0 | - | - | - | 2.67 | 9.49 | 3.27 | 18.5 | -0.83 | 12.52 | 0.06 | 3.09 | -1E-3 | -1.25 | 0.33 | 0.23 | 87.72 | 0.01 | 0.07 | 16.32 | 16.39 | 0.99 | 0.98 | 37.8 | 37.5 |

Note: * refers to sets that have been rescaled considering the intercept with a value of zero.
