# Supplementary material for: Comparative statistical analysis of the release kinetics models for nanoprecipitated drug delivery systems based on poly(lactic-co-glycolic acid)
Source: PLoS One. 2022 Mar 10;17(3):e0264825. doi: 10.1371/journal.pone.0264825 (PMC8912140; doi:10.1371/journal.pone.0264825)
Supplement: S5 File — (DOCX) [file pone.0264825.s005.docx]

**S5 File.**

**Zero Order Model by Bootstrapping fitted regression**

| **No.** | **Inter_B_** | **IC_95%_** | | **Pend_B_** | **IC_95%_** | | **R^2^_B_** | **IC_95%_** | | **AIC** |
| --- | --- | --- | --- | --- | --- | --- | --- | --- | --- | --- |
| **1** | 47.23 | 31.85 | 60.52 | 0.24 | 0.12 | 0.49 | 0.68 | 0.49 | 0.83 | 52.37 |
| **2** | 46.93 | 35.90 | 58.16 | 0.21 | 0.09 | 0.36 | 0.68 | 0.44 | 0.83 | 49.29 |
| **3** | 32.22 | 26.27 | 42.49 | 0.19 | 0.11 | 0.37 | 0.80 | 0.74 | 0.85 | 57.58 |
| **4** | 23.78 | 13.00 | 43.50 | 0.12 | 0.07 | 0.22 | 0.94 | 0.87 | 0.95 | 23.08 |
| **5** | 19.27 | 9.87 | 38.75 | 0.08 | 0.03 | 0.17 | 0.90 | 0.80 | 0.96 | 22.02 |
| **6** | 28.30 | 17.74 | 50.31 | 1.53 | 0.93 | 3.90 | 0.85 | 0.77 | 0.89 | 41.55 |
| **7** | 76.00 | 76.00 | 88.00 | 8.00 | 4.00 | 8.00 | 0.81 | 0.71 | 0.97 | 11.62 |
| **8** | 44.54 | -9.00 | 77.50 | 19.85 | 7.50 | 24.80 | 0.65 | 0.57 | 0.76 | 20.41 |
| **9** | 31.88 | -13.0 | 59.59 | 23.85 | 13.50 | 28.0 | 0.79 | 0.72 | 0.85 | 19.35 |
| **10** | 30.66 | 6.25 | 65.60 | 2.85 | 0.90 | 6.09 | 0.74 | 0.41 | 0.76 | 31.81 |
| **11** | 16.92 | 8.38 | 46.50 | 2.80 | 1.41 | 5.20 | 0.90 | 0.83 | 0.93 | 28.02 |
| **12** | 36.03 | 25.86 | 46.83 | 0.439 | 0.33 | 0.58 | 0.88 | 0.76 | 0.95 | 55.86 |
| **13** | 26.05 | 20.02 | 36.72 | 0.12 | 0.07 | 0.17 | 0.84 | 0.70 | 0.92 | 51.58 |
| **14** | 48.67 | 40.40 | 63.45 | 0.19 | 0.10 | 0.46 | 0.78 | 0.65 | 0.85 | 43.26 |
| **15** | 55.96 | 45.64 | 71.64 | 0.17 | 0.07 | 0.44 | 0.73 | 0.62 | 0.81 | 43.82 |
| **16** | 53.78 | 47.46 | 64.89 | 0.17 | 0.10 | 0.36 | 0.81 | 0.69 | 0.86 | 38.89 |
| **17** | 62.45 | 54.15 | 74.35 | 0.14 | 0.06 | 0.35 | 0.74 | 0.62 | 0.80 | 39.87 |
| **18** | 44.27 | -22.9 | 82.70 | 22.54 | 5.54 | 38.64 | 0.62 | 0.37 | 0.77 | 43.12 |
| **19** | 31.09 | -69.90 | 75.88 | 26.91 | 7.87 | 159.38 | 0.680 | 0.409 | 0.930 | 49.60 |
| **20** | 13.21 | 8.85 | 20.59 | 1.56 | 1.06 | 2.75 | 0.88 | 0.83 | 0.96 | 48.86 |
| **21** | 11.97 | 9.49 | 15.64 | 1.21 | 0.95 | 1.86 | 0.94 | 0.91 | 0.97 | 30.67 |
| **22** | 13.06 | 9.80 | 18.71 | 1.53 | 1.19 | 2.42 | 0.92 | 0.88 | 0.98 | 40.25 |
| **23** | 20.36 | 15.91 | 25.80 | 1.92 | 1.51 | 3.02 | 0.93 | 0.89 | 0.95 | 45.66 |
| **24** | 17.18 | 13.56 | 23.07 | 1.39 | 1.00 | 2.37 | 0.90 | 0.86 | 0.93 | 42.30 |
| **25** | 18.98 | 15.07 | 24.51 | 2.12 | 1.68 | 3.10 | 0.95 | 0.93 | 0.96 | 43.41 |
| **26** | 64.69 | 59.86 | 67.46 | 0.21 | 0.17 | 0.37 | 0.92 | 0.72 | 0.99 | 27.36 |
| **27** | 61.82 | 50.79 | 71.81 | 0.47 | 0.26 | 0.92 | 0.85 | 0.70 | 0.94 | 27.68 |
| **28** | 35.92 | 23.88 | 57.00 | 0.72 | 0.33 | 1.78 | 0.85 | 0.64 | 0.96 | 32.68 |
| **29** | 38.49 | 27.81 | 50.00 | 0.56 | 0.37 | 1.34 | 0.86 | 0.79 | 0.94 | 29.37 |
| **30** | 28.33 | 22.37 | 35.89 | 0.43 | 0.29 | 0.87 | 0.90 | 0.77 | 0.97 | 24.04 |
| **31** | 43.59 | 35.43 | 50.73 | 0.24 | 0.15 | 0.43 | 0.86 | 0.75 | 0.92 | 33.03 |
| **32** | 42.57 | 35.01 | 51.74 | 0.26 | 0.14 | 0.39 | 0.84 | 0.73 | 0.91 | 34.88 |
| **33** | 5.20 | -0.60 | 20.44 | 15.16 | 10.31 | 21.71 | 0.96 | 0.92 | 0.99 | 22.37 |
| **33*** | 0 | - | - | 17.22 | 16.02 | 20.85 | 0.93 | 0.86 | 0.98 | 22.81 |
| **34** | 52.13 | 40.07 | 72.19 | 1.11 | 0.45 | 2.82 | 0.66 | 0.49 | 0.80 | 55.04 |
| **35** | 32.42 | 25.64 | 44.34 | 4.69 | 3.42 | 5.83 | 0.97 | 0.94 | 0.98 | 31.63 |
| **36** | 37.75 | 28.14 | 67.91 | 9.06 | 4.22 | 12.96 | 0.90 | 0.72 | 0.97 | 33.26 |
| **37** | 13.07 | 11.77 | 21.50 | 0.15 | 0.12 | 0.19 | 0.99 | 0.98 | 1.00 | 4.43 |
| **38** | 5.15 | 1.46 | 14.66 | 1.22 | 0.77 | 2.97 | 0.85 | 0.74 | 0.90 | 28.32 |
| **38*** | 0 | - | - | 1.61 | 1.27 | 3.09 | 0.73 | 0.60 | 0.82 | 30.05 |
| **39** | 5.80 | 2.80 | 15.81 | 1.39 | 0.89 | 2.42 | 0.88 | 0.80 | 0.93 | 28.31 |
| **39*** | 0 | - | - | 1.81 | 1.48 | 3.06 | 0.80 | 0.59 | 0.85 | 30.89 |
| **40** | 7.59 | 2.84 | 16.95 | 1.44 | 0.93 | 3.61 | 0.87 | 0.78 | 0.91 | 29.98 |
| **40*** | 0 | - | - | 2.01 | 1.60 | 3.40 | 0.77 | 0.41 | 0.86 | 33.10 |

Note: * refers to sets that have been rescaled considering the intercept with a value of zero.

**First-order regression model adjusted by Bootstrapping.**

| **No.** | **Inter_B_** | **IC_95%_** | | **Pend_B_** | **IC_95%_** | | **R^2^_B_** | **IC_95%_** | | **AIC** |
| --- | --- | --- | --- | --- | --- | --- | --- | --- | --- | --- |
| **1** | 3.76 | 3.34 | 4.06 | -4E-3 | -0.01 | -1E-3 | 0.36 | 0.15 | 0.51 | -13.25 |
| **2** | 3.78 | 3.42 | 4.02 | -4E-3 | -8E-3 | -1E-3 | 0.40 | 0.13 | 0.46 | -16.81 |
| **3** | 3.41 | 3.15 | 3.73 | -4E-3 | -0.01 | -2E-3 | 0.54 | 0.29 | 0.53 | -23.17 |
| **4** | 3.28 | 2.88 | 3.80 | -2E-3 | -5E-3 | -1E-3 | 0.85 | 0.60 | 0.70 | -11.21 |
| **5** | 3.03 | 2.67 | 3.69 | -2E-3 | -5E-3 | -7e-4 | 0.79 | 0.50 | 0.81 | -11.23 |
| **6** | 3.18 | 2.64 | 3.81 | -0.03 | -0.09 | -0.01 | 0.57 | 0.29 | 0.56 | -4.86 |
| **7** | 4.33 | 4.24 | 4.48 | -0.08 | -0.27 | -0.04 | 1.00 | 0.65 | 0.65 | -14.96 |
| **8** | 3.77 | 2.83 | 4.36 | -0.29 | -1.61 | -0.08 | 1.00 | 0.41 | 1.00 | -3.80 |
| **9** | 3.16 | 2.51 | 4.13 | -0.48 | -0.48 | -0.15 | 1.00 | 0.51 | 1.00 | -3.45 |
| **10** | 3.19 | 2.51 | 4.20 | -0.06 | -0.17 | -0.01 | 0.60 | 0.27 | 0.44 | -2.40 |
| **11** | 2.84 | 2.12 | 3.84 | -0.07 | -0.17 | -0.02 | 0.72 | 0.37 | 0.61 | -3.73 |
| **12** | 3.48 | 3.11 | 3.80 | -8E-3 | -0.01 | -5E-3 | 0.59 | 0.33 | 0.70 | -14.24 |
| **13** | 3.19 | 2.82 | 3.53 | -3E-3 | -6E-3 | -1E-3 | 0.61 | 0.37 | 0.66 | -20.64 |
| **14** | 3.84 | 3.68 | 4.15 | -3E-3 | -7E-3 | -1E-3 | 0.59 | 0.30 | 0.60 | -19.31 |
| **15** | 3.98 | 3.80 | 4.24 | -2E-3 | -6e-4 | -9e-4 | 0.52 | 0.25 | 0.53 | -20.38 |
| **16** | 3.97 | 3.82 | 4.16 | -2E-3 | -6E-3 | -1E-3 | 0.63 | 0.37 | 0.61 | -24.77 |
| **17** | 4.11 | 3.99 | 4.30 | -2E-3 | -4E-3 | -7e-4 | 0.55 | 0.27 | 0.51 | -26.04 |
| **18** | 3.24 | -0.6 | 4.38 | -0.58 | -1.37 | -0.04 | 0.34 | 0.11 | 0.30 | 2.89 |
| **19** | 2.96 | -1.6 | 4.25 | -0.67 | -3.06 | -0.09 | 0.32 | 0.11 | 0.35 | 4.16 |
| **20** | 2.56 | 2.28 | 2.92 | -0.07 | -0.14 | -0.04 | 0.65 | 0.48 | 0.77 | -28.16 |
| **21** | 2.53 | 2.36 | 2.80 | -0.05 | -0.10 | -0.04 | 0.78 | 0.67 | 0.83 | -4.34 |
| **22** | 2.61 | 2.43 | 2.97 | -0.06 | -0.11 | -0.04 | 0.73 | 0.61 | 0.80 | -38.65 |
| **23** | 3.03 | 2.88 | 3.35 | -0.05 | -0.10 | -0.03 | 0.77 | 0.61 | 0.78 | -43.02 |
| **24** | 2.84 | 2.63 | 3.10 | -0.05 | -0.10 | -0.03 | 0.71 | 0.55 | 0.71 | -38.52 |
| **25** | 3.00 | 2.83 | 3.25 | -0.06 | -0.10 | -0.04 | 0.79 | 0.63 | 0.79 | -41.53 |
| **26** | 4.17 | 4.11 | 4.21 | -2E-3 | -4E-3 | -2E-3 | 0.79 | 0.43 | 0.97 | -46.52 |
| **27** | 4.10 | 3.93 | 4.17 | -6E-3 | -0.01 | -3E-3 | 0.70 | 0.40 | 0.74 | -21.56 |
| **28** | 3.55 | 3.24 | 3.97 | -0.01 | -0.04 | -6E-3 | 0.66 | 0.00 | 0.69 | -11.72 |
| **29** | 3.62 | 3.24 | 3.90 | -0.01 | -0.03 | -5E-3 | 0.67 | 0.40 | 0.68 | -13.71 |
| **30** | 3.33 | 3.11 | 3.59 | -0.01 | -0.02 | -6E-3 | 0.72 | 0.44 | 0.79 | -16.39 |
| **31** | 3.76 | 3.56 | 3.91 | -4E-3 | -8E-3 | -2E-3 | 0.69 | 0.42 | 0.74 | -26.95 |
| **32** | 3.74 | 3.49 | 3.92 | -5E-3 | -0.01 | -2E-3 | 0.66 | 0.41 | 0.73 | -24.77 |
| **33** | 2.30 | 1.72 | 3.25 | -0.51 | -1.06 | -0.21 | 0.80 | 0.54 | 0.79 | -9.06 |
| **34** | 3.88 | 3.60 | 4.24 | -0.01 | -0.04 | -6E-3 | 0.41 | 0.14 | 0.41 | -15.52 |
| **35** | 3.56 | 3.31 | 3.84 | -0.07 | -0.11 | -0.04 | 0.87 | 0.72 | 0.86 | -25.13 |
| **36** | 3.64 | 3.39 | 4.23 | -0.14 | -0.24 | -0.04 | 0.75 | 0.41 | 0.85 | -13.22 |
| **37** | 2.71 | 2.65 | 3.18 | -6E-3 | -7E-3 | -2E-3 | 0.99 | 0.90 | 1.00 | -19.04 |
| **38** | 2.68 | 2.44 | 3.21 | -0.05 | -0.12 | -0.02 | 0.65 | 0.39 | 0.64 | -11.62 |
| **39** | 2.74 | 2.47 | 3.20 | -0.05 | -012 | -0.02 | 0.66 | 0.37 | 0.67 | -11.81 |
| **40** | 1.61 | 0.51 | 2.75 | -0.11 | -0.32 | -0.03 | 0.49 | 0.20 | 0.50 | 3.20 |

Note: * refers to sets that have been rescaled considering the intercept with a value of zero.

**Korsmeyer Peppas model considering 100% of the regression release curve adjusted by Bootstrapping.**

| **No.** | **Inter_B_** | **IC_95%_** | | **Pend_B_** | **IC_95%_** | | **R^2^_B_** | **IC_95%_** | | **AIC** |
| --- | --- | --- | --- | --- | --- | --- | --- | --- | --- | --- |
| **1** | 3.33 | 3.03 | 3.82 | 0.24 | 0.11 | 0.34 | 0.87 | 0.79 | 0.92 | -22.41 |
| **2** | 3.44 | 3.15 | 3.78 | 0.19 | 0.10 | 0.31 | 0.87 | 0.81 | 0.92 | -26.10 |
| **3** | 2.86 | 1.75 | 3.11 | 0.27 | 0.20 | 0.29 | 0.98 | 0.96 | 0.99 | -55.41 |
| **4** | 1.04 | 0.47 | 2.07 | 0.54 | 0.37 | 0.76 | 0.98 | 0.87 | 1.00 | -20.56 |
| **4*** | 0 | - | - | 0.75 | 0.72 | 0.78 | 0.98 | 0.87 | 1.00 | -15.09 |
| **5** | 1.01 | -0.47 | 2.13 | 0.50 | 0.33 | 0.95 | 0.95 | 0.84 | 0.99 | -17.06 |
| **5*** | 0 | - | - | 0.70 | 0.66 | 0.73 | 0.88 | 0.70 | 0.96 | -14.28 |
| **6** | 2.89 | 2.73 | 3.38 | 0.47 | 0.30 | 0.59 | 0.96 | 0.93 | 0.98 | -19.35 |
| **7** | 4.48 | 4.46 | 4.52 | 0.12 | 0.07 | 0.12 | 0.81 | 0.81 | 0.81 | -18.13 |
| **8** | 4.16 | 4.01 | 4.44 | 0.54 | 0.14 | 0.54 | 0.65 | 0.65 | 0.65 | -5.61 |
| **9** | 4.29 | 3.86 | 4.29 | 0.67 | 0.28 | 0.67 | 0.79 | 0.79 | 0.79 | -5.71 |
| **10** | 2.76 | 2.50 | 3.94 | 0.62 | 0.16 | 0.96 | 0.92 | 0.86 | 0.95 | -8.90 |
| **11** | 2.40 | 2.29 | 3.41 | 0.67 | 0.30 | 0.90 | 0.97 | 0.88 | 0.99 | -13.21 |
| **12** | 3.15 | 3.00 | 3.47 | 0.29 | 0.21 | 0.32 | 0.94 | 0.86 | 0.99 | -28.71 |
| **13** | 2.07 | 1.94 | 2.35 | 0.36 | 0.32 | 0.41 | 0.98 | 0.91 | 0.99 | -46.53 |
| **14** | 3.34 | 3.25 | 3.62 | 0.22 | 0.15 | 0.26 | 0.97 | 0.93 | 0.99 | -37.38 |
| **15** | 3.53 | 3.44 | 3.92 | 0.19 | 0.10 | 0.23 | 0.95 | 0.89 | 0.98 | -35.01 |
| **16** | 3.59 | 3.53 | 3.80 | 0.16 | 0.12 | 0.19 | 0.97 | 0.91 | 0.99 | -43.47 |
| **17** | 3.80 | 3.72 | 4.03 | 0.13 | 0.08 | 0.17 | 0.96 | 0.91 | 0.98 | -41.20 |
| **18** | 4.06 | 2.89 | 4.49 | 1.00 | 0.11 | 3.75 | 0.60 | 0.46 | 0.73 | 2.19 |
| **19** | 3.89 | 2.90 | 4.46 | 1.21 | 0.21 | 4.09 | 0.63 | 0.49 | 0.73 | 2.70 |
| **20** | 2.33 | 2.24 | 2.44 | 0.50 | 0.44 | 0.56 | 0.98 | 0.94 | 0.99 | -64.15 |
| **21** | 2.38 | 2.29 | 2.47 | 0.37 | 0.32 | 0.42 | 0.98 | 0.96 | 0.99 | -69.71 |
| **22** | 2.46 | 2.38 | 2.52 | 0.41 | 0.37 | 0.46 | 0.98 | 0.96 | 0.99 | -66.99 |
| **23** | 2.90 | 2.83 | 2.96 | 0.36 | 0.33 | 0.40 | 0.98 | 0.97 | 0.99 | -74.29 |
| **24** | 2.69 | 2.65 | 2.76 | 0.37 | 0.32 | 0.39 | 0.99 | 0.98 | 0.99 | -81.26 |
| **25** | 2.85 | 2.77 | 2.92 | 0.39 | 0.35 | 0.43 | 0.98 | 0.98 | 0.99 | -74.89 |
| **26** | 3.99 | 3.95 | 4.08 | 0.10 | 0.05 | 0.12 | 0.95 | 0.87 | 0.99 | -54.72 |
| **27** | 3.86 | 3.79 | 4.08 | 0.15 | 0.09 | 0.19 | 0.96 | 0.87 | 0.98 | -31.30 |
| **28** | 3.02 | 2.85 | 3.37 | 0.34 | 0.24 | 0.46 | 0.96 | 0.74 | 0.99 | -21.91 |
| **29** | 3.19 | 2.96 | 3.51 | 0.28 | 0.18 | 0.37 | 0.95 | 0.91 | 0.97 | -22.55 |
| **30** | 2.94 | 2.82 | 3.12 | 0.26 | 0.21 | 0.33 | 0.98 | 0.84 | 0.99 | -29.01 |
| **31** | 3.46 | 3.34 | 3.64 | 0.16 | 0.11 | 0.19 | 0.97 | 0.91 | 0.98 | -40.84 |
| **32** | 3.41 | 3.27 | 3.65 | 0.18 | 0.11 | 0.21 | 0.96 | 0.91 | 0.98 | -37.54 |
| **33** | 2.93 | 2.78 | 3.10 | 0.98 | 0.57 | 1.22 | 0.97 | 0.93 | 0.99 | -19.02 |
| **34** | 3.53 | 3.31 | 3.95 | 0.32 | 0.15 | 0.50 | 0.87 | 0.73 | 0.95 | -24.93 |
| **35** | 3.36 | 3.33 | 3.42 | 0.45 | 0.42 | 0.47 | 0.99 | 0.98 | 0.99 | -56.11 |
| **36** | 3.77 | 3.72 | 4.18 | 0.44 | 0.20 | 0.49 | 0.96 | 0.85 | 1.00 | -22.25 |
| **37** | 1.35 | 0.75 | 1.57 | 0.44 | 0.42 | 0.57 | 0.98 | 0.96 | 1.00 | -19.04 |
| **38** | 1.31 | 2.48 | 2.84 | 0.39 | 0.26 | 0.47 | 0.96 | 0.88 | 0.98 | -23.28 |
| **39** | 1.49 | 2.60 | 2.91 | 0.39 | 0.29 | 0.49 | 0.97 | 0.91 | 0.99 | -25.36 |
| **40** | 1.34 | 0.91 | 2.39 | 0.90 | 0.39 | 1.32 | 0.90 | 0.81 | 0.97 | -4.81 |

Note: * refers to sets that have been rescaled considering the intercept with a value of zero.

**Korsmeyer Peppas model considering 60% of the regression release curve adjusted by Bootstrapping.**

| **No.** | **Inter_B_** | **IC_95%_** | | **Pend_B_** | **IC_95%_** | | **R^2^_B_** | **IC_95%_** | | **AIC** |
| --- | --- | --- | --- | --- | --- | --- | --- | --- | --- | --- |
| **1** | 2.96 | 2.89 | 3.48 | 0.59 | 0.46 | 0.83 | 0.97 | 0,91 | 0,95 | -15,19 |
| **2** | 3.18 | 3.04 | 3.56 | 0.42 | 0.23 | 0.61 | 0.93 | 0,79 | 0,92 | -17,39 |
| **3** | 2.83 | 2.74 | 3.07 | 0.28 | 0.22 | 0.30 | 0.97 | 0,91 | 0,98 | -52,27 |
| **4** | 0.58 | -0.01 | 1.93 | 0.66 | 0.40 | 0.77 | 0.98 | 0,72 | 1,00 | -18,18 |
| **4*** | 0 | - | - | 0.77 | 0.76 | 0.79 | 0.95 | 0,47 | 0,99 | -16,98 |
| **5** | 1.01 | -0.47 | 2.13 | 0.50 | 0.33 | 0.95 | 0.95 | 0,84 | 0,99 | -17,06 |
| **5*** | 0 | - | - | 0.70 | 0.66 | 0.72 | 0.88 | 0,74 | 0,97 | -14,28 |
| **6** | 2.84 | 2.74 | 3.04 | 0.59 | 0.35 | 0.70 | 0.97 | 0,90 | 0,98 | -16,73 |
| **7** | - | - | - | - | - | - | - | - | - | - |
| **8** | - | - | - | - | - | - | - | - | - | - |
| **9** | - | - | - | - | - | - | - | - | - | - |
| **10** | - | - | - | - | - | - | - | - | - | - |
| **11** | 2.32 | 2.26 | 3.37 | 0.78 | 0.68 | 1.11 | 0.99 | 0,81 | 0,99 | -12,99 |
| **12** | 3.09 | 3.02 | 3.85 | 0.35 | 0.20 | 0.56 | 0.83 | 0,25 | 0,90 | -13,82 |
| **13** | 2.07 | 1.94 | 2.35 | 0.36 | 0.32 | 0.41 | 0.98 | 0,91 | 0,99 | -46,53 |
| **14** | 3.20 | 2.91 | 3.38 | 0.29 | 0.22 | 0.43 | 0.97 | 0,86 | 1,00 | -20,61 |
| **15** | 3.29 | 3.09 | 3.44 | 0.33 | 0.21 | 0.42 | 1.00 | 0,93 | 1,00 | -15,40 |
| **16** | 3.43 | 3.34 | 3.47 | 0.27 | 0.23 | 0.31 | 1.00 | 0,98 | 1,00 | -21,10 |
| **17** | 3.62 | 3.59 | 3.64 | 0.24 | 0.22 | 0.25 | 1.00 | 0,99 | 1,00 | -26,82 |
| **18** | - | - | - | - | - | - | - | - | - | - |
| **19** | 7.58 | 5.75 | 9.85 | 9.06 | 4.60 | 12.62 | 1.00 | 0,88 | 1,00 | -2,91 |
| **20** | 2.33 | 2.24 | 2.44 | 0.50 | 0.44 | 0.56 | 0.98 | 0,94 | 0,99 | -64,15 |
| **21** | 2.38 | 2.29 | 2.47 | 0.37 | 0.32 | 0.42 | 0.98 | 0,96 | 0,99 | -69,71 |
| **22** | 2.46 | 2.38 | 2.52 | 0.41 | 0.37 | 0.46 | 0.98 | 0,96 | 0,99 | -66,99 |
| **23** | 2.90 | 2.83 | 2.96 | 0.36 | 0.33 | 0.40 | 0.98 | 0,97 | 0,99 | -74,29 |
| **24** | 2.69 | 2.65 | 2.76 | 0.37 | 0.32 | 0.39 | 0.99 | 0,98 | 0,99 | -81,26 |
| **25** | 2.85 | 2.77 | 2.92 | 0.39 | 0.35 | 0.43 | 0.98 | 0,98 | 0,99 | -74,89 |
| **26** | - | - | - | - | - | - | - | - | - | - |
| **27** | - | - | - | - | - | - | - | - | - | - |
| **28** | 2.79 | 2.75 | 2.79 | 0.50 | 0.23 | 0.54 | 1.00 | 0,97 | 1,00 | -16,76 |
| **29** | 3.03 | 2.86 | 3.47 | 0.37 | 0.22 | 0.53 | 0.98 | 0,89 | 0,90 | -15,33 |
| **30** | 2.94 | 2.82 | 3.12 | 0.26 | 0.21 | 0.33 | 0.98 | 0,84 | 0,99 | -29,01 |
| **31** | 3.41 | 3.25 | 3.64 | 0.19 | 0.11 | 0.22 | 0.94 | 0,81 | 0,95 | -23,68 |
| **32** | 3.29 | 3.15 | 3.69 | 0.24 | 0.10 | 0.36 | 0.93 | 0,83 | 0,90 | -16,67 |
| **33** | 2.93 | 2.78 | 3.10 | 0.98 | 0.57 | 1.22 | 0.97 | 0,93 | 0,99 | -19,02 |
| **34** | 3.24 | 3.11 | 3.38 | 0.62 | 0.38 | 0.73 | 0.99 | 0,93 | 1,00 | -22,07 |
| **35** | 3.35 | 3.33 | 3.41 | 0.47 | 0.42 | 0.49 | 0.99 | 0,98 | 0,99 | -27,24 |
| **36** | - | - | - | - | - | - | - | - | - | - |
| **37** | 1.35 | 0.75 | 1.57 | 0.44 | 0.42 | 0.57 | 0.98 | 0,96 | 1,00 | -19,04 |
| **38** | 1.31 | 2.48 | 2.84 | 0.39 | 0.26 | 0.47 | 0.96 | 0,88 | 0,98 | -23,28 |
| **39** | 1.49 | 2.60 | 2.91 | 0.39 | 0.29 | 0.49 | 0.97 | 0,91 | 0,99 | -25,36 |
| **40** | 1.34 | 0.91 | 2.39 | 0.90 | 0.39 | 1.32 | 0.90 | 0,81 | 0,97 | -4,81 |

Note: * refers to sets that have been rescaled considering the intercept with a value of zero.

**Weibull regression model adjusted by Bootstrapping.**

| **No.** | **Inter_B_** | **IC_95%_** | | **Pend_B_** | **IC_95%_** | | **R^2^_B_** | **IC_95%_** | | **AIC** |
| --- | --- | --- | --- | --- | --- | --- | --- | --- | --- | --- |
| **1** | -1.11 | -1.45 | -0.56 | 0.35 | 0.21 | 0.51 | 0.91 | 0.85 | 0.95 | -19.73 |
| **2** | -0.99 | -1.28 | -0.60 | 0.29 | 0.19 | 0.41 | 0.91 | 0.87 | 0.94 | -23.15 |
| **3** | -1.69 | -1.78 | -1.48 | 0.34 | 0.29 | 0.36 | 0.99 | 0.97 | 0.99 | -58.60 |
| **4** | -4.50 | -5.31 | -3.84 | 0.81 | 0.70 | 0.97 | 0.99 | 0.89 | 1.00 | -20.95 |
| **5** | -4.02 | -4.84 | -2.90 | 0.63 | 0.44 | 1.09 | 0.96 | 0.82 | 1.00 | -15.43 |
| **6** | 0.11 | -1.71 | -1.30 | 0.67 | 0.55 | 0.73 | 0.99 | 0.98 | 0.99 | -25.41 |
| **7** | 0.92 | - | - | 0.65 | - | - | - | - | - | - |
| **8** | 0.64 | - | - | 1.98 | - | - | - | - | - | - |
| **9** | 0.26 | - | - | 1.87 | - | - | - | - | - | - |
| **10** | -1.81 | -2.03 | -0.54 | 0.88 | 0.40 | 1.25 | 0.96 | 0.93 | 0.98 | -9.23 |
| **11** | -2.20 | -2.25 | -1.28 | 0.87 | 0.57 | 1.08 | 0.98 | 0.93 | 1.00 | -14.29 |
| **12** | -1.33 | -1.49 | -0.94 | 0.43 | 0.32 | 0.47 | 0.96 | 0.84 | 0.99 | -22.10 |
| **13** | -2.63 | -2.73 | -2.27 | 0.45 | 0.41 | 0.49 | 0.98 | 0.93 | 0.99 | -44.79 |
| **14** | -1.24 | -1.40 | -1.05 | 0.37 | 0.31 | 0.41 | 0.99 | 0.96 | 0.99 | 37.02 |
| **15** | -0.99 | -1.11 | -0.58 | 0.34 | 0.25 | 0.40 | 0.98 | 0.96 | 0.99 | -33.11 |
| **16** | -0.91 | -0.95 | -0.65 | 0.30 | 0.24 | 0.31 | 0.99 | 0.96 | 0.99 | -41.73 |
| **17** | -0.63 | -0.70 | -0.34 | 0.27 | 0.21 | 0.30 | 0.98 | 0.95 | 0.99 | -38.34 |
| **18** | 0.73 | -1.10 | 1.25 | 2.12 | 0.44 | 6.11 | 0.70 | 0.57 | 0.75 | 5.11 |
| **18*** | 0 | - | - | 1.89 | 0.69 | 4.06 | 0.47 | 0.03 | 0.79 | 3.38 |
| **19** | 0.41 | -1.329 | 1.532 | 2.659 | 0.78 | 6.98 | 0.76 | 0.57 | 0.82 | 5.19 |
| **19*** | 0 | - | - | 2.32 | 1.21 | 4.55 | 0.57 | 0.03 | 0.82 | 3.32 |
| **20** | -2.21 | -2.32 | -2.10 | 0.56 | 0.50 | 0.62 | 0.98 | 0.96 | 0.99 | -62.80 |
| **21** | -2.16 | -2.28 | -2.06 | 0.41 | 0.36 | 0.48 | 0.98 | 0.95 | 0.99 | -64.97 |
| **22** | -2.08 | -2.18 | -2.00 | 0.47 | 0.42 | 0.53 | 0.98 | 0.96 | 0.99 | -61.11 |
| **23** | -1.61 | -1.72 | -1.52 | 0.450 | 0.39 | 0.51 | 0.98 | 0.96 | 0.99 | -64.26 |
| **24** | -1.84 | -1.87 | -1.78 | 0.42 | 0.40 | 0.44 | 0.99 | 0.98 | 0.99 | -79.78 |
| **25** | -1.66 | -1.78 | -1.58 | 0.48 | 0.42 | 0.54 | 0.98 | 0.97 | 0.99 | -63.79 |
| **26** | -0.39 | -0.58 | -0.11 | 0.25 | 0.12 | 0.31 | 0.92 | 0.80 | 0.95 | -32.70 |
| **27** | -0.54 | -0.61 | -0.29 | 0.33 | 0.26 | 0.36 | 0.98 | 0.86 | 1.00 | -27.57 |
| **28** | -1.58 | -1.79 | -1.19 | 0.50 | 0.43 | 0.65 | 0.97 | 0.77 | 1.00 | -20.11 |
| **29** | -1.35 | -1.68 | -1.03 | 0.40 | 0.30 | 0.49 | 0.97 | 0.94 | 0.99 | -22.12 |
| **30** | -1.60 | -1.70 | -1.42 | 0.34 | 0.28 | 0.41 | 0.98 | 0.83 | 0.99 | -28.44 |
| **31** | -0.99 | -1.12 | -0.81 | 0.24 | 0.19 | 0.27 | 0.98 | 0.94 | 0.99 | -38.61 |
| **32** | -1.05 | -1.21 | -0.80 | 0.26 | 0.19 | 0.30 | 0.97 | 0.92 | 0.99 | -35.32 |
| **33** | -1.53 | -1.64 | -1.36 | 1.171 | 0.98 | 1.43 | 0.98 | 0.94 | 0.99 | -20.40 |
| **34** | -0.93 | -1.17 | -0.40 | 0.57 | 0.38 | 0.80 | 0.92 | 0.83 | 0.98 | -20.17 |
| **35** | -1.26 | -2.15 | -1.14 | 0.86 | 0.70 | 1.23 | 0.96 | 0.93 | 0.98 | -21.88 |
| **36** | -0.46 | -0.60 | -0.31 | 0.93 | 0.75 | 1.06 | 0.98 | 0.81 | 1.00 | -13.61 |
| **36*** | 0 | - | - | 0.704 | 0.423 | 1.00 | 0.67 | 0.41 | 0.91 | -6.83 |
| **37** | -3.40 | -4.12 | -3.26 | 0.515 | 0.48 | 0.67 | 0.98 | 0.96 | 1.00 | -17.37 |
| **38** | -4.40 | -5.28 | -2.50 | 1.38 | 0.49 | 2.15 | 0.87 | 0.74 | 0.97 | 3.96 |
| **39** | -4.06 | -5.24 | -2.50 | 1.29 | 0.56 | 2.37 | 0.84 | 0.76 | 0.90 | 4.52 |
| **40** | -3.22 | -3.64 | -2.15 | 0.95 | 0.45 | 1.38 | 0.91 | 0.83 | 0.98 | -4.80 |

Note: * refers to sets that have been rescaled considering the intercept with a value of zero.

**Hyperbolic Tangent Function Model by Bootstrapping Fitted Regression**

| **No.** | **Inter_B_** | **IC_95%_** | | **Pend_B_** | **IC_95%_** | | **R^2^_B_** | **IC_95%_** | | **AIC** |
| --- | --- | --- | --- | --- | --- | --- | --- | --- | --- | --- |
| **1** | 0.38 | 0.21 | 0.59 | 0.06 | 0.03 | 0.08 | 0.87 | 0.78 | 0.95 | -30.68 |
| **2** | 0.40 | 0.25 | 0.56 | 0.05 | 0.03 | 0.07 | 0.85 | 0.74 | 0.93 | -33.52 |
| **3** | 0.19 | 0.14 | 0.28 | 0.04 | 0.03 | 0.05 | 0.96 | 0.94 | 0.97 | -67.24 |
| **4** | -0.20 | -0.45 | -0.15 | 0.06 | 0.05 | 0.07 | 0.99 | 0.91 | 1.00 | -30.05 |
| **4*** | 0 | - | - | 0.04 | 0.03 | 0.05 | 0.90 | 0.73 | 0.97 | -23.30 |
| **5** | -0.02 | -0.12 | 0.16 | 0.03 | 0.02 | 0.04 | 0.95 | 0.78 | 0.99 | -26.45 |
| **5*** | 0 | - | - | 0.03 | 0.02 | 0.03 | 0.98 | 0.97 | 0.99 | -23.30 |
| **6** | -0.01 | -0.06 | 0.08 | 0.22 | 0.20 | 0.25 | 0.99 | 0.98 | 0.99 | -38.34 |
| **6*** | 0 | - | - | 0.22 | 0.21 | 0.23 | 0.99 | 0.96 | 0.99 | -40.32 |
| **7** | -0.08 | - | - | 1.67 | - | - | - | - | - | - |
| **8** | -1.66 | - | - | 2.92 | - | - | - | - | ­- | - |
| **9** | -1.18 | - | - | 2.11 | - | - | - | - | - | - |
| **10** | -0.11 | -0.27 | 0.37 | 0.31 | 0.20 | 0.41 | 0.97 | 0.94 | 0.98 | -18.23 |
| **10*** | 0 | - | - | 0.29 | 0.27 | 0.32 | 0.98 | 0.89 | 0.99 | -19.73 |
| **11** | -0.14 | -0.23 | 0.08 | 0.25 | 0.20 | 0.31 | 0.98 | 0.94 | 1.00 | -25.29 |
| **11*** | 0 | - | - | 0.21 | 0.17 | 0.22 | 0.92 | 0.65 | 0.99 | -23.07 |
| **12** | 0.18 | 0.10 | 0.32 | 0.1 | 0.08 | 0.12 | 0.96 | 0.84 | 0.99 | -34.88 |
| **13** | 0.11 | 0.06 | 0.22 | 0.03 | 0.02 | 0.04 | 0.95 | 0.90 | 0.98 | -58.51 |
| **14** | 0.31 | 0.22 | 0.53 | 0.07 | 0.05 | 0.09 | 0.96 | 0.90 | 0.98 | -34.36 |
| **15** | 0.41 | 0.28 | 0.64 | 0.07 | 0.05 | 0.10 | 0.94 | 0.89 | 0.96 | -30.69 |
| **16** | 0.40 | 0.34 | 0.53 | 0.06 | 0.05 | 0.07 | 0.97 | 0.87 | 0.99 | -40.70 |
| **17** | 0.54 | 0.43 | 0.74 | 0.06 | 0.04 | 0.08 | 0.94 | 0.89 | 0.96 | -33.19 |
| **18** | -1.01 | -4.18 | 1.73 | 2.19 | 1.17 | 6.60 | 0.82 | 0.57 | 0.93 | -3.66 |
| **19** | -1.72 | -5.56 | 0.65 | 2.88 | 1.90 | 8.31 | 0.86 | 0.61 | 0.98 | -4.56 |
| **20** | 0.01 | -0.02 | 0.07 | 0.10 | 0.08 | 0.12 | 0.97 | 0.94 | 0.99 | -99.06 |
| **20*** | 0 | - | - | 0.10 | 0.09 | 0.11 | 0.99 | 0.98 | 0.99 | -100.1 |
| **21** | 0.03 | 0.01 | 0.04 | 0.07 | 0.06 | 0.08 | 0.99 | 0.97 | 0.99 | -121.2 |
| **22** | 0.02 | -2E-3 | 0.03 | 0.09 | 0.08 | 0.10 | 0.98 | 0.96 | 0.99 | -105.1 |
| **22*** | 0 | - | - | 0.10 | 0.09 | 0.10 | 0.99 | 0.98 | 0.99 | -104.6 |
| **23** | 0.04 | 0.02 | 0.06 | 0.13 | 0.12 | 0.14 | 0.98 | 0.98 | 0.99 | -103.8 |
| **24** | 0.06 | 0.04 | 0.10 | 0.09 | 0.07 | 0.10 | 0.98 | 0.96 | 0.99 | -109.2 |
| **25** | 0.02 | 5E-4 | 0.04 | 0.14 | 0.13 | 0.15 | 0.98 | 0.96 | 0.99 | -109.0 |
| **25*** | 0 | - | - | 0.15 | 0.14 | 0.15 | 0.98 | 0.97 | 0.99 | -107.44 |
| **26** | 0.52 | 0.40 | 0.66 | 0.09 | 0.04 | 0.11 | 0.98 | 0.97 | 0.99 | -38.03 |
| **27** | 0.45 | 0.34 | 0.62 | 0.13 | 0.09 | 0.16 | 0.98 | 0.96 | 0.99 | -26.98 |
| **28** | 0.14 | -0.05 | 0.38 | 0.11 | 0.09 | 0.20 | 0.95 | 0.79 | 1.00 | -23.80 |
| **28*** | 0 | - | - | 0.14 | 0.13 | 0.17 | 0.97 | 0.96 | 0.99 | -23.01 |
| **29** | 0.22 | 0.11 | 0.35 | 0.09 | 0.06 | 0.11 | 0.95 | 0.80 | 1.00 | -29.74 |
| **30** | 0.18 | 0.11 | 0.25 | 0.05 | 0.04 | 0.08 | 0.96 | 0.86 | 0.99 | -35.77 |
| **31** | 0.35 | 0.28 | 0.45 | 0.04 | 0.03 | 0.06 | 0.95 | 0.89 | 0.98 | -44.25 |
| **32** | 0.33 | 0.26 | 0.45 | 0.05 | 0.03 | 0.06 | 0.94 | 0.89 | 0.97 | -41.95 |
| **33** | -0.27 | -0.34 | -0.21 | 0.49 | 0.45 | 0.54 | 0.99 | 0.97 | 0.99 | -41.90 |
| **34** | 0.29 | -0.02 | 0.69 | 0.22 | 0.15 | 0.43 | 0.88 | 0.80 | 0.94 | -23.99 |
| **34*** | 0 | 0 | 0 | 0.30 | 0.24 | 0.37 | 0.93 | 0.89 | 0.97 | -22.17 |
| **35** | -0.64 | -1.72 | -0.16 | 0.67 | 0.40 | 1.03 | 0.91 | 0.86 | 0.95 | -15.72 |
| **35*** | 0 | - | - | 0.44 | 0.34 | 0.60 | 0.63 | 0.25 | 0.84 | -13.82 |
| **36** | -0.54 | -1.19 | -0.17 | 1.05 | 0.67 | 1.27 | 0.95 | 0.82 | 0.97 | -11.46 |
| **36*** | 0 | - | - | 0.74 | 0.56 | 0.90 | 0.94 | 0.90 | 0.96 | -10.00 |
| **37** | 5E-3 | -0.05 | 0.08 | 0.03 | 0.02 | 0.03 | 0.99 | 0.97 | 1.00 | -31.33 |
| **37*** | 0 | - | - | 0.03 | 0.03 | 0.03 | 0.94 | 0.90 | 0.96 | -33.26 |
| **38** | -0.03 | -0.06 | 0.05 | 0.07 | 0.05 | 0.10 | 0.94 | 0.87 | 0.96 | -42.20 |
| **38*** | 0 | - | - | 0.07 | 0.06 | 0.08 | 0.94 | 0.91 | 0.97 | -43.54 |
| **39** | -0.02 | -0.06 | 0.07 | 0.08 | 0.06 | 0.12 | 0.94 | 0.89 | 0.97 | -43.27 |
| **39*** | 0 | - | - | 7E-3 | 0.07 | 0.08 | 0.94 | 0.90 | 0.96 | -44.54 |
| **40** | -0.02 | -0.07 | 0.08 | 0.09 | 0.06 | 0.14 | 0.96 | 0.90 | 0.98 | -41.04 |
| **40*** | 0 | - | - | 0.08 | 0.08 | 0.10 | 0.94 | 0.90 | 0.96 | -42.81 |

Note: * refers to sets that have been rescaled considering the intercept with a value of zero.

**Second-degree polynomial equation by Bootstrapping adjusted regression**

| **No.** | **a_B_** | **IC_95%_** | | **b_B_** | **IC_95%_** | | **c_B_** | **IC_95%_** | | **R^2^_B_** | **IC_95%_** | | **AIC** |
| --- | --- | --- | --- | --- | --- | --- | --- | --- | --- | --- | --- | --- | --- |
| **1** | 38.86 | 26.42 | 60.59 | 0.88 | 0.20 | 2.21 | -4E-3 | -0.01 | -6E-4 | 0,68 | 0,49 | 0,83 | 51,50 |
| **2** | 40.51 | 28.56 | 57.02 | 0.74 | 0.19 | 2.10 | -3E-3 | -0.01 | -6E-4 | 0,68 | 0,48 | 0,83 | 48,32 |
| **3** | 24.27 | 19.85 | 37.16 | 0.57 | 0.27 | 0.73 | -1E-3 | -3E-3 | -5E-4 | 0,80 | 0,73 | 0,85 | 47,17 |
| **4** | 8.02 | -47.50 | 33.00 | 0.32 | -0.37 | 0.48 | -3E-4 | -9E-4 | 3E-3 | 0,94 | 0,87 | 0,95 | 14,89 |
| **4*** | 0 | - | - | 0.40 | 0.36 | 0.46 | -4E-4 | -8E-4 | -3E-3 | 0,97 | 0,80 | 1 | 16,52 |
| **5** | 7.71 | -116.5 | 18.50 | 0.24 | -0.68 | 0.87 | -2E-4 | -1E-3 | 4E-3 | 0,90 | 0,82 | 0,96 | 17,08 |
| **5*** | 0 | - | - | 0.30 | 0.24 | 0.39 | -3E-4 | -9E-4 | -2E-4 | 0,94 | 0,67 | 1 | 16,73 |
| **6** | 14.69 | 9.79 | 37.57 | 5.21 | 2.62 | 8.27 | -0.07 | -0.16 | -0.03 | 0,85 | 0,76 | 0,90 | 33,52 |
| **6*** | 0 | - | - | 7.85 | 4.99 | 10.21 | -0.12 | -0.25 | -0.06 | 0,91 | 0,74 | 0,91 | 40,17 |
| **7** | 64 | - | - | 36 | - | - | -8 | - | - | - | - | - | - |
| **8** | -26.3 | - | - | 145.9 | - | - | -34.6 | - | - | - | - | - | - |
| **9** | -27.5 | - | - | 129.5 | - | - | -29.0 | - | - | - | - | - | - |
| **10** | 1.33 | 1.33 | 56.00 | 14.54 | 2.66 | 17.92 | -0.45 | -0.90 | -0.05 | 0,81 | 0,69 | 0,86 | 26,12 |
| **10*** | 0 | - | - | 14.24 | 9.66 | 15.85 | -0.44 | -0.79 | 1.16 | 0,99 | 0,61 | 0,99 | 25,93 |
| **11** | 3.00 | -2.10 | 35.00 | 7.83 | 3.16 | 12.69 | -0.19 | -0.79 | 0.46 | 0,90 | 0,83 | 0,95 | 20,59 |
| **11*** | 0 | - | - | 8.98 | 7.56 | 10.74 | -0.24 | -0.43 | 0.15 | 0,99 | 0,78 | 0,99 | 19,53 |
| **12** | 30.41 | 22.51 | 48.47 | 0.98 | 0.43 | 1.48 | -3E-3 | -8E-3 | -8E-4 | 0,88 | 0,76 | 0,95 | 54,25 |
| **12*** | 36.03 | 25.86 | 46.83 | 0.439 | 0.33 | 0.58 | 0 | - | - | 0,88 | 0,76 | 0,95 | 55,86 |
| **13** | 15.62 | 12.78 | 26.04 | 0.37 | 0.17 | 0.45 | -7E-4 | -1E-3 | -2E-4 | 0,84 | 0,73 | 0,92 | 36,89 |
| **14** | 37.81 | 33.73 | 56.22 | 0.72 | 0.30 | 1.07 | -2E-3 | -5E-3 | -7E-4 | 0,78 | 0,67 | 0,86 | 35,05 |
| **15** | 45.07 | 39.50 | 65.62 | 0.74 | 0.22 | 1.04 | -2E-3 | -5E-3 | -5E-4 | 0,73 | 0,62 | 0,81 | 38,32 |
| **15*** | 55.96 | 45.64 | 71.64 | 0.17 | 0.07 | 0.44 | 0 | - | - | 0,73 | 0,62 | 0,81 | 43,82 |
| **16** | 46.40 | 40.84 | 58.96 | 0.55 | 0.24 | 0.84 | -1E-3 | -4E-3 | -5E-4 | 0,81 | 0,73 | 0,87 | 33,01 |
| **16*** | 53.78 | 47.46 | 64.89 | 0.17 | 0.10 | 0.36 | 0 | - | - | 0,81 | 0,69 | 0,86 | 38,89 |
| **17** | 54.38 | 48.55 | 71.55 | 0.52 | 0.12 | 0.84 | -1E-3 | -4E-3 | -2E-4 | 0,74 | 0,63 | 0,82 | 35,13 |
| **17*** | 62.45 | 54.15 | 74.35 | 0.14 | 0.06 | 0.35 | 0 | - | - | 0,74 | 0,62 | 0,80 | 39,87 |
| **18** | -55.91 | -176.31 | 71.99 | 180.43 | 19.00 | 878.73 | -46.16 | -250.83 | -2.72 | 0,62 | 0,37 | 0,76 | 41,42 |
| **19** | -112.31 | -172.59 | 66.83 | 289.6 | 27.05 | 402.89 | -73.37 | -105.11 | -0.02 | 0,68 | 0,46 | 0,96 | 44,98 |
| **19*** | 0 | - | - | 93.74 | -26.81 | 121.4 | -20.45 | -32.1 | 118.84 | 0,55 | 0,30 | 0,84 | 46,33 |
| **20** | 6.23 | 5.64 | 12.86 | 3.97 | 2.73 | 4.56 | -0.10 | -0.17 | -0.06 | 0,88 | 0,82 | 0,96 | 10,63 |
| **21** | 8.53 | 7.84 | 8.71 | 2.39 | 2.28 | 2.57 | -0.05 | -0.06 | -0.04 | 0,94 | 0,90 | 0,96 | -30,87 |
| **22** | 8.57 | 6.45 | 9.18 | 3.10 | 2.74 | 3.63 | -0.06 | -0.10 | -0.04 | 0,92 | 0,87 | 0,98 | 7,98 |
| **23** | 14.42 | 13.19 | 14.99 | 3.93 | 3.66 | 4.32 | -0.08 | -0.12 | -0.06 | 0,93 | 0,88 | 0,96 | -2,83 |
| **24** | 11.92 | 10.25 | 14.85 | 3.25 | 2.54 | 3.85 | -0.08 | -0.12 | -0.04 | 0,90 | 0,87 | 0,92 | 12,90 |
| **25** | 13.71 | 12.37 | 15.23 | 3.95 | 3.62 | 4.30 | -0.08 | -0.11 | -0.05 | 0,95 | 0,93 | 0,96 | 3,08 |
| **26** | 61.69 | 56.39 | 68.24 | 0.55 | 0.09 | 1.16 | -2E-3 | -0.01 | 5E-4 | 0,92 | 0,71 | 0,99 | 27,36 |
| **27** | 52.60 | 41.14 | 71.46 | 1.51 | -0.12 | 4.33 | -0.01 | -0.07 | 0.04 | 0,85 | 0,70 | 0,94 | 26,40 |
| **28** | 23.11 | 17.80 | 80.00 | 2.57 | -0.37 | 4.30 | -0.02 | 0.05 | 0.01 | 0,85 | 0,64 | 0,94 | 27,86 |
| **29** | 28.70 | 15.81 | 46.81 | 1.66 | 0.58 | 4.97 | -0.01 | -0.05 | 1E-3 | 0,86 | 0,78 | 0,94 | 27,44 |
| **30** | 21.98 | 16.00 | 32.83 | 1.30 | -0.22 | 2.03 | -0.01 | -0.02 | 4E-3 | 0,90 | 0,76 | 0,96 | 21,42 |
| **31** | 37.91 | 31.65 | 47.23 | 0.70 | 0.35 | 0.98 | -3E-3 | -7E-3 | -1E-3 | 0,86 | 0,75 | 0,92 | 28,26 |
| **31*** | 43.59 | 35.43 | 50.73 | 0.24 | 0.15 | 0.43 | 0 | - | - | 0,86 | 0,75 | 0,92 | 33,03 |
| **32** | 37.05 | 29.62 | 47.42 | 0.75 | 0.34 | 1.08 | -4E-3 | -7E-3 | -1E-3 | 0,84 | 0,73 | 0,91 | 30,48 |
| **32*** | 42.57 | 35.01 | 51.74 | 0.26 | 0.14 | 0.39 | 0 | - | - | 0,84 | 0,73 | 0,91 | 34,88 |
| **33** | -7.16 | -13.24 | 19.60 | 31.71 | 10.06 | 39.84 | -3.65 | -6.16 | 1.00 | 0,96 | 0,93 | 0,99 | 15,08 |
| **33*** | 0 | - | - | 17.22 | 16.02 | 20.85 | 0 | - | - | 0,93 | 0,86 | 0,98 | 22,81 |
| **34** | 32.12 | 21.47 | 73.92 | 6.44 | 1.11 | 12.33 | -0.10 | -0.29 | 0.36 | 0,66 | 0,49 | 0,77 | 50,34 |
| **34*** | -0.93 | -1.17 | -0.40 | 0.57 | 0.38 | 0.80 | 0 | - | - | 0,92 | 0,83 | 0,98 | -20,17 |
| **35** | 21.94 | 18.64 | 29.05 | 8.76 | 6.95 | 10.65 | -0.24 | -0.42 | -0.15 | 0,97 | 0,94 | 0,98 | 14,90 |
| **36** | 20.81 | 11.11 | 77.00 | 23.58 | 7.64 | 49.65 | -1.77 | -6.80 | -0.15 | 0,90 | 0,72 | 0,97 | 28,12 |
| **36*** | 0 | - | - | 38.42 | 27.83 | 50.28 | -3.41 | -5.61 | -1.91 | 0,91 | 0,56 | 0,97 | 33,16 |
| **37** | 11.29 | 6.33 | 14.83 | 0.22 | 0.07 | 0.32 | -3E-4 | -7E-4 | 7E-4 | 0,99 | 0,98 | 1,00 | 4,03 |
| **37*** | 0 | - | - | 0.51 | 0.41 | 1 | -1E-3 | -4E-3 | -1E-3 | 0,92 | 0,82 | 1,00 | 13,43 |
| **38** | -0.66 | -4.24 | 8.27 | 4.00 | 1.56 | 7.13 | -0.11 | -0.31 | 0.14 | 0,85 | 0,79 | 0,90 | 19,45 |
| **38*** | 0 | - | - | 4.01 | 0.89 | 4.90 | -0.11 | -0.17 | 2.00 | 0,94 | 0,81 | 0,97 | 17,63 |
| **39** | 0.04 | -2.10 | 13.97 | 4.51 | 1.41 | 8.28 | -0.13 | -0.36 | 0.60 | 0,88 | 0,81 | 0,92 | 22,82 |
| **39*** | 0 | - | - | 1.81 | 1.48 | 3.06 | 0 | - | - | 0,80 | 0,59 | 0,85 | 30,89 |
| **40** | 0.89 | -1.35 | 16.23 | 5.15 | 1.08 | 8.24 | -0.15 | -0.38 | -0.02 | 0,87 | 0,79 | 0,91 | 24,29 |
| **40*** | 0 | - | - | 2.01 | 1.60 | 3.40 | 0 | - | - | 0,77 | 0,41 | 0,86 | 33,10 |

Note: * refers to sets that have been rescaled considering the intercept with a value of zero.

**Third-degree polynomial equation by Bootstrapping adjusted regression.**

| **No.** | **a_B_** | **IC_95%_** | | **b_B_** | **IC_95%_** | | **c_B_** | **IC_95%_** | | **d_B_** | **IC_95%_** | | **R^2^_B_** | **IC_95%_** | | **AIC** |
| --- | --- | --- | --- | --- | --- | --- | --- | --- | --- | --- | --- | --- | --- | --- | --- | --- |
| **1** | 26.55 | 5.82 | 49.82 | 3.14 | 1.04 | 13.71 | -0.05 | -0.86 | -0.01 | 2E-4 | 3e-5 | 7E-3 | 0.88 | 0.66 | 1.00 | 49.26 |
| **2** | 30.23 | 14.44 | 46.16 | 2.85 | 0.99 | 10.01 | -0.04 | -0.49 | -0.01 | 1E-4 | 3e-5 | 4E-3 | 0.89 | 0.67 | 1.00 | 45.61 |
| **3** | 20.02 | 15.22 | 30.74 | 1.08 | 0.56 | 2.09 | -9e-3 | -0.03 | -3E-3 | 2e-5 | 7e-6 | 2e-4 | 0.95 | 0.89 | 0.99 | 41.68 |
| **4** | 15.13 | -0.01 | 0.71 | 0.13 | -1.76 | 2.50 | 5e-4 | -9E-3 | 0.01 | -1e-6 | -4e-5 | 1e-5 | 1.00 | 0.94 | 1.00 | 14.89 |
| **4*** | 0 | - | - | 0.52 | -0.34 | 0.81 | -1E-3 | -8E-3 | 8E-3 | 1E-6 | -2E-5 | 3E-5 | 1.00 | 0.90 | 1.00 | 15.91 |
| **5** | 21.75 | -0.01 | 86.00 | -0.01 | -2.30 | 2.55 | 1E-3 | -9E-3 | 0.02 | -2e-5 | -5e-5 | 1e-5 | 1.00 | 0.86 | 1.00 | 16.50 |
| **5*** | 0 | - | - | 0.41 | -0.59 | 0.80 | -1E-3 | -0.01 | 0.01 | 1.31 | -3E-5 | 4E-5 | 1 | 0.80 | 1 | 18.36 |
| **6** | 6.04 | 0.83 | 28.66 | 11.99 | 4.24 | 19.53 | -0.66 | -2.48 | -0.10 | 9E-3 | 9e-4 | 0.10 | 0.99 | 0.95 | 1.00 | 28.07 |
| **6*** | 0 | - | - | 4.77 | 3.90 | 12.18 | 0 | - | - | 0 | - | - | 0.72 | 0.55 | 0.98 | 35.17 |
| **7** | 64 | - | - | 36 | - | - | -8 | - | - | - | - | - | - | - | - | - |
| **8** | -26.3 | - | - | 145.9 | - | - | -34.6 | - | - | - | - | - | - | - | - | - |
| **9** | -27.5 | - | - | 129.5 | - | - | -29.0 | - | - | - | - | - | - | - | - | - |
| **10** | -7.96 | -8.19 | -6.47 | 21.57 | 20.88 | 21.89 | -1.64 | -1.74 | -1.57 | 0.03 | 0.03 | 0.04 | 1.00 | 0.99 | 1.00 | -12.11 |
| **11** | 2.23 | -25.95 | 6.46 | 8.02 | 2.03 | 20.94 | -0.26 | -1.53 | 1.61 | 2E-3 | -0.11 | 0.03 | 1.00 | 0.96 | 1.00 | 17.26 |
| **11*** | 0 | - | - | 10.25 | 5.80 | 13.37 | -0.39 | -1.97 | 0.98 | 6E-3 | -0.08 | 0.24 | 1.00 | 0.93 | 1.00 | 16.45 |
| **12** | 24.45 | 8.84 | 39.70 | 2.06 | 0.98 | 10.91 | -0.02 | -0.39 | -6E-3 | 8E-5 | 1e-5 | 3E-3 | 0.89 | 0.77 | 0.99 | 52.37 |
| **12*** | 36.03 | 25.86 | 46.83 | 0.439 | 0.33 | 0.58 | 0 | - | - | 0 | - | - | 0.88 | 0.76 | 0.95 | 55.86 |
| **13** | 11.41 | 9.01 | 0.14 | 0.59 | 0.48 | 0.75 | -2E-3 | -4E-3 | -1E-3 | 3e-6 | 1e-6 | 7e-6 | 0.98 | 0.96 | 0.99 | 23.08 |
| **14** | 31.10 | 23.52 | 46.33 | 1.45 | 0.70 | 3.36 | -0.01 | -0.08 | -3E-3 | 3e-5 | 6e-6 | 5e-4 | 0.98 | 0.92 | 1.00 | 28.20 |
| **14*** | 37.81 | 33.73 | 56.22 | 0.72 | 0.30 | 1.07 | -2E-3 | -5E-3 | -7E-4 | 0 | - | - | 0.78 | 0.67 | 0.86 | 35.05 |
| **15** | 36.19 | 27.58 | 57.75 | 1.85 | 0.60 | 4.05 | -0.02 | -0.09 | -3E-3 | 5e-5 | 8e-6 | 5e-4 | 0.96 | 0.85 | 1.00 | 33.03 |
| **15*** | 55.96 | 45.64 | 71.64 | 0.17 | 0.07 | 0.44 | 0 | - | - | 0 | - | - | 0.73 | 0.62 | 0.81 | 43.82 |
| **16** | 40.58 | 30.73 | 57.42 | 1.15 | 0.35 | 3.92 | -0.01 | -0.12 | -1E-3 | 2e-5 | 2e-6 | 8e-4 | 0.97 | 0.85 | 1.00 | 29.75 |
| **16*** | 53.78 | 47.46 | 64.89 | 0.17 | 0.10 | 0.36 | 0 | - | - | 0 | - | - | 0.81 | 0.69 | 0.86 | 38.89 |
| **17** | 47.05 | 38.35 | 65.87 | 1.59 | 0.35 | 3.72 | -1E-3 | -0.10 | -2E-3 | 6e-5 | 4e-6 | 6e-4 | 0.96 | 0.80 | 1.00 | 32.44 |
| **18** | -194.3 | -1847.0 | 74.13 | 562.06 | -77.11 | 7447.3 | -295.7 | -9470 | 135.68 | 47.03 | -35.70 | 3966.6 | 1.00 | 0.71 | 1.00 | 38.31 |
| **18*** | 0 | - | - | 86.96 | -1172 | 164.3 | -14.47 | -90.84 | 3459.0 | -0.59 | -2190 | 15.78 | 0.78 | 0.37 | 1.00 | 42.18 |
| **19** | -230.0 | -321.61 | 418.0 | 611.34 | -2446 | 1012.0 | -316.4 | -850.0 | 4400 | 50.45 | -2333 | 171.77 | 0.97 | 0.91 | 0.98 | 28.38 |
| **20** | 4.78 | 3.19 | 5.73 | 5.22 | 4.38 | 6.48 | -0.27 | -0.53 | -0.12 | 5E-3 | -2E-3 | 0.01 | 0.99 | 0.98 | 0.99 | -1.90 |
| **21** | 8.87 | 7.86 | 8.99 | 2.11 | 1.98 | 2.58 | -0.01 | -0.10 | 0.02 | -1E-3 | -3E-3 | 5E-3 | 0.99 | 0.99 | 0.99 | -35.98 |
| **21*** | 8.99 | 8.90 | 9.48 | 2.02 | 1.92 | 2.07 | 0 | - | - | -1E-3 | -2E-3 | -2E-4 | 0.99 | 0.99 | 0.99 | -34.67 |
| **22** | 9.58 | 7.50 | 10.84 | 2.26 | 1.50 | 5.24 | 0.04 | -0.72 | 0.21 | -3E-3 | -0.01 | 0.04 | 0.99 | 0.96 | 0.99 | 6.92 |
| **22*** | 13.06 | 9.80 | 18.71 | 1.53 | 1.19 | 2.42 | 0 | - | - | 0 | - | - | 0.92 | 0.88 | 0.98 | 40.25 |
| **23** | 14.81 | 14.10 | 17.82 | 3.54 | 2.08 | 4.32 | -0.02 | -0.21 | 0.15 | -1E-3 | -0.01 | 7E-3 | 0.99 | 0.99 | 0.99 | -4.61 |
| **23*** | 20.36 | 15.91 | 25.80 | 1.92 | 1.51 | 3.02 | 0 | - | - | 0 | - | - | 0.93 | 0.89 | 0.95 | 45.66 |
| **24** | 10.36 | 8.84 | 16.08 | 4.30 | 1.36 | 5.61 | -0.22 | -0.47 | 0.22 | 4E-3 | -0.01 | 0.01 | 0.98 | 0.95 | 0.98 | 10.75 |
| **24*** | 11.92 | 10.25 | 14.85 | 3.25 | 2.54 | 3.85 | -0.08 | -0.12 | -0.04 | 0 | - | - | 0.90 | 0.87 | 0.92 | 12.90 |
| **25** | 13.43 | 12.31 | 18.27 | 4.04 | 1.51 | 4.81 | -0.08 | -0.22 | 0.27 | 4e-5 | -0.01 | 7E-3 | 0.99 | 0.98 | 0.99 | 4.98 |
| **25*** | 18.98 | 15.07 | 24.51 | 2.12 | 1.68 | 3.10 | 0 | - | - | 0 | - | - | 0.95 | 0.93 | 0.96 | 43.41 |
| **26** | 55.78 | 47.42 | 63.78 | 1.56 | 0.38 | 3.59 | -0.04 | -0.11 | 0.07 | 2E-4 | -3E-3 | 1E-3 | 0.98 | 0.85 | 0.92 | 21.98 |
| **26*** | 64.69 | 59.86 | 67.46 | 0.21 | 0.17 | 0.37 | 0 | - | - | 0 | - | - | 0.92 | 0.72 | 0.99 | 27.36 |
| **27** | 40.49 | 44.01 | 64.32 | 5.05 | 1.30 | 5.65 | -0.13 | -0.23 | 0.21 | 1E-3 | -0.01 | 2E-3 | 0.88 | 0.84 | 0.88 | 21.39 |
| **28** | 14.02 | 11.81 | 35.55 | 4.72 | 0.68 | 5.89 | -0.11 | -0.19 | 0.07 | 8E-4 | -1E-3 | 7E-3 | 0.99 | 0.98 | 0.99 | 10.53 |
| **29** | 14.33 | 12.78 | 38.17 | 5.73 | 1.75 | 6.40 | -0.16 | -0.23 | -0.03 | 1E-3 | 2E-4 | 2E-3 | 0.89 | 0.89 | 0.89 | 22.89 |
| **29*** | 0 | - | - | 9.34 | 4.66 | 9.88 | -0.36 | -0.41 | -0.10 | 3E-3 | 7E-4 | 4E-3 | 0.95 | 0.66 | 0.92 | 32.93 |
| **30** | 15.22 | 14.49 | 21.75 | 3.12 | 2.00 | 4.62 | -0.07 | -0.25 | -0.04 | 6E-4 | 3E-4 | 1E-3 | 0.98 | 0.98 | 0.98 | 8.63 |
| **30*** | 28.33 | 22.37 | 35.89 | 0.43 | 0.29 | 0.87 | 0 | - | - | 0 | - | - | 0.90 | 0.77 | 0.97 | 24.04 |
| **31** | 33.76 | 28.87 | 46.28 | 1.33 | 0.40 | 1.93 | -0.01 | -0.03 | -2E-3 | 8e-5 | 1e-6 | 2E-4 | 0.91 | 0.77 | 0.92 | 26.09 |
| **31*** | 43.59 | 35.43 | 50.73 | 0.24 | 0.15 | 0.43 | 0 | - | - | 0 | - | - | 0.86 | 0.75 | 0.92 | 33.03 |
| **32** | 32.20 | 22.88 | 45.82 | 1.50 | 0.52 | 3.50 | -0.02 | -0.08 | -4E-3 | 9e-5 | 1e-5 | 5E-4 | 0.95 | 0.76 | 0.91 | 27.70 |
| **32*** | 42.57 | 35.01 | 51.74 | 0.26 | 0.14 | 0.39 | 0 | - | - | 0 | - | - | 0.84 | 0.73 | 0.91 | 34.88 |
| **33** | -13.30 | -56.40 | 16.00 | 49.06 | -44.33 | 112.53 | -14.60 | -42.60 | 66.00 | 1.73 | -18.66 | 5.46 | 0.97 | 0.97 | 0.97 | 13.63 |
| **33*** | 0 | - | - | 15.83 | 10.09 | 36.50 | 4.80 | -10.25 | 13.31 | -1.32 | -3.93 | 2.66 | 0.99 | 0.94 | 0.97 | 17.67 |
| **34** | 10.75 | 9.68 | 107.18 | 15.82 | -18.73 | 19.71 | -0.74 | -1.57 | 3.87 | 9E-3 | -0.16 | 0.03 | 0.98 | 0.66 | 0.93 | 39.96 |
| **34*** | 0 | - | - | 21.13 | 12.71 | 20.83 | -1.40 | -1.39 | -0.29 | 0.02 | -0.04 | 0.02 | 0.97 | 0.56 | 0.95 | 46.08 |
| **35** | 17.97 | 15.04 | 63.48 | 11.71 | -3.51 | 14.58 | -0.72 | -1.29 | 1.27 | 0.01 | -0.05 | 0.05 | 0.99 | 0.98 | 0.99 | 13.90 |
| **35*** | 32.42 | 25.64 | 44.34 | 4.69 | 3.42 | 5.83 | 0 | - | - | 0 | - | - | 0.97 | 0.94 | 0.98 | 31.63 |
| **36** | 11.78 | -30.20 | 24.74 | 40.72 | -2.83 | 95.76 | -7.03 | -20.45 | 14.53 | 0.41 | -2.24 | 1.59 | 0.92 | 0.83 | 0.96 | 26.44 |
| **36*** | 0 | - | - | 56.17 | 31.33 | 68.08 | -11.04 | -18.98 | -2.93 | 0.70 | -0.48 | 4.23 | 0.98 | 0.63 | 0.97 | 26.31 |
| **37** | 16.25 | - | - | -0.02 | - | - | 2E-3 | - | - | -1e-5 | - | - | NA | NA | NA | - |
| **38** | -4.54 | -6.57 | 12.28 | 8.50 | -41.00 | 12.82 | -0.68 | -2.05 | 38.00 | 0.01 | -8.00 | 0.10 | 0.98 | 0.89 | 0.94 | 17.72 |
| **39** | -5.48 | -5.83 | 13.19 | 10.75 | -12.90 | 12.26 | -0.94 | -1.76 | 16.80 | 0.02 | -3.60 | 0.08 | 0.96 | 0.90 | 0.97 | 15.40 |
| **39*** | 0 | - | - | 1.81 | 1.48 | 3.06 | 0 | - | - | 0 | - | - | 0.80 | 0.59 | 0.85 | 30.89 |
| **40** | -4.48 | -6.05 | 12.20 | 10.91 | -39.73 | 13.77 | -1.00 | -1.85 | 38.80 | 0.02 | -8.26 | 0.08 | 0.99 | 0.95 | 1.00 | 16.00 |
| **40*** | 0 | - | - | 2.01 | 1.60 | 3.40 | 0 | - | - | 0 | - | - | 0.77 | 0.41 | 0.86 | 33.10 |

Note: * refers to sets that have been rescaled considering the intercept with a value of zero.

**Fourth-degree polynomial equation by Bootstrapping adjusted regression.**

| **No.** | **a_B_** | **IC_95%_** | | **b_B_** | **IC_95%_** | | **c_B_** | **IC_95%_** | | **d_B_** | **IC_95%_** | | **e_B_** | **IC_95%_** | | **R^2^_B_** | **IC_95%_** | | **AIC** |
| --- | --- | --- | --- | --- | --- | --- | --- | --- | --- | --- | --- | --- | --- | --- | --- | --- | --- | --- | --- |
| **1** | 13.38 | 0.20 | 39.96 | 8.41 | 2.71 | 1.99 | -0.36 | -2.20 | -0.05 | 4E-3 | 4e-4 | 0.08 | -1e-5 | -5e-4 | -1e-6 | 0.98 | 0.85 | 1.00 | 44.81 |
| **1*** | 47.23 | 31.85 | 60.52 | 0.24 | 0.12 | 0.49 | 0 | - | - | 0 | - | - | 0 | - | - | 0.68 | 0.49 | 0.83 | 52.37 |
| **2** | 20.22 | -1.50 | 39.73 | 6.51 | 2.17 | 25.45 | -0.26 | -3.23 | -0.04 | 3E-3 | 3E-4 | 0.14 | -1e-5 | -1E-3 | -9e-7 | 0.98 | 0.86 | 1.00 | 40.67 |
| **2*** | 46.93 | 35.90 | 58.16 | 0.21 | 0.09 | 0.36 | 0 | - | - | 0 | - | - | 0 | - | - | 0.68 | 0.44 | 0.83 | 49.29 |
| **3** | 16.4 | 12.47 | 27.69 | 1.99 | 0.96 | 3.69 | -0.03 | -0.14 | -0.01 | 3e-4 | 9e-5 | 2E-3 | -7e-7 | -9e-6 | -2e-7 | 0.97 | 0.95 | 0.99 | 34.76 |
| **4** | 94.14 | - | - | -2.68 | - | - | 0.03 | - | - | -1e-4 | - | - | 1e-7 | - | - | NA | NA | NA | - |
| **5** | 111.7 | - | - | -3.35 | - | - | 0.03 | - | - | -1e-4 | - | - | 1E-7 | - | - | NA | NA | NA | - |
| **6** | 0.85 | 0.25 | 7.12 | 19.49 | 12.00 | 20.76 | -2.45 | -3.07 | -0.92 | 0.10 | 0.03 | 0.20 | -1E-3 | -4E-3 | -1E-4 | 1.00 | 0.99 | 1.00 | 15.96 |
| **6*** | 0 | - | - |  |  |  | 0 | - | - | 0 | - | - | 0 | - | - | NA | NA | NA | - |
| **7** | 64 | - | - | 36 | - | - | -8 | - | - | - | - | - | - | - | - | NA | NA | NA | - |
| **8** | -26.3 | - | - | 145.9 | - | - | -34.6 | - | - | - | - | - | - | - | - | NA | NA | NA | - |
| **9** | -27.5 | - | - | 129.5 | - | - | -29.0 | - | - | - | - | - | - | - | - | NA | NA | NA | - |
| **10** | -8.26 | - | - | 22.00 | - | - | -1.79 | - | - | 0.05 | - | - | -3E-4 | - | - | NA | NA | NA | - |
| **11** | 7.87 | - | - | -0.19 | - | - | 2.57 | - | - | -0.25 | - | - | 6E-3 | - | - | NA | NA | NA | - |
| **12** | 15.43 | 2.09 | 3.67 | 7.38 | 0.25 | 23.53 | -0.17 | -2.86 | 0.15 | 1E-3 | -0.01 | 0.07 | -3e-6 | -4e-4 | 9e-5 | 0.95 | 0.80 | 1.00 | 53.42 |
| **13** | 10.53 | 7.49 | 17.50 | 0.66 | 0.20 | 1.13 | -3E-3 | -9E-3 | 4E-3 | 8e-6 | -3e-5 | 3e-5 | -6e-9 | -6e-8 | 7e-8 | 0.98 | 0.96 | 0.99 | 22.82 |
| **13*** | 26.05 | 20.02 | 36.72 | 0.12 | 0.07 | 0.17 | 0 | - | - | 0 | - | - | 0 | - | - | 0.84 | 0.70 | 0.92 | 51.58 |
| **14** | 26.87 | 17.99 | 36.87 | 2.64 | -0.56 | 4.44 | -0.05 | -0.13 | 0.30 | 4e-4 | -0.01 | 2E-3 | -9e-7 | -8e-6 | 5e-5 | 1.00 | 0.95 | 1.00 | 24.41 |
| **14*** | 48.67 | 40.40 | 63.45 | 0.19 | 0.10 | 0.46 | 0 | - | - | 0 | - | - | 0 | - | - | 0.78 | 0.65 | 0.85 | 43.26 |
| **15** | 31.29 | 19.33 | 57.03 | 2.85 | 0.64 | 5.94 | -0.06 | -5E-3 | 3.34 | 5e-4 | -5E-3 | 3E-3 | -1e-6 | -1e-5 | 3e-5 | 1.00 | 0.92 | 1.00 | 27.84 |
| **15*** | 55.96 | 45.64 | 71.64 | 0.17 | 0.07 | 0.44 | 0 | - | - | 0 | - | - | 0 | - | - | 0.73 | 0.62 | 0.81 | 43.82 |
| **16** | 33.14 | 27.48 | 60.77 | 3.03 | 6E-3 | 5.14 | -0.06 | -0.23 | 0.01 | 6e-4 | -1e-4 | 3E-3 | -13-6 | -1e-5 | 2e-6 | 1.00 | 0.90 | 1.00 | 28.45 |
| **17** | 40.46 | 37.02 | 57.25 | 3.12 | 1.21 | 4.41 | -0.07 | -0.17 | -0.02 | 7e-4 | 1e-4 | 3E-3 | -2e-6 | -1e-5 | -3e-7 | 1.00 | 0.94 | 1.00 | 22.35 |
| **18** | -983.28 | -2329 | 117 | 3480 | -262 | 10299 | -3724 | -15657 | 411 | 1537 | -199 | 9792 | -214 | -2008 | 30 | 1.00 | 0.73 | 1.00 | 35.83 |
| **18*** | 0 | - | - | -282.8 | -3480 | 245.8 | 790.72 | -362.5 | 14233 | 790.7 | -18351 | 281.5 | 72.63 | -67.85 | 7695.8 | 1.00 | 0.53 | 1.00 | 40.35 |
| **19** | -214 | -539 | 631 | 595 | -3799 | 1700 | -364 | -1585 | 7585 | 88 | -5632 | 601 | 20 | -79 | 1269 | 1.00 | 0.99 | 1.00 | 22.12 |
| **20** | 5.09 | 1.38 | 6.68 | 4.88 | 2.17 | 8.40 | -0.19 | -1.09 | 0.88 | -1E-3 | -0.15 | 0.08 | 1e-4 | -2E-3 | 7E-3 | 0.99 | 0.98 | 0.99 | -0.15 |
| **20*** | 13.22 | 7.89 | 17.61 | 1.56 | 1.17 | 2.93 | 0 | - | - | 0 | - | - | 0 | - | - | 0.79 | 0.72 | 0.97 | 48.86 |
| **21** | 9.04 | 8.50 | 9.55 | 1.90 | 1.26 | 2.60 | 0.04 | -0.19 | 0.29 | -6E-3 | -0.04 | 0.02 | 1e-4 | -1E-3 | 2E-3 | 0.99 | 0.99 | 0.99 | -36.53 |
| **21*** | 11.97 | 9.49 | 15.64 | 1.21 | 0.95 | 1.86 | 0 | - | - | 0 | - | - | 0 | - | - | 0.94 | 0.91 | 0.97 | 30.67 |
| **22** | 8.71 | 6.05 | 13.18 | 3.42 | -0.79 | 8.00 | -0.29 | -2.15 | 0.75 | 0.02 | -0.06 | 0.29 | -8e-4 | -0.01 | 1E-3 | 0.99 | 0.98 | 0.99 | 8.37 |
| **23** | 14.11 | 12.39 | 18.21 | 4.43 | 1.50 | 7.27 | -0.28 | -1.28 | 0.37 | 0.02 | -0.03 | 0.15 | -5e-4 | -6E-3 | 1E-3 | 0.99 | 0.99 | 0.99 | -3.81 |
| **24** | 8.54 | 5.00 | 16.35 | 6.63 | 1.25 | 12.88 | -0.87 | -3.36 | 0.26 | 0.06 | -0.02 | 0.41 | -1E-3 | -0.01 | 7e-4 | 0.99 | 0.97 | 0.99 | 9.97 |
| **24*** | 17.18 | 13.56 | 23.07 | 1.39 | 1.00 | 2.37 | 0 | - | - | 0 | - | - | 0 | - | - | 0.90 | 0.86 | 0.93 | 42.30 |
| **25** | 12.12 | 8.82 | 18.46 | 5.88 | 1.41 | 10.19 | -0.57 | -2.21 | 0.39 | 0.04 | -0.03 | 0.25 | -1E-3 | -0.01 | 6e-4 | 0.99 | 0.99 | 0.99 | 4.76 |
| **25*** | 18.98 | 15.07 | 24.51 | 2.12 | 1.68 | 3.10 | 0 | - | - | 0 | - | - | 0 | - | - | 0.95 | 0.93 | 0.96 | 43.41 |
| **26** | 49.10 | 35.25 | 63.26 | 4.14 | 0.32 | 14.30 | -0.25 | -2.21 | 0.06 | 5E-3 | -3E-3 | 0.01 | -2e-5 | -2E-3 | 3e-5 | 0.99 | 0.96 | 1.00 | 14.05 |
| **26*** | 64.69 | 59.86 | 67.46 | 0.21 | 0.17 | 0.37 | 0 | - | - | 0 | - | - | 0 | - | - | 0.92 | 0.72 | 0.99 | 27.36 |
| **27** | 39.89 | 23.49 | 44.75 | 5.53 | 1.99 | 9.52 | -0.25 | -0.48 | 0.35 | 4E-3 | -0.02 | 9E-3 | -2e-5 | -6e-5 | 3e-4 | 1.00 | 0.98 | 1.00 | 5.42 |
| **28** | 11.04 | 3.21 | 37.46 | 6.42 | 6E-3 | 12.13 | -0.23 | -1.21 | 0.15 | 3E-3 | -4E-3 | 0.04 | -1e-5 | -5e-4 | 3e-5 | 1.00 | 0.98 | 1.00 | 11.14 |
| **28*** | 0 | - | - | 13.89 | 7.55 | 14.81 | -1.34 | -1.85 | -0.27 | 0.04 | 4E-3 | 0.10 | 4E-4 | -1E-3 | -2E-5 | 1.00 | 0.94 | 1.00 | 22.83 |
| **29** | 11.16 | 7.94 | 21.99 | 7.64 | 5.00 | 9.98 | -0.37 | -0.77 | -0.21 | 7E-3 | 3E-3 | 0.02 | -4e-5 | -2e-4 | -2e-5 | 1.00 | 0.99 | 1.00 | 0.44 |
| **29*** | 0 | - | - | 14.47 | 9.43 | 16.60 | 1.30 | -2.36 | -0.46 | 0.03 | 9E-3 | 0.16 | -3E-4 | -3E-3 | -5E-5 | 1.00 | 0.93 | 1.00 | 21.04 |
| **30** | 13.86 | 11.89 | 20.51 | 3.87 | 2.25 | 5.30 | -0.15 | -0.40 | -0.06 | 2E-3 | 6e-4 | 0.01 | -1e-5 | -1e-4 | -1e-6 | 1.00 | 0.99 | 1.00 | -5.40 |
| **31** | 28.79 | 17.04 | 44.82 | 2.97 | 0.71 | 8.84 | -0.08 | -0.7 | -7E-3 | 9e-4 | -1e-5 | 0.01 | -3e-6 | -1e-4 | 3e-7 | 1.00 | 0.88 | 1.00 | 26.09 |
| **32** | 26.41 | 13.85 | 46.04 | .94 | 0.48 | 9.46 | -0.08 | -0.72 | -2E-3 | 9e-4 | -2e-5 | 0.01 | -3e-6 | -7e-5 | 3e-7 | 1.00 | 0.88 | 1.00 | 27.35 |
| **33** | 5.31 | -78.80 | 31.60 | -8.57 | -109.3 | 166.6 | 34.33 | -88.33 | 157.0 | -13.71 | -70.66 | 21.33 | 1.64 | -1.86 | 10.40 | 1.00 | 1.00 | 1.00 | 9.93 |
| **33*** | 0 | - | - | 5.19 | -30.33 | 28.76 | 25.52 | -27.33 | 86.77 | -11.50 | -44.33 | 34.66 | 1.41 | -10.66 | 6.88 | 1.00 | 0.98 | 1.00 | 8.10 |
| **34** | 10.14 | -28.95 | 66.69 | 16.37 | -29.26 | 53.68 | -0.79 | -16.47 | 12.11 | 6E-3 | -1.29 | 2.24 | 1e-5 | -0.06 | 0.03 | 0.99 | 0.95 | 1.00 | 29.19 |
| **34*** | 0 | - | - | 24.07 | 17.81 | 37.90 | -2.21 | -14.88 | -0.01 | 0.07 | -0.16 | 2.84 | -8E-4 | -0.14 | 4E-3 | 0.98 | 0.92 | 1.00 | 28.11 |
| **35** | 12.70 | -7.00 | 32.93 | 17.72 | -0.01 | 36.71 | -2.35 | -6.68 | 2.20 | 0.18 | -0.29 | 0.66 | -5E-3 | -0.02 | 9E-3 | 1.00 | 0.99 | 1.00 | 6.42 |
| **36** | 15.30 | -48.51 | 31.26 | 32.47 | -22.04 | 130.86 | -6.61 | -47.16 | 45.90 | 0.87 | -14.41 | 7.19 | -0.04 | -0.38 | 1.28 | 1.00 | 0.98 | 1.00 | 24.91 |
| **36*** | 0 | - | - | 24.22 | 19.61 | 34.75 | -2.24 | -10.13 | -0.92 | 0.07 | -0.02 | 1.48 | -8E-4 | -0.05 | 1E-3 | 0.98 | 0.94 | 1.00 | 28.11 |
| **37** | 16.25 | - | - | -0.02 | - | - | 2E-3 | - | - | -1e-5 | - | - | - | - | - | NA | NA | NA | - |
| **38** | -5.43 | -30.10 | 13.66 | 11.05 | -47.67 | 43.42 | -1.66 | -13.87 | 46.70 | 0.12 | -12.44 | 1.60 | -2E-3 | -0.04 | 0.74 | 1.00 | 0.95 | 1.00 | 12.76 |
| **38*** | 0 | - | - | -0.01 | -18.04 | 7.80 | 2.36 | -15.06 | 25.42 | -0.35 | -6.61 | 13.52 | 9E-3 | -1.84 | 0.48 | 0.99 | 0.91 | 1.00 | 18.77 |
| **39** | -5.45 | -15.67 | 3.44 | 11.35 | -15.89 | 23.91 | -1.13 | -5.79 | 20.71 | 0.02 | -5.59 | 0.56 | -1e-4 | -0.01 | 0.33 | 1.00 | 0.99 | 1.00 | 4.60 |
| **40** | -5.26 | -31.95 | 13.93 | 12.35 | -46.65 | 48.81 | -1.79 | -15.63 | 47.83 | 0.11 | -12.88 | 1.83 | -2E-3 | -0.05 | 0.76 | 1.00 | 0.96 | 1.00 | 13.20 |
| **40*** | 0 | - | - | 2.23 | -16.42 | 10.08 | 2.13 | -12.14 | 26.07 | -0.29 | -7.75 | 10.33 | 8E-3 | -0.77 | 0.66 | 0.99 | 0.94 | 1.00 | 17.96 |

Note: * refers to sets that have been rescaled considering the intercept with a value of zero.

**Fifth-degree polynomial equation by Bootstrapping adjusted regression**

| **No.** | **a_B_** | **IC_95%_** | | **b_B_** | **IC_95%_** | | **c_B_** | **IC_95%_** | | **d_B_** | **IC_95%_** | | **e_B_** | **IC_95%_** | | **e_B_** | **IC_95%_** | | **R^2^_B_** | **IC_95%_** | | **AIC** |
| --- | --- | --- | --- | --- | --- | --- | --- | --- | --- | --- | --- | --- | --- | --- | --- | --- | --- | --- | --- | --- | --- | --- |
| **1** | 2.12 | -0.66 | 22.23 | 17.6 | 7.17 | 21.20 | -1.63 | -2.70 | -0.32 | 0.05 | 5E-3 | 0.15 | -6e-4 | -3e-3 | -4e-5 | 2e-6 | 1e-7 | 2e-5 | 1.00 | 0.96 | 1.00 | 34.10 |
| **1*** | 0 | - | - | 19.52 | 11.05 | 20.69 | -1.98 | -2.59 | -0.53 | 0.06 | 9E-3 | 0.14 | -8E-4 | -3E-3 | -6E-5 | 3E-6 | 1E-7 | 2E-5 | 0.99 | 0.80 | 1.00 | 43.16 |
| **2** | 10.21 | -8.70 | 39.59 | 13.99 | 2.69 | 37.50 | -1.33 | -8.55 | -0.09 | 0.04 | 1E-3 | 0.78 | -5e-4 | -0.02 | -8e-6 | 2e-6 | 1e-8 | 1e-4 | 1.00 | 0.93 | 1.00 | 34.25 |
| **3** | 13.37 | 9.91 | 23.81 | 3.39 | 1.48 | 5.61 | -0.12 | -0.33 | -0.03 | 1E-3 | 3e-4 | 9e-3 | -1e-5 | -1e-4 | -2e-6 | 3e-8 | 3e-9 | 3e-7 | 0.99 | 0.97 | 1.00 | 27.05 |
| **3*** | 16.4 | 12.47 | 27.69 | 1.99 | 0.96 | 3.69 | -0.03 | -0.14 | -0.01 | 3e-4 | 9e-5 | 2E-3 | -7e-7 | -9e-6 | -2e-7 | 0 | - | - | 0.97 | 0.95 | 0.99 | 34.76 |
| **4** | 94.14 | - | - | -2.68 | - | - | 0.03 | - | - | -1e-4 | - | - | 1e-7 | - | - | - | - | - | NA | NA | NA | - |
| **5** | 111.7 | - | - | -3.35 | - | - | 0.03 | - | - | -1e-4 | - | - | 1e-7 | - | - | - | - | - | NA | NA | NA | - |
| **6** | 0.75 | -0.57 | 0.85 | 19.8 | 19.47 | 21.79 | -2.77 | -3.48 | -2.41 | 0.19 | 0.08 | 0.26 | -0.05 | -8E-3 | 4E-3 | 5e-5 | -1e-4 | 8e-5 | 1.00 | 0.99 | 1.00 | -20.2 |
| **6*** | 0 | - | - | 4.77 | 3.90 | 12.18 | 0 | - | - | 0 | - | - | 0 | - | - | 0 | - | - | 0.72 | 0.55 | 0.98 | 35.17 |
| **7** | 64 | - | - | 36 | - | - | -8 | - | - | - | - | - | - | - | - | - | - | - | NA | NA | NA | - |
| **8** | -26.3 | - | - | 145.9 | - | - | -34.6 | - | - | - | - | - | - | - | - | - | - | - | NA | NA | NA | - |
| **9** | -27.5 | - | - | 129.5 | - | - | -29 | - | - | - | - | - | - | - | - | - | - | - | NA | NA | NA | - |
| **10** | -8.26 | - | - | 22.00 | - | - | -1.79 | - | - | 0.05 | - | - | -3e-4 | - | - | - | - | - | NA | NA | NA | - |
| **11** | 7.87 | - | - | -0.19 | - | - | 2.57 | - | - | -0.25 | - | - | 6E-3 | - | - | - | - | - | NA | NA | NA | - |
| **12** | 10.92 | -4.25 | 43.51 | 10.28 | -34.9 | 34.09 | -0.58 | -9.27 | 16.93 | 0.01 | -2.65 | 0.44 | -1e-4 | -5e-3 | 0.05 | 2e-7 | -2e-4 | 2e-5 | 0.99 | 0.85 | 1.00 | 55.05 |
| **13** | 10.28 | 4.98 | 24.82 | 0.64 | -0.51 | 1.75 | -2e-3 | -0.05 | 0.02 | -1e-6 | -2e-4 | 7e-4 | 3e-8 | -3e-6 | 1e-6 | -5e-11 | -1e-9 | 7e-9 | 0.99 | 0.96 | 1.00 | 24.32 |
| **14** | 26.21 | 15.0 | 33.96 | 3.05 | -1.25 | 5.45 | -0.09 | -0.23 | 0.48 | 1e-3 | -0.02 | 4e-3 | -8e-6 | -3e-5 | 4e-4 | 1e-8 | -2e-6 | 9e-8 | 1.00 | 0.98 | 1.00 | 12.07 |
| **14*** | 48.67 | 40.40 | 63.45 | 0.19 | 0.10 | 0.46 | 0 | - | - | 0 | - | - | 0 | - | - | 0 | - | - | 0.78 | 0.65 | 0.85 | 43.26 |
| **15** | 29.59 | 15.59 | 34.95 | 3.50 | 0.37 | 7.22 | -0.1 | -0.34 | 0.39 | 1e-3 | -0.02 | 7e-3 | -8e-6 | -6e-5 | 4e-4 | 1e-8 | -2e-6 | 2e-7 | 1.00 | 0.97 | 1.00 | 17.25 |
| **16** | 31.13 | 23.13 | 37.54 | 3.95 | 2.19 | 6.23 | -0.14 | -0.32 | 4E-3 | 2E-3 | -8E-3 | 7E-3 | -1e-5 | -7e-5 | 1e-4 | 2e-8 | -9e-7 | 2e-7 | 1.00 | 0.99 | 1.00 | 19.47 |
| **17** | 36.99 | 36.70 | 43.17 | 4.43 | 2.73 | 4.59 | -0.18 | -0.2 | -0.07 | 3E-3 | 1E-3 | 4E-3 | -2e-5 | -4e-5 | -5e-6 | 4e-8 | 1e-8 | 1e-7 | 1.00 | 0.99 | 1.00 | 9.29 |
| **18** | -2818 | - | - | 13391 | - | - | -23095 | - | - | 18217 | - | - | -6413 | - | - | 815 | - | - | NA | NA | NA | - |
| **19** | 547.7 | -375.2 | 1675 | -3518 | -10846 | 937 | 7677 | -272 | 25844 | -6835 | -28043 | -427 | 2566 | 280 | 13954 | -338 | -2487 | -45 | NA | NA | NA | - |
| **19*** | 0 | - | - | -659.1 | -1217 | 1463 | 2226 | -9827 | 4505 | -2163 | -5479 | 22930 | 786.8 | -21957 | 2781 | -98.66 | -492 | 7488 | 1.00 | 0.99 | 1.00 | 12.73 |
| **20** | 5.98 | -1.29 | 9.87 | 3.41 | -4.22 | 12.15 | 0.44 | -2.83 | 4.97 | -0.12 | -1.12 | 0.43 | 8E-3 | -0.03 | 0.10 | -1e-4 | -3E-3 | 8e-4 | 0.99 | 0.98 | 0.99 | 1.79 |
| **21** | 9.07 | 8.11 | 10.63 | 1.86 | -0.37 | 3.45 | 0.07 | -0.83 | 1.06 | -0.01 | -0.19 | 0.21 | 4e-4 | -0.02 | 0.01 | -6e-6 | -6e-4 | 9e-4 | 0.99 | 0.99 | 0.99 | -34.8 |
| **21*** | 11.97 | 9.49 | 15.64 | 1.21 | 0.95 | 1.86 | 0 | - | - | 0 | - | - | 0 | - | - | 0 | - | - | 0.94 | 0.91 | 0.97 | 30.67 |
| **22** | 6.73 | 1.25 | 9.62 | 7.06 | 1.34 | 14.19 | -1.96 | -5.20 | 1.26 | 0.31 | -0.48 | 0.91 | -0.02 | -0.07 | 0.064 | 4e-4 | -2E-3 | 2E-3 | 0.99 | 0.99 | 0.99 | -2.97 |
| **23** | 13.07 | 7.72 | 20.71 | 6.36 | -1.51 | 14.87 | -1.09 | -5.79 | 1.69 | 0.15 | -0.33 | 1.26 | -9E-3 | -0.12 | 0.02 | 1e-4 | -1E-3 | 4E-3 | 0.99 | 0.99 | 0.99 | -4.69 |
| **23*** | 20.36 | 15.91 | 25.80 | 1.92 | 1.51 | 3.02 | 0 | - | - | 0 | - | - | 0 | - | - | 0 | - | - | 0.93 | 0.89 | 0.95 | 45.66 |
| **24** | 5.79 | -1.02 | 16.68 | 11.73 | 0.07 | 21.62 | -3.21 | -8.01 | 0.88 | 0.47 | -0.18 | 1.53 | -0.02 | -0.12 | 0.01 | 6e-4 | -4e-4 | 4E-3 | 0.99 | 0.98 | 0.99 | 5.09 |
| **24*** | 11.92 | 10.25 | 14.85 | 3.25 | 2.54 | 3.85 | -0.08 | -0.12 | -0.04 | 0 | - | - | 0 | - | - | 0 | - | - | 0.90 | 0.87 | 0.92 | 12.90 |
| **25** | 10.79 | 1.24 | 18.37 | 8.48 | -0.69 | 21.36 | -1.80 | -7.75 | 1.96 | 0.24 | -0.41 | 1.43 | -0.01 | -0.12 | 0.04 | 3e-4 | -1E-3 | 4E-3 | 0.99 | 0.99 | 0.99 | 2.19 |
| **25*** | 18.98 | 15.07 | 24.51 | 2.12 | 1.68 | 3.10 | 0 | - | - | 0 | - | - | 0 | - | - | 0 | - | - | 0.95 | 0.93 | 0.96 | 43.41 |
| **26** | 41.28 | 27.95 | 66.92 | 9.73 | -1.14 | 21.67 | -1.28 | -4.70 | 0.26 | 0.07 | -0.01 | 0.49 | -1E-3 | -0.02 | 2e-4 | 6e-6 | -1e-6 | 3e-4 | 1.00 | 0.98 | 1.00 | 8.18 |
| **27** | 45.36 | - | - | 1.47 | - | - | 0.49 | - | - | -0.03 | - | - | 8e-4 | - | - | -5e-6 | - | - | NA | NA | NA | - |
| **28** | 2.23 | - | - | 12.97 | - | - | -1.42 | - | - | 0.06 | - | - | -1e-3 | - | - | 8e-6 | - | - | NA | NA | NA | - |
| **29** | 7.54 | - | - | 10.32 | - | - | -0.86 | - | - | 0.03 | - | - | -5e-4 | - | - | 3e-6 | - | - | NA | NA | NA | - |
| **30** | 11.64 | - | - | 5.51 | - | - | -0.45 | - | - | 0.01 | - | - | -3e-4 | - | - | 2e-6 | - | - | NA | NA | NA | - |
| **31** | 22.04 | 15.95 | 38.88 | 5.72 | 0.79 | 9.83 | -0.33 | -0.98 | -2E-3 | 8e-3 | -1e-4 | 0.04 | -8e-5 | -6e-4 | 1e-6 | 3e-7 | -6e-9 | 3e-6 | 1.00 | 0.93 | 1.00 | 24.84 |
| **32** | 18.79 | 12.46 | 37.38 | 6.40 | 0.91 | 10.72 | -0.37 | -1.06 | -1E-3 | 9E-3 | -2e-4 | 0.04 | -9e-5 | -7e-4 | 3e-6 | 3e-7 | -1e-8 | 3e-6 | 1.00 | 0.92 | 1.00 | 26.26 |
| **33** | 47.37 | - | - | -180.3 | - | - | 270.9 | - | - | -153.9 | - | - | 38.43 | - | - | -3.5 | - | - | NA | NA | NA | - |
| **34** | 9.49 | -105.8 | 211.3 | 18.52 | -173.87 | 169.48 | -1.28 | -86.05 | 61.39 | -0.03 | -9.95 | 20.09 | 3E-3 | -1.91 | 0.64 | -3e-5 | -0.01 | 0.05 | 1.00 | 0.97 | 1.00 | 27.66 |
| **34*** | 0 | - | - | 29.96 | 10.48 | 65.35 | -5.99 | -38.85 | 6.42 | 0.76 | -3.88 | 10.00 | -0.03 | -0.91 | 0.68 | 5E-4 | -0.03 | 0.03 | 0.99 | 0.94 | 1 | -30.10 |
| **35** | 7.93 | 3.33 | 14.89 | 24.55 | -83.98 | 32.86 | -5.55 | -9.72 | 24.95 | 0.74 | -3.19 | 1.65 | -0.04 | -0.13 | 0.18 | 1E-3 | -4E-3 | 4E-3 | 1.00 | 0.99 | 1.00 | 6.42 |
| **35*** | 0 | - | - | 35.94 | 22.22 | 40.11 | -10.51 | -14.9 | -2.56 | 1.65 | 0.06 | 3.15 | -0.11 | -0.31 | -6E-3 | 3E-3 | -1E-3 | 0.01 | 1.00 | 0.99 | 1.00 | 15.40 |
| **36** | 36.58 | - | - | -42.87 | - | - | 72.5 | - | - | -28.26 | - | - | 4.27 | - | - | -0.22 | - | - | NA | NA | NA | - |
| **37** | 16.25 | - | - | -0.02 | - | - | 2E-3 | - | - | -1e-5 | - | - | - | - | - | - | - | - | NA | NA | NA | - |
| **38** | 10.25 | -32.6 | 17.76 | -34.98 | -64.39 | 49.03 | 32.43 | -18.1 | 69.45 | -7.24 | -25.14 | 2.85 | 0.55 | -0.18 | 3.47 | -0.01 | -0.15 | 3E-3 | 1.00 | 0.93 | 1.00 | 14.44 |
| **38*** | 0 | - | - | -2.34 | -24.42 | 9.61 | 5.26 | -35.1 | 38.62 | -1.19 | -15.28 | 37.67 | 0.08 | -12.3 | 2.18 | -2E-3 | -0.09 | 1.16 | 1.00 | 0.94 | 1.00 | 14.79 |
| **39** | 0.17 | -16.3 | 5.21 | -4.52 | -23.11 | 25.33 | 9.40 | -6.85 | 30.53 | -2.23 | -11.08 | 8.82 | 0.17 | -0.05 | 1.51 | -3E-3 | -0.06 | 9E-4 | 1.00 | 0.99 | 1.00 | 2.53 |
| **39*** | 0 | - | - | -4.21 | -11.37 | 4.26 | 9.75 | -0.18 | 21.47 | -2.58 | -8.18 | 7.37 | 0.20 | -3.12 | 1.13 | -4E-3 | -0.04 | 0.32 | 1.00 | 0.98 | 1.00 | 7.40 |
| **40** | 10.31 | -34.9 | 18.23 | -33.20 | -64.24 | 55.50 | 32.69 | -20.6 | 71.77 | -7.34 | -26.24 | 3.32 | 0.55 | -0.21 | 3.64 | -0.01 | -0.15 | 4E-3 | 1.00 | 1.00 | 1.00 | 15.20 |
| **40*** | 0 | - | - | -0.09 | -23.21 | 11.74 | 4.70 | -35.6 | 40.10 | -1.08 | -16.11 | 38.26 | 0.08 | -12.5 | 2.31 | -1E-3 | -0.10 | 1.19 | 1.00 | 0.95 | 1.00 | 15.92 |

Note: * refers to sets that have been rescaled considering the intercept with a value of zero.
