# Supplementary material for: Comparative statistical analysis of the release kinetics models for nanoprecipitated drug delivery systems based on poly(lactic-co-glycolic acid)
Source: PLoS One. 2022 Mar 10;17(3):e0264825. doi: 10.1371/journal.pone.0264825 (PMC8912140; doi:10.1371/journal.pone.0264825)
Supplement: S6 File — In order to give significance to the Weibull method, a linear regression was performed between the Korsmeyer-Peppas n parameter and the Weibull beta parameter. (DOCX) [file pone.0264825.s006.docx]

**S6 File. Linear regression of** $\boldsymbol{\beta}$ **vs.** $\boldsymbol{n}$

In order to give significance to the Weibull method, a linear regression was performed between the Korsmeyer-Peppas n parameter and the Weibull beta parameter.


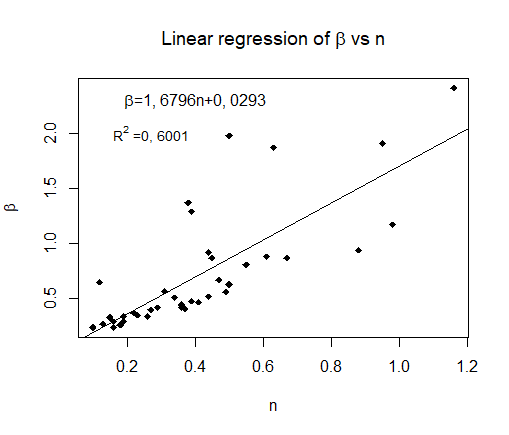


According to the regression, the relationship β=1.6796 n+0.0293 was obtained, so that, when n=0.43, β has a value of 0.75 and when n=0.85, β acquires a value of 1.45.
