# Supplementary material for: Comparative statistical analysis of the release kinetics models for nanoprecipitated drug delivery systems based on poly(lactic-co-glycolic acid)
Source: PLoS One. 2022 Mar 10;17(3):e0264825. doi: 10.1371/journal.pone.0264825 (PMC8912140; doi:10.1371/journal.pone.0264825)
Supplement: Attachment — Submitted filename: Response to Reviewers.pdf [file pone.0264825.s007.pdf]

# Response to Reviewers

For a better reading, reviewers' comments are shown in bold blue, while our response is shown in black.

## 5. Review Comments to the Author

**Responses to the comments of Reviewer #1**

Thank you for forwarding these helpful review reports. We have tried to address REVIEWER #1 suggestions as well as possible and specified in detail below.

**Reviewer #1: Authors deal with the statistical comparison of mathematical models used in order to find which of them best describes the drug release profile from PLGA particles synthesized by nanoprecipitation method. I think the manuscript is very well written overall, very straightforward to follow and understand**
**I will recommend their publication after few corrections/suggestions as detailed below.**
**1. Why authors only consider 40 works and all of them after 2016? Perhaps a clear explanation it would be nice for readers.**

**Response:**

The authors thank this valuable comment. We considered 40 datasets extracted from scientific literature since 2016. This is due to three main reasons listed below.

1. Using the nanoprecipitation method there are hundreds of scientific papers published so far, therefore, to limit the study, the papers from the last 5 years, i.e. since 2016, were selected.
2. In 2016, to the best of our knowledge, a paper referring to the hyperbolic tangent function model was published for the first time (Eltayeb, Stride, Edirisinghe, & Harker, 2016).
3. The authors considered the time of 5 years as sufficient for other authors to consider and use the hyperbolic tangent model and thus collect representative data and results.

For a better understanding of the readers an explanation of this was added in lines 170-172 of the manuscript where it is expressed:

To limit the study, the authors selected scientific articles from 2016. Furthermore, to the best of our knowledge, this was the year in which the mathematical model of hyperbolic tangent function was applied for the first time to evaluate the release profile of core-shell lipid nanoparticles [77].

[77] Eltayeb, M., Stride, E., Edirisinghe, M., & Harker, A. (2016). Electrosprayed

nanoparticle delivery system for controlled release. *Materials Science and Engineering C*, *66*, 138–146. https://doi.org/10.1016/j.msec.2016.04.001

**2. I would also suggest to provide extra 1 or 2 self-made schematizations. For instance: i.- Time line regarding the mathematical models used so far to describes the drug release in similar materials. ii.- a graphical schematization regarding their main results.**

**Response:**

Accordingly to this comment, we have added the following schematization to address point i) as figure #2.

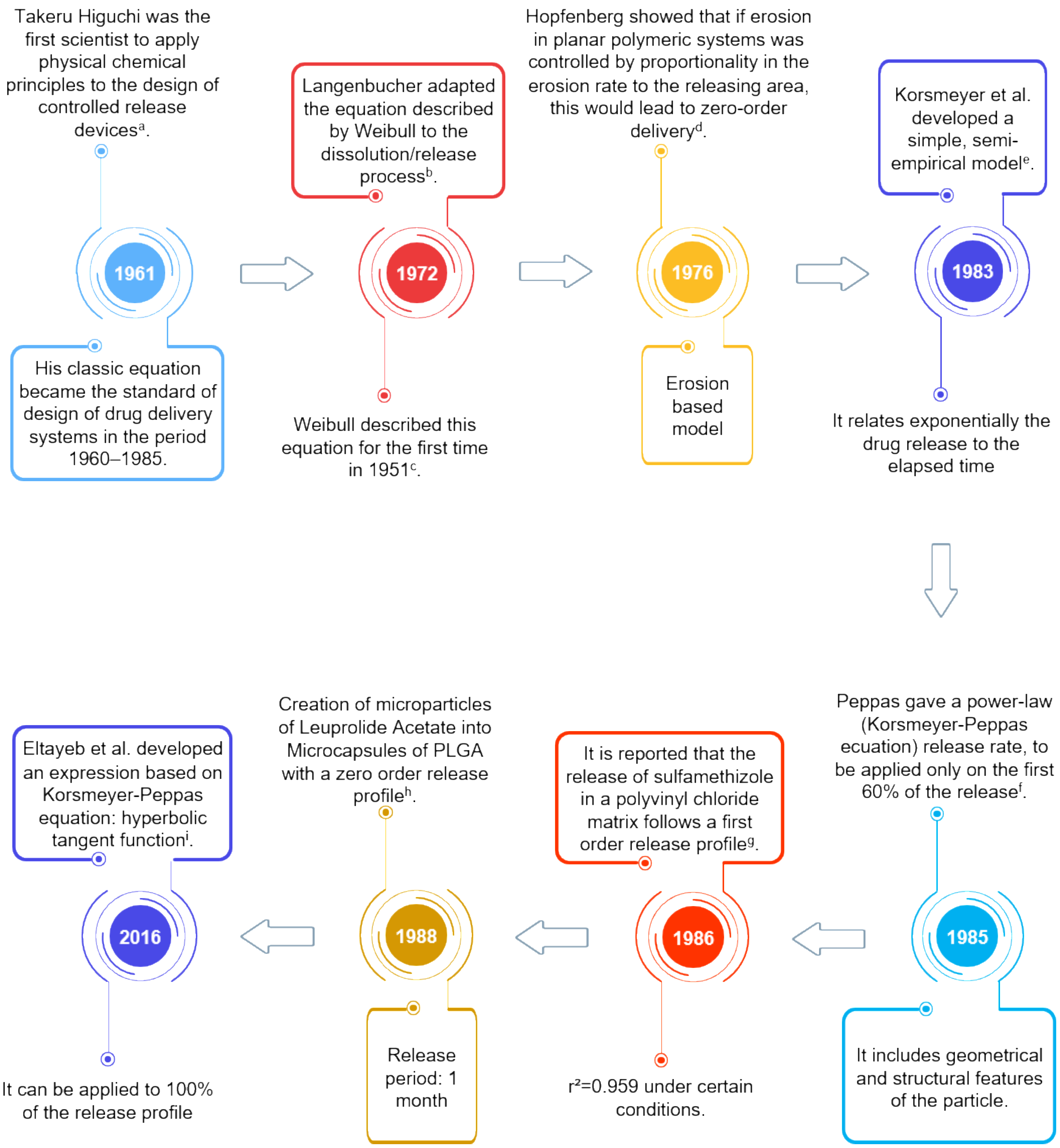

Here the authors show a timeline of the development of the models used in the present study.

In the manuscript, line 200-203 introduce the reader to the aforementioned timeline:

Fig 2 shows a time-line of the development of the mathematical models used in this study.

**Fig 2. Time-line of conventional empirical/semi-empirical models. a. [83]; b. [84]; c. [85]; d. [86]; e. [80]; f. [82]; g. [87]; h. [88]; i. [77].**

**3. Few English sentences are confusing. I will suggest to check/correct the whole manuscript by a native**

**Response:**

Thank you for your suggestion, whole manuscript has been revised by a Native American. All changes are recorded using Track Changes in Microsoft Word

**4. I've particularly don't like the bunch of tables in the manuscript. I would suggest to maintain in the main text the most appealing tables and move the rest to the SI.**

**Response:**

Thank you for your suggestion. We have moved Table 1 and 2 to SI. These tables were selected because they are not main results. Table 1 corresponds to standard equations and Table 2 to scientifical well-known definitions and general equations.

It should be noted that the numbers of the figures and tables in the manuscript and in the SI have changed, these have been updated and also their citations within the text.

**5. Conclusion need to be rewritten, to highlight their main findings.**

**Response:**

Thank you for pointing this out. Conclusion has been rewritten as follow (lines 524-541):

This study demonstrated that, although they are tools that help to understand drug release dynamics, there is no general empirical/semiempirical mathematical expression that can describe, in all cases, the release profile of drugs encapsulated in PLGA nanoparticles synthesized by nanoprecipitation methods. In addition, it was found that although $R^2$ and $R^2_a$ are the most commonly used criteria to determine

whether or not a model fits the data obtained in an experiment, they are not the most appropriate. It was also shown that the Akaike and Bayesian criteria can better reflect the fit of a model since the results are not influenced by the inclusion of new terms or an increase in the complexity of the equation. It was also revealed that the number of observations per set is a limiting factor for the application of different models and the subsequent statistical analysis. Therefore, the Bootstrap resampling technique becomes a very useful technique to solve this drawback. In the specific case of this study, 50% of the sets studied do not meet the requirement of at least 8 observations for the construction of the release curve, therefore these results are not very reproducible and have low statistical significance.

Furthermore, the analysis employed in this project provided significant statistical evidence to consider the Hyperbolic Tangent Function model as the most adequate and general model to describe the drug release kinetics. This model, unlike the mathematical expression of Korsmeyer-Peppas, could be adjusted to the complete release curve. Since it has kinetic parameters, it acquires greater predictive power than the Weibull model. However, a more exhaustive study of this model is required in order to understand the chemistry, physics, and biology behind it.

**Responses to the comments of Reviewer #2**

**Reviewer #2: The manuscript applied the appropriate statistical approaches to compare and summarize the commonly used models for the drug release profile from PLGA particles synthesized by nano-precipitation method.**
**The screening and justification of literature and data are important for the assessment of study outcomes and final conclusion. It will be better to add a flow diagram for the screening procedure and justification/criteria of literature for the systematic research.**

**Response:**

We think this is an excellent suggestion, therefore, we have added Fig. 1 and its citation in the manuscript.

molecular weight or inherent viscosity, among others, were listed. Fig 1 illustrates the search process for the scientific articles selected for this study. The data sets obtained can be found in the supplementary information.

**Fig 1. Flow diagram for the screening procedure and selection criteria for the scientific articles chosen in this study.**

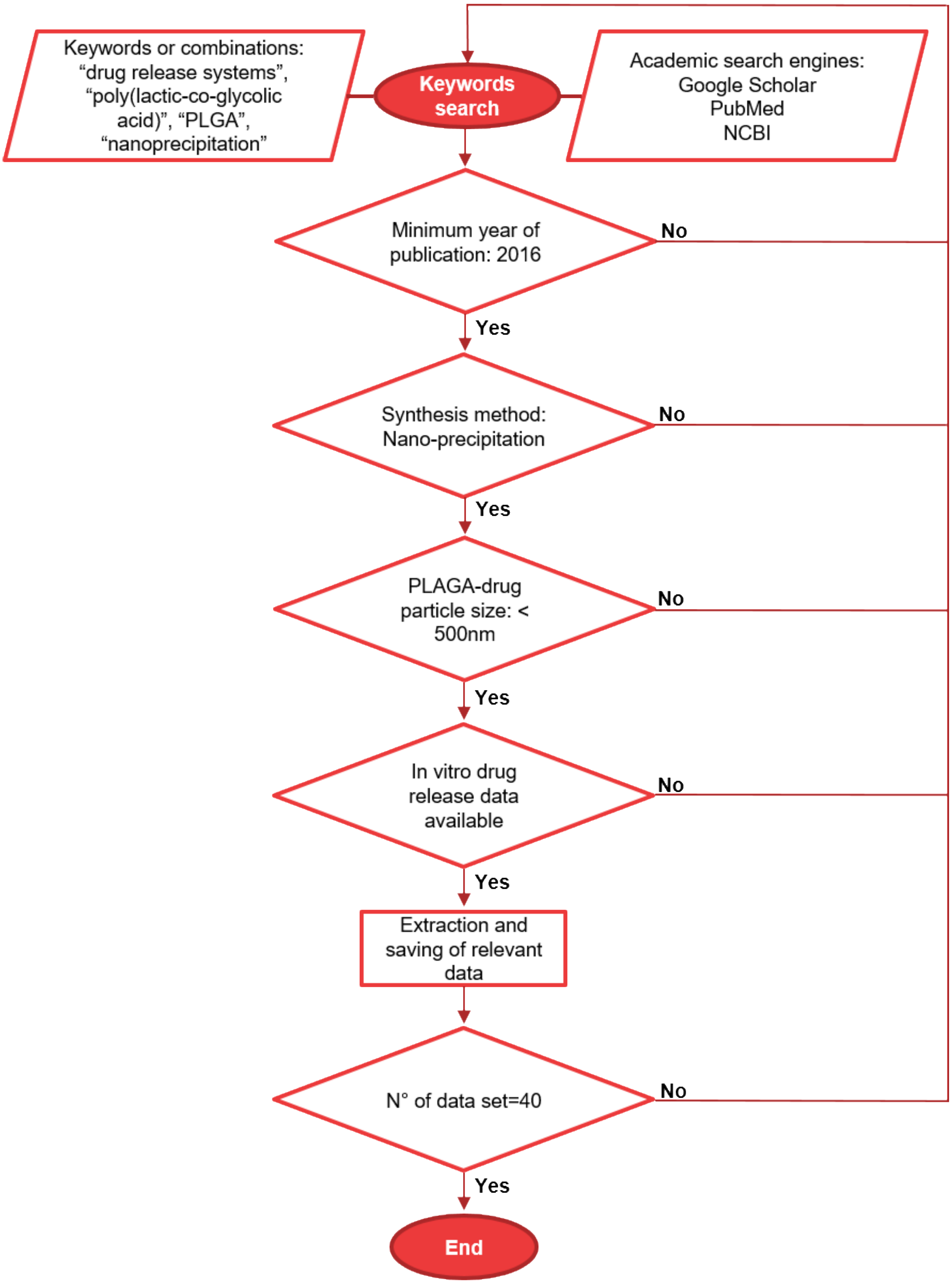


For a better understanding of the readers an explanation of the selection criteria was added in lines 170-172 of the manuscript where it is expressed:

To limit the study, the authors selected scientific articles from 2016. Furthermore, to the best of our knowledge, this was the year in which the mathematical model of hyperbolic tangent function was applied for the first time to evaluate the release profile of core-shell lipid nanoparticles [77].

[77] Eltayeb, M., Stride, E., Edirisinghe, M., & Harker, A. (2016). Electrosprayed nanoparticle delivery system for controlled release. Materials Science and Engineering C, 66, 138–146. https://doi.org/10.1016/j.msec.2016.04.001
